# Spatiotemporal analyses suggest the role of glacial history and the ice‐free corridor in shaping American badger population genetic variation

**DOI:** 10.1002/ece3.6541

**Published:** 2020-07-09

**Authors:** Brett M. Ford, Anna Cornellas, Jennifer A. Leonard, Richard D. Weir, Michael A. Russello

**Affiliations:** ^1^ Department of Biology University of British Columbia Kelowna BC Canada; ^2^ Conservation and Evolutionary Genetics Group Estación Biológica de Doñana (EBD‐CSIC) Seville Spain; ^3^ British Columbia Ministry of Environment and Climate Change Strategy Victoria BC Canada

**Keywords:** ancient DNA, carnivore, peripheral populations, phylogeography, Pleistocene glaciation, *Taxidea taxus*

## Abstract

Recurring glacial cycles through the Quaternary period drastically altered the size and distribution of natural populations of North American flora and fauna. The “southerly refugia model” has been the longstanding framework for testing the effects of glaciation on contemporary genetic patterns; however, insights from ancient DNA have contributed to the reconstruction of more complex histories for some species. The American badger, *Taxidea taxus*, provides an interesting species for exploring the genetic legacy of glacial history, having been hypothesized to have postglacially emerged from a single, southerly refugium to recolonize northern latitudes. However, previous studies have lacked genetic sampling from areas where distinct glacial refugia have been hypothesized, including the Pacific Northwest and American Far North (Yukon, Alaska). In order to further investigate the phylogeographic history of American badgers, we collected mitochondrial DNA sequence data from ancient subfossil material collected within the historical range (Alaska, Yukon) and combined them with new and previously published data from across the species' contemporary distribution (*n* = 1,207). We reconstructed a mostly unresolved phylogenetic tree and star‐like haplotype network indicative of emergence from a largely panmictic glacial refugium and recent population expansion, the latter further punctuated by significantly negative Tajima's *D* and Fu's *Fs* values. Although directionality of migration cannot be unequivocally inferred, the moderate to high levels of genetic variation exhibited by American badgers, alongside the low frequency of haplotypes with indels in the Midwest, suggest a potential recolonization into central North America after the hypothesized ice‐free corridor reopened ~13,000 years ago. Overall, the expanded reconstruction of phylogeographic history of American badgers offers a broader understanding of contemporary range‐wide patterns and identifies unique genetic units that can likely be used to inform conservation of at‐risk populations at the northern periphery.

## Graphical abstract

Recurring glacial cycles through the Quaternary period drastically altered the size and distribution of natural populations of North American fauna, leaving distinct signatures in contemporary gene pools. Here, we combined newly acquired sequencing information from contemporary, historical, and ancient DNA samples of the American badger (*Taxidea taxus*) with exemplar sequences across the species' range (total *n* = 1,207) to reconstruct the phylogeographic history of the species in North America. Recovered patterns of variation revealed little discrete geographic structuring indicative of a largely panmictic glacial refugium and recent population expansion, while also suggesting a potential recolonization into central North America after the hypothesized ice‐free corridor reopened. Photo credit: Emma Walker.

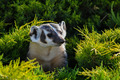

## 1 INTRODUCTION

Understanding how historical processes shape the genetic variation of natural populations has long been of interest to ecologists and evolutionary biologists. Glaciation, in particular, has been extensively studied in the context of population genetic structure, with numerous studies finding concordance between patterns of genetic variation and the historical locations of ice sheets (Hewitt, 1999, 2000, 2004). Early studies, based on fossil evidence, suggested that natural populations survived the harsh environments imposed by glaciation by residing in refugia that constituted geographic regions hospitable for flora and fauna, primarily south of glacial extents (Petit et al., 2003). Indeed, genetic data have corroborated this hypothesis for numerous species, with high levels of genetic diversity at lower latitudes decreasing in a clinal fashion toward northern latitudes, coinciding with the recolonization events that followed glacial retreat (Hewitt, 2004). This “southerly refugia model” (Bennett, Tzedakis, & Willis, 1991) has been the longstanding framework for testing the effects of glaciation on contemporary genetic patterns.

Recently, studies of greater breadth and depth, as well as advances in ancient DNA technologies, have depicted more detailed and oftentimes complex histories for North American populations (Shafer, Cullingham, Côté, & Coltman, 2010; Soltis, Morris, McLachlan, Manos, & Soltis, 2006). Cryptic refugia, which were semihospitable environments within ice sheets (Provan & Bennett, 2008), and a northern refugium in ancient Beringia (Tremblay & Schoen, 1999) have both been suggested to account for the reconstructed patterns of North American populations (Shafer et al., 2010; Soltis et al., 2006). Indeed, genetic studies have identified “hot spots” of genetic diversity and discrete genetic variation for populations at the northern extent of species' ranges, suggesting a refugial history (Rowe, Heske, Brown, & Paige, 2004; Shafer, Côté, & Coltman, 2011). However, patterns of genetic diversity indicating potential glacial refugia can be obscured by complex scenarios, such as admixture between lineages from separate refugia (Petit et al., 2003), genetic structure within refugia (i.e., refugia within refugia; Gómez & Lunt, 2007), and deeper historical associations (Lovette & Bermingham, 1999). Identifying the locations of glacial refugia and accounting for these alternative scenarios can also directly inform conservation management by identifying lineages of distinct ecological and evolutionary significance (Bhagwat & Willis, 2008; Hampe & Petit, 2005).

One interesting species to explore the genetic legacy of glacial history is the American badger (*Taxidea taxus*). As a semifossorial mustelid, American badgers are typically restricted to dry grassland–shrubland habitats, where they can burrow in silty, sandy‐loam soils and catch fossorial prey (Committee on the Status of Endangered Wildlife in Canada, 2012). Despite its habitat specialization, the American badger's high dispersal capabilities have enabled colonization to diverse areas throughout much of central and western North America (Committee on the Status of Endangered Wildlife in Canada, 2012; Kierepka & Latch, 2016). Thus, while habitat specialization suggests that badgers should have resided in one or multiple refugia, their dispersal capacity and extensive species' range strongly suggest the potential for the latter (Shafer et al., 2010). It has been hypothesized that glaciation displaced badgers into a single, southerly refugium from which the species recolonized northern latitudes, with isolation by major geographic barriers leading to the recognition of four subspecies (*T. t. jacksoni, T. t. taxus, T. t. berlandieri*, and *T. t. jeffersonii*; Kierepka & Latch, 2016; Long, 1972). Phylogeographic studies have partially supported this hypothesis, providing evidence for fragmented units with low genetic diversity at the northern periphery (Ethier, Lafleche, Swanson, Nocera, & Kyle, 2012), and connected populations with high levels of gene flow at the center of the species' range (Kierepka & Latch, 2016). However, a lack of genetic sampling from areas where distinct glacial refugia have been hypothesized (e.g., the Pacific Northwest and Alaska, the latter a location where badgers resided historically) has hindered a complete picture of how postglacial colonization has affected contemporary genetic patterns. Understanding the relative impact of postglacial colonization is essential, especially for the management of endangered populations at the periphery; in Canada, American badgers are currently listed as endangered (*T. t. jacksonii*, *T. t. jeffersonii*—Eastern population, *T. t. jeffersonii*—Western population) or of special concern (*T. t. taxus*) (Committee on the Status of Endangered Wildlife in Canada, 2012).

Here, we aimed to gain a broader perspective of the historical influences that shaped the genetic variation of American badger populations across the entire species' range. To do so, we collected mitochondrial DNA sequence data from ancient subfossil material collected within the historical range of American badgers in Alaska (USA) and the Yukon (Canada), and paired these with new data from the contemporary range in the American West and Pacific Northwest where coverage has been previously thin or lacking. We combined these data with those previously published from across the species distribution (Ethier et al., 2012; Ford, Weir, Lewis, Larsen, & Russello, 2019; Kierepka & Latch, 2016), producing the most comprehensive dataset to date, covering the American badger's historical and contemporary ranges. We used these data to reconstruct the phylogeographic history of the American badger to specifically test alternative hypotheses of (1) a single refugium, which would be generally evidenced by a star‐like topology showing little differentiation among a few common haplotypes surrounded by rare haplotypes, or (2) multiple refugia, which would be partially supported by well‐defined geographic groups with highly divergent haplotypes.

## 2 METHODS

### 2.1 Sample collection

**FIGURE 1:**
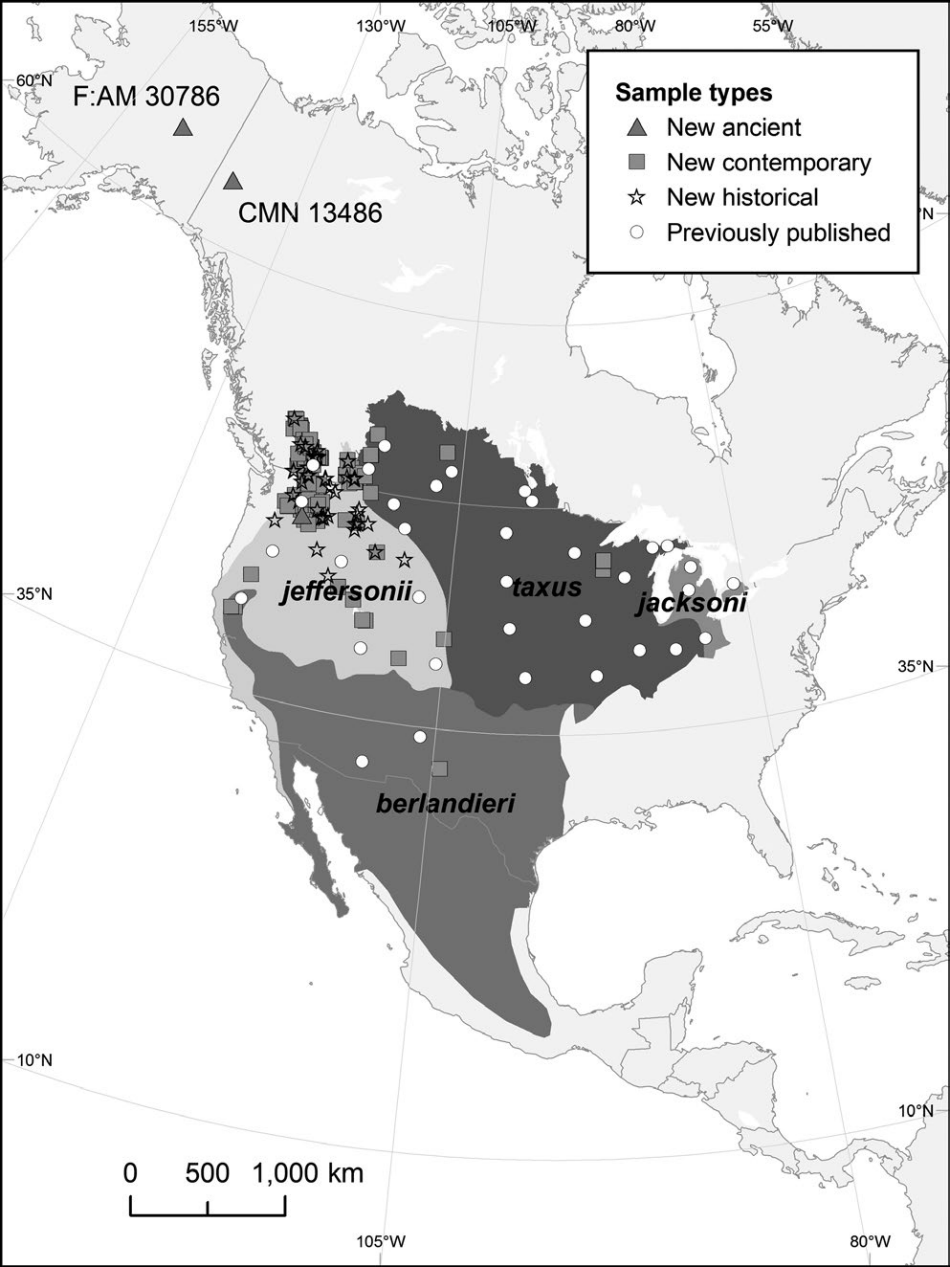
Sample distribution map of American badgers used in the current study. Subspecies boundaries are shaded, and sample type is represented by different symbols defined in the inset. Previously published samples are plotted as a single point representing sampling units

We sampled ancient subfossils (*n* = 13) with estimated ages dating to the late Wisconsin (>12,000 ybp) with provenances in Alaska, USA, and Yukon, Canada, from the American Museum of Natural History and the Canadian Museum of Nature (Figure 1; Appendix S1; Table S1). Methods to prepare subfossil bones for DNA extraction followed those described in Lippold et al. (2011). Briefly, a Dremel^®^ tool fitted with a cutoff wheel was used to remove surface contaminants from bones. A 0.25 g sample was then removed from each specimen, taking caution not to disturb bone processes used for species identification. Samples were covered with aluminum foil and pulverized with a mortar and pestle before DNA extraction. All surfaces and equipment were thoroughly cleaned with bleach solutions between each sample.

Additionally, we collected claw powder samples from 74 museum specimens accessioned across five institutions [Royal British Columbia Museum (RBCM), Burke Museum of Natural History and Culture (BMNHC), Beaty Biodiversity Museum (BBM), Charles R. Conner Museum (CRCM), James R. Slater Museum of Natural History (PSM), Phillip L. Wright Zoological Museum (PLWZM)] from British Columbia (*n* = 31), Washington (*n* = 20), Idaho (*n* = 9), and Montana (*n* = 14) (Appendix S1). Samples were collected following methods described in Casas‐Marce, Revilla, and Godoy (2010). Briefly, a Dremel^®^ bit tool (1.5‐2mm diameter) instrument was used to drill powder from the base of a single foreclaw. Surfaces and tools were cleaned with bleach between collection of each sample. Powder samples were stored dry in 2 ml centrifuge tubes until DNA extraction.

Lastly, we subsampled tissue from 12 roadkill specimens accessioned at the BC Ministry of Environment and Climate Change Strategy originally collected in 2015 from California (*n* = 2), Colorado (*n* = 2), New Mexico (*n* = 1), Oregon (*n* = 1), Saskatchewan (*n* = 1), Utah (*n* = 2), and Wisconsin (*n* = 3).

### 2.2 Data collection

Genomic DNA was extracted from contemporary samples using the Nucleospin Tissue Kit (Macherey‐Nagel). DNA extractions from historical samples were conducted using a MinElute Kit (Qiagen) and the protocol in Jensen et al. (2018) within the dedicated Historical DNA Analysis Facility at the University of British Columbia Okanagan, Canada. Genomic DNA was extracted from subfossil specimens using a phenol–chloroform method (Losey et al., 2013) in the ancient DNA laboratory at the Estación Biológica de Doñana, Spain. All extractions from historical and ancient specimens were conducted in batches of 16 tubes including three or four negatives that were carried through all steps, including PCR amplification, when an additional two negatives were added per batch. Different species were included in ancient DNA extraction batches such that two samples of the same species were never in adjacent tubes.

Haplotypic data were collected from a fragment of the D‐loop from the mitochondrial genome so as to make use of the expansive range‐wide data from several previously published studies (Table 1; Ethier et al., 2012; Ford et al., 2019; Kierepka & Latch, 2016). We used the primer set designed in Ford et al. (2019) targeting a ~600 basepair fragment at the 5′ end of the D‐loop as well as three internal primer sets to amplify ~200 basepair fragments for the more degraded DNA samples. Fragments were PCR amplified in 15 μl volumes containing 1X PCR Buffer (Applied Biosystems; 150 mM Tris‐HCl, pH 8.0, 500 mM KCl), 0.2 mM dNTPs, 0.2 mg/ml bovine serum albumin, 0.67 μM of each primer, and 0.5 U of AmpliTaq Gold DNA Polymerase (Applied Biosystems). For ancient samples, we used 0.8 mg/ml of bovine serum albumin (BSA) or rabbit serum album (RSA). Cycling conditions for all fragments included an initial denaturation step at 94°C for 10 min, followed by five cycles of 95°C for 45 s, 55°C for 45 s and 72°C for 45 s, then 35 cycles of 95°C for 45 s, 48°C for 45 s, 72°C for 1 min, and then a final extension at 72°C for 10 min. Ramp times between denaturation and annealing and between annealing and extension were slowed to 1°C/s. All reactions were checked on agarose gels stained with SYBR Safe (Invitrogen), and all appropriately sized bands were directly sequenced in both directions with the same primers used in the PCR. To assure that accurate DNA sequencing information was recovered from ancient samples, each DNA fragment was independently amplified and sequenced at least twice. PCR products were purified using magnetic beads (Rohland & Reich, 2012) and Sanger sequenced by Macrogen. Sequences were visualized and edited using Sequencher 5.0 (Gene Codes Corporation).

**TABLE 1:** Sample distribution and diversity indices based on a fragment of the mtDNA D‐loop for American badgers across the species' range

Sampling Unit	Code	*N*	Number of Haplotypes	Haplotype Diversity (*H*_d_ ± *SE*)	Nucleotide Diversity (*π* ± *SE*)	Pairwise Difference	Source
British Columbia	BC	127	6	0.670 ± 0.031	0.006 ± 0.004	2.66 ± 1.43	F: 101, N: 26
‐ Cariboo	CR	26	1	0.000 ± 0.000	0.000 ± 0.000	0.00 ± 0.00	
‐ Thompson	TH	21	4	0.576 ± 0.099	0.005 ± 0.003	2.15 ± 1.24	
‐ Nicola	NI	6	2	0.600 ± 0.129	0.002 ± 0.002	0.60 ± 0.55	
‐ Okanagan	OK	40	4	0.631 ± 0.060	0.003 ± 0.002	1.38 ± 0.87	
‐ East Kootenay	EK	34	4	0.590 ± 0.071	0.007 ± 0.004	2.70 ± 1.47	
Washington	WA	46	13	0.818 ± 0.038	0.007 ± 0.004	2.61 ± 1.42	K: 1, F: 39, N: 6
Oregon	OR	14	9	0.934 ± 0.045	0.011 ± 0.006	4.23 ± 2.23	K: 13, N: 1
Alberta	AL	51	15	0.674 ± 0.074	0.007 ± 0.004	3.01 ± 1.59	K: 5, E: 41, F: 5
Idaho	ID	29	15	0.941 ± 0.022	0.008 ± 0.005	3.15 ± 1.68	K: 20, F: 4, N: 5
Montana	MT	78	19	0.703 ± 0.055	0.007 ± 0.004	3.04 ± 1.60	K: 55, E: 12, F: 3, N: 8
Saskatchewan	SK	54	13	0.801 ± 0.037	0.007 ± 0.004	2.86 ± 1.53	K: 26, E: 27, N: 1
Manitoba	MB	60	14	0.854 ± 0.030	0.008 ± 0.005	3.55 ± 1.83	K: 4, E: 56
Wyoming	WY	8	6	0.929 ± 0.084	0.007 ± 0.004	3.75 ± 2.11	K: 8
Utah	UT	44	16	0.901 ± 0.022	0.009 ± 0.005	5.00 ± 2.48	K: 42, N: 2
Colorado	CO	6	6	1.00 ± 0.096	0.009 ± 0.006	3.40 ± 2.02	K: 4, N: 2
New Mexico	NM	12	6	0.758 ± 0.122	0.009 ± 0.005	3.56 ± 1.95	K: 11, N: 1
North Dakota	ND	58	16	0.783 ± 0.044	0.004 ± 0.003	2.44 ± 1.34	K: 58
South Dakota	SD	78	24	0.881 ± 0.021	0.006 ± 0.004	3.55 ± 1.82	K: 78
Nebraska	NE	60	23	0.842 ± 0.044	0.005 ± 0.003	2.73 ± 1.47	K: 60
Oklahoma	OL	12	7	0.833 ± 0.100	0.005 ± 0.003	2.73 ± 1.56	K: 12
Kansas	KS	31	14	0.843 ± 0.060	0.006 ± 0.003	3.44 ± 1.80	K: 31
Minnesota	MN	67	17	0.774 ± 0.045	0.006 ± 0.004	3.69 ± 1.89	K: 67
Iowa	IA	59	10	0.817 ± 0.034	0.007 ± 0.004	3.83 ± 1.95	K: 59
Missouri	MO	8	4	0.643 ± 0.184	0.005 ± 0.003	2.89 ± 1.70	K: 8
Wisconsin	WI	76	9	0.530 ± 0.064	0.004 ± 0.002	2.09 ± 1.18	K: 73, N: 3
Illinois	IL	11	8	0.927 ± 0.067	0.007 ± 0.004	3.93 ± 2.13	K: 11
Indiana	IN	4	3	0.833 ± 0.222	0.004 ± 0.004	2.50 ± 1.69	K: 4
Ontario	ON	26	2	0.077 ± 0.070	0.000 ± 0.000	0.08 ± 0.15	E: 26
Upper Peninsula	UP	35	5	0.664 ± 0.048	0.003 ± 0.002	1.32 ± 0.84	K: 18, E: 17
Lower Peninsula	LP	118	5	0.642 ± 0.025	0.001 ± 0.001	0.51 ± 0.43	K: 99, E: 19
Ohio	OH	28	4	0.426 ± 0.107	0.003 ± 0.002	1.73 ± 1.04	K: 28
California	CA	3	3	NA	NA	NA	K: 1, N: 2
Arizona	AZ	2	2	NA	NA	NA	K: 2
Alaska	AK	1	1	NA	NA	NA	N: 1
Yukon	YT	1	1	NA	NA	NA	N: 1

Note

K = Kierepka and Latch (2016), E = Ethier et al. (2012), *F* = Ford et al. (2019), *N* = new to this study. Populations with low sample size (*n* < 4; California, Arizona, Alaska, and Yukon) were excluded from genetic diversity and SAMOVA analyses.

We combined these newly collected data with homologous mtDNA D‐loop sequences deposited in GenBank for 1160 American badgers originally sampled from 2001 to 2016 throughout North America for which locality information was available (Table 1; Ethier et al., 2012; Ford et al., 2019; Kierepka & Latch, 2016).

### 2.3 Phylogenetic analysis

All newly acquired and previously published sequences from Ford et al. (2019) and Ethier et al. (2012) were trimmed at the 5′ end to align with sequences from Kierepka and Latch (2016), and 3′ terminal gaps were coded as missing data. All sequences were aligned using default parameters in MUSCLE (Edgar, 2004) as implemented in GENEIOUS 10.1.2 (Kearse et al., 2012).

To reconstruct relationships between mtDNA haplotypes, Bayesian phylogenetic analysis was conducted using MrBayes 3.2 (Ronquist & Huelsenbeck, 2003). A previously identified 26‐bp indel was coded as a fifth character state following Kierepka and Latch (2016). The best fit model of nucleotide substitution (GTR + G + I) was determined using jmodeltest2 (Darriba, Taboada, Doallo, & Posada, 2012) and the Bayesian information criterion (BIC). Overlapping D‐loop sequence from the previously published European badger (*Meles meles*) mitochondrial genome was used as an outgroup. The analysis ran four simultaneous chains for 2.0 × 10^6^ total generations, each using a random tree as a starting point, the default heating scheme, and saving a tree every 200 generations for a total 10,000 trees. The first 1,000 trees were discarded as burn‐in samples and the remaining 9,000 trees were used to construct a consensus tree and derive posterior probability values.

A haplotype network was constructed using statistical parsimony, as implemented in TCS (Clement, Posada, & Crandall, 2000). The dissimilarity matrix from the TCS output was used to visualize the haplotype network using Hapstar 0.7 (Teacher & Griffiths, 2011).

### 2.4 Haplotypic variation and geographic structure

To define populations for downstream analyses, individuals were grouped into sampling units based on their state or province of origin, except sequences from British Columbia, which were grouped into the five sampling units (Cariboo, Thompson, Nicola, Okanagan, and East Kootenay) following Ford et al. (2019). Specific geographic coordinates for each individual were not provided in Kierepka and Latch (2016) or Ethier et al. (2012); therefore, the latitude and longitude of the centroid for each state, province, or distribution (e.g., units at the periphery where badgers are not found throughout the entire state or province) were used for the geographic coordinates of sampling units following Kierepka and Latch (2016). Estimates of haplotype diversity (*H*_d_), nucleotide diversity (*π*), and pairwise difference were calculated for each sampling unit in Arlequin 3.5 (Excoffier, Laval, & Schneider, 2005). A least‐squares linear regression was used to test for an association between the peripherality of a sampling unit and its genetic diversity using function *lm()* as implemented in R (R Core Team, 2018) with haplotype diversity (*H*_d_) as the response variable and distance to the center (in km) as the explanatory variable. The center was calculated by averaging the latitude and longitude of centroids for all states, provinces, and peripheral ranges inhabited by American badgers in North America. Distance from the center was calculated for each sampling unit for which we had adequate haplotype diversity estimates (≥5 haplotypes). A least‐squares linear regression was also employed to test for an association between the latitude of a sampling unit and its haplotype diversity again using function *lm()* as implemented in R (R Core Team, 2018) with *H*_d_ as the response variable and latitude as the explanatory variable.

To measure differentiation between sampling units across the species' distribution, we conducted a spatial analysis of molecular variance (SAMOVA 1.0; Dupanloup, Schneider, & Excoffier, 2002). SAMOVA defines groups of populations by identifying the configuration that maximizes the amount of genetic variance explained among differentiated groupings (Dupanloup et al., 2002). The optimal configuration of groups was tested from *K* = 2 to *K* = 15, using 100 simulated annealing steps, as suggested in the manual (Dupanloup et al., 2002). We chose the optimal configuration based on the grouping that maximized the total amount of genetic variance between groups (*ϕ*_CT_).

### 2.5 Demographic history

To test for evidence of population expansion, we used two neutrality tests, Tajima's *D* (Tajima, 1989) and Fu's *Fs* (Fu, 1997), for each of the SAMOVA groupings and for the entire contemporary dataset. Tajima's *D* and Fu's *Fs* are expected to be significantly negative for populations that have undergone a recent expansion. We tested for significance of the two summary statistics using 1,000 random permutations implemented in Arlequin 3.5 (Excoffier et al., 2005).

To infer changes in effective population size over time, a Bayesian skyline plot analysis (Drummond, Rambaut, Shapiro, & Pybus, 2005) was implemented in BEAST 2.5.2 (Bouckaert et al., 2014) using the best fit model of nucleotide substitution (GTR + G + I) as described above. We ran three chains for 1 × 10^7^ Markov chain Monte Carlo (MCMC) generations, using a clock rate of 1.0 × 10^–7^ substitutions per base per year following Kierepka and Latch (2016) and informed by Molak, Suchard, Ho, Beilman, and Shapiro (2015). We discarded the first 10% of generations as burn‐in and Bayesian skyline plot analysis was implemented in TRACER 1.7 (Rambaut, Drummond, Xie, Baele, & Suchard, 2018).

## 3 RESULTS

### 3.1 Data collection

Two of 13 ancient subfossils collected in Alaska, USA, and Yukon, Canada, yielded the entire D‐loop fragment [one from Alaska (F:AM 30786) and one from Yukon (CMN 13486)], with five additional samples recovering partial sequences; only the two full sequences were retained for analyses. High‐quality mtDNA D‐loop sequences were obtained from 45 of 74 museum specimens and all 12 new contemporary samples. Combined with previously published mitochondrial DNA sequence data from the species' range, our final dataset included 1,207 individuals (Figure 1; Table 1; Table S1).

### 3.2 Phylogenetic and network analyses

**FIGURE 2:**
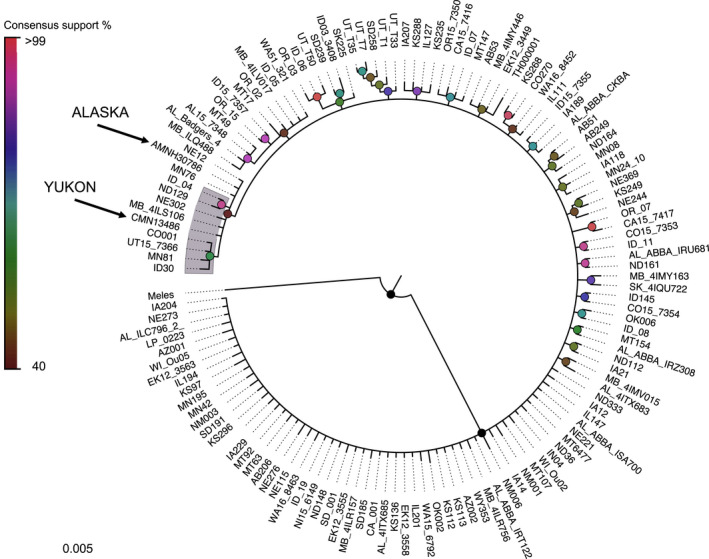
Bayesian phylogenetic tree of all recovered mtDNA D‐loop haplotypes of American badgers across the contemporary and historical range. The European badger (*Meles meles*) was used an outgroup to root the tree. Haplotypes recovered from the ancient subfossil specimens are indicated by asterisks. Shaded haplotypes included the 26‐basepair indel. Bayesian posterior probabilities are indicated according to the inset color bar

A total of 118 haplotypes were recovered across all sampling units, four of which were new to this study. The Bayesian phylogenetic tree revealed little structure, consistent with the findings of Kierepka and Latch (2016). The samples from Alaska and Yukon formed a weakly supported clade with haplotypes found in the Canadian Prairies and the American Midwest (Figure 2).

**FIGURE 3:**
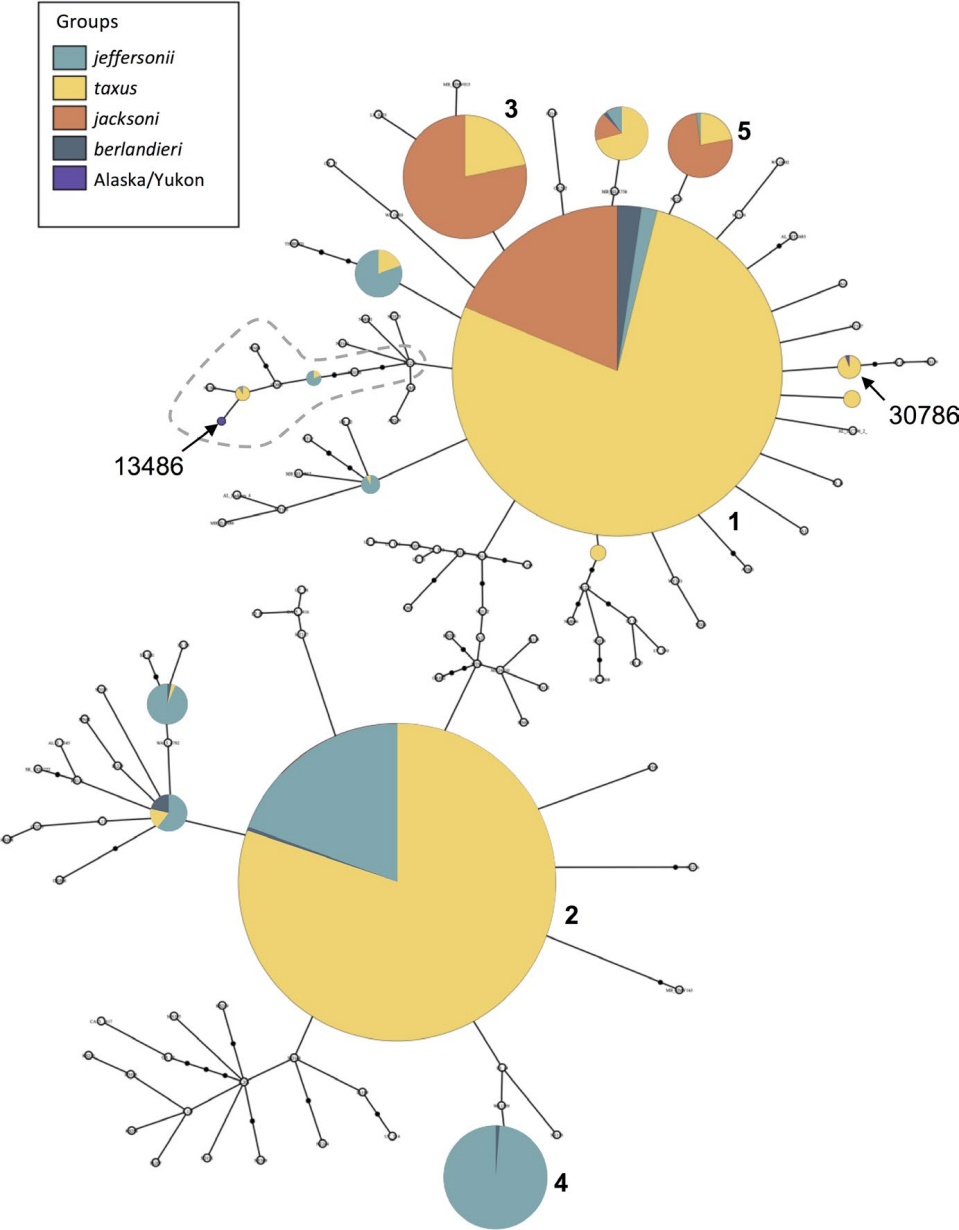
Haplotype network based on a fragment of the mtDNA D‐loop from American badger sampling units across the species' range. Each circle represents a unique haplotype, with the size corresponding to the relative number of individuals with each haplotype. Colors designate the four current subspecies designations in relation to sampling units in the Pacific Northwest [*T. t. jeffersonii*] and eastern Beringia (Alaska/Yukon). Open circles represent haplotypes with 10 or fewer individuals. Closed, black circles represent unsampled transitions between recovered haplotypes. The most common haplotypes are numbered 1–5. Haplotypes recovered from ancient specimens are labeled with museum identification numbers. Sequences with a 26‐basepair indel are highlighted by the gray dashed line

Similarly, the haplotype network revealed no discrete associations between haplotypes and geographic locations for sampling units across the species' range, although the five most common haplotypes were found in high proportions in certain localities, especially for subspecies with sampling units at the periphery, such as *T. t. jeffersonii* and *T. t. jacksoni* (Figure 3). One haplotype was the most common across all badger sampling units (Haplotype 1), found in all four current subspecies designations; the four other most common haplotypes were found at varying frequencies across the continent (Figure 3). *T. t. taxus* shared haplotypes with all other subspecies, but subspecies at opposite ends of the species' distribution shared very little haplotypic variation (e.g., *T. t. jeffersonii* and *T. t. jacksonii*).

### 3.3 Haplotypic variation and geographic structure

**FIGURE 4:**
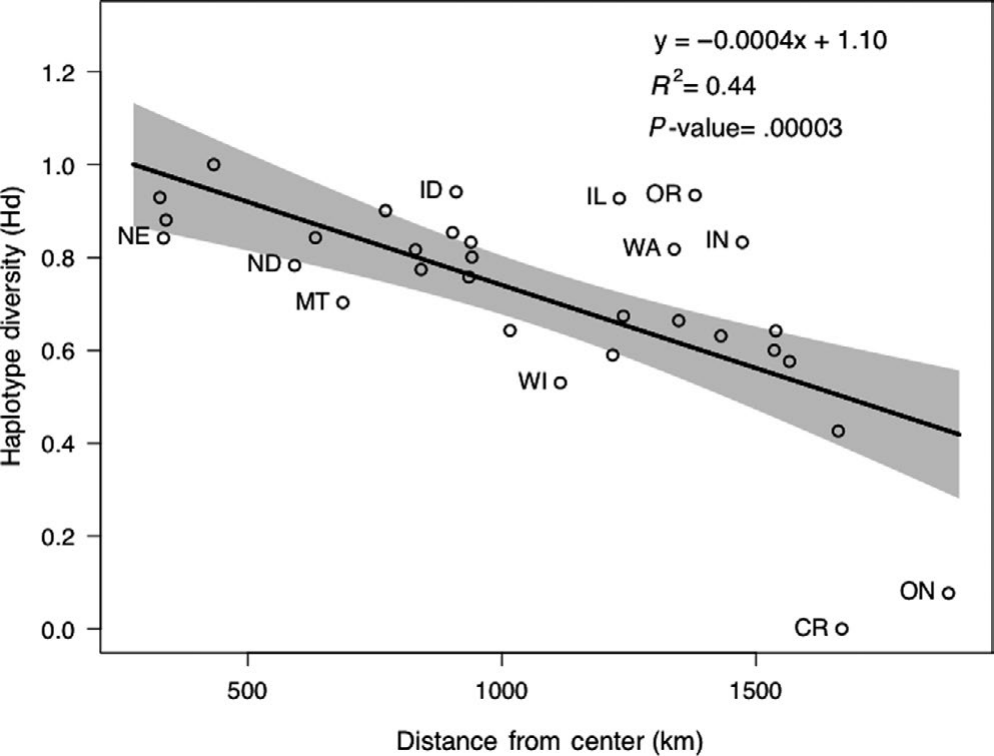
Least‐square regression of the distance from center (kilometers) versus haplotype diversity (*H*_d_). Gray shading depicts the 95% confidence interval. Sampling units ≤ 5 individuals were excluded from analysis. Outlier sampling units, with residuals > 1.0, are labeled with sampling unit abbreviations

Variation in haplotype diversity was large, ranging from 0.000 ± 0.000 in BC‐Cariboo to 1.00 ± 0.096 in Colorado (Table 1). Haplotype diversity was significantly associated with distance to center, decreasing as sampling units were closer to the periphery (*R* ^2^ = .44; *p < *.001; Figure 4). Haplotype diversity generally decreased with an increase in latitude, although not significantly (*R* ^2^ = .10; *p* = .08, data not shown).

**FIGURE 5:**
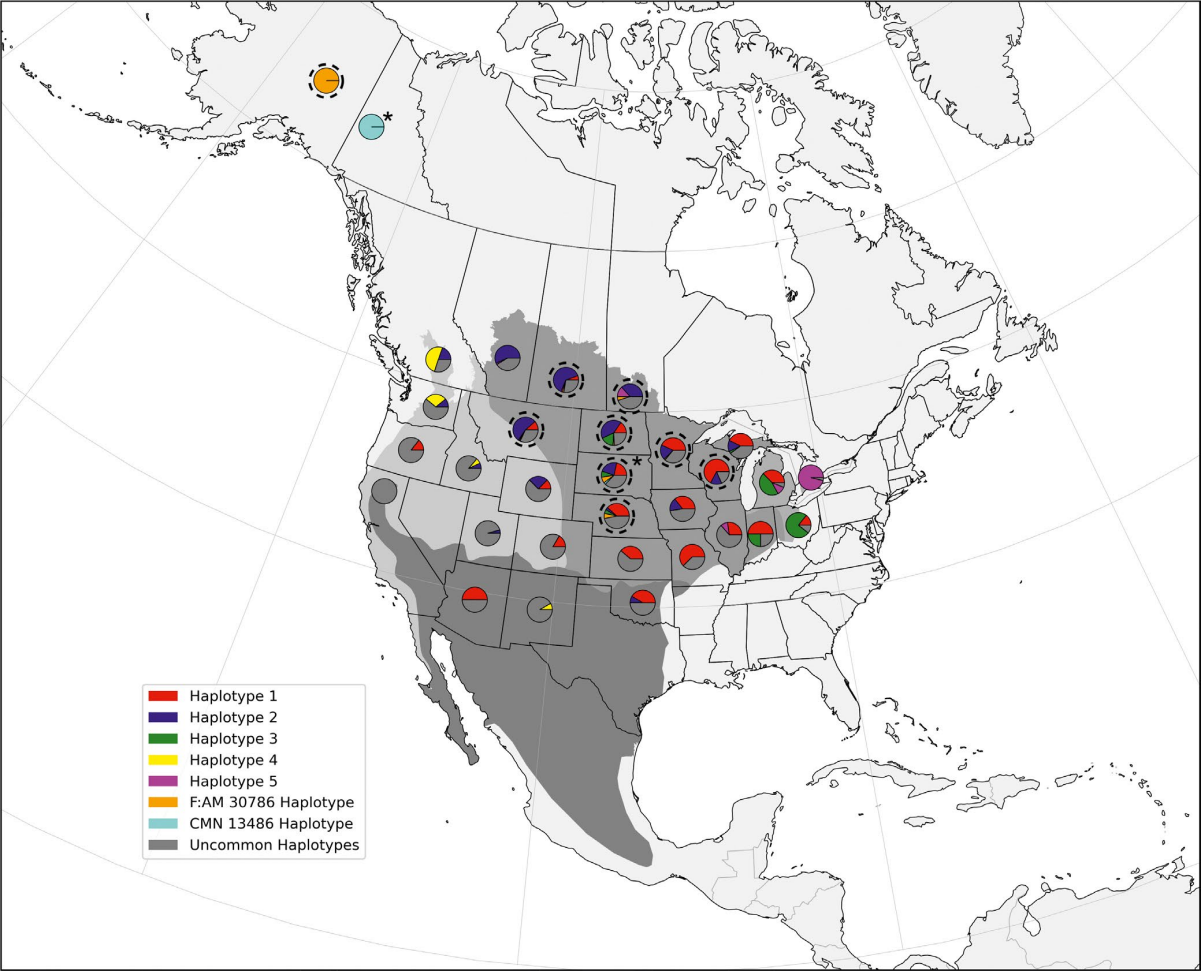
Distribution of the most common haplotypes and haplotypes recovered from ancient specimens across the American badger range. Locations of pie charts represent sampling units. Subspecies boundaries are shaded. Dotted circles indicate locations that shared the haplotype possessed by the ancient specimen sampled in Alaska (F:AM 30786); asterisk indicates the location that shared the haplotype possessed by the ancient specimen sampled in the Yukon (CMN 13486)

Haplotypes recovered from ancient badgers from Alaska and the Yukon were exact matches to haplotypes in the Canadian “Prairie provinces” of Saskatchewan and Manitoba, as well as in Montana and the American Midwestern states of North Dakota, South Dakota, Nebraska, Minnesota, and Wisconsin (Figure 5). Of particular note, the Yukon sample possessed a haplotype with a distinguishing 26 bp indel that has been detected previously in the American West and Midwest (Figure 2; Kierepka & Latch, 2016).

SAMOVA analyses estimated an optimal configuration of populations at *K* = 2, where the BC‐Cariboo and BC‐Nicola separated from all other sampling units throughout the species' range (Table 2), with 39.26% of variation explained among the two groups. The percent of variation among groups and *ϕ*_CT_ gradually decreased as the number of groups considered (*K*) increased. The next configuration that explained the most variation between groups further separated BC‐Cariboo and BC‐Nicola units into their own groups at *K* = 3 (Table 2). Consistent with results from Kierepka and Latch (2016), we found that the Lower Peninsula, Ohio, and Ontario samples grouped together at *K* = 5 relative to all other sampling units in North America, besides those identified in British Columbia (Table 2).

**TABLE 2:** SAMOVA groupings for *K* = 2 to *K* = 10

*K*	Groupings	*ϕ*_SC_	*ϕ*_ST_	*ϕ*_CT_	% var among groups
2	CR, NI | TH, OK, WA, OR, EK, AL, ID, MT, SK, MB, WY, UT, CO, NM, ND, SD, NE, OL, KS, MN, IA, MO, WI, IL, IN, ON, UP, LP, OH	0.252	0.546	0.393	39.26
3	CR | NI | TH, OK, WA, OR, EK, AL, ID, MT, SK, MB, WY, UT, CO, NM, ND, SD, NE, OL, KS, MN, IA, MO, WI, IL, IN, ON, UP, LP, OH	0.254	0.534	0.376	37.56
4	CR, TH, OK | NI | IN | WA, OR, EK, AL, ID, MT, SK, MB, WY, UT, CO, NM, ND, SD, NE, OL, KS, MN, IA, MO, WI, IL, ON, UP, LP, OH	0.217	0.489	0.348	34.76
5	CR | TH, OK | NI | ON, LP, OH | WA, OR, EK, AL, ID, MT, SK, MB, WY, UT, CO, NM, ND, SD, NE, OL, KS, MN, IA, MO, WI, IL, IN, UP	0.134	0.428	0.339	33.91
6	CR | TH, OK | NI | IN | ON, LP, OH | WA, OR, EK, AL, ID, MT, SK, MB, WY, UT, CO, NM, ND, SD, NE, OL, KS, MN, IA, MO, WI, IL, UP	0.134	0.425	0.336	33.56
7	CR, TH, OK | NI | CO | MO | IN | ON, LP, OH | WA, OR, EK, AL, ID, MT, SK, MB, WY, UT, NM, ND, SD, NE, OL, KS, MN, IA, WI, IL, UP	0.133	0.417	0.328	32.76
8	CR | TH, OK | NI | CO | NM | IN | ON, LP, OH | WA, OR, EK, AL, ID, MT, SK, MB, WY, UT, ND, SD, NE, OL, KS, MN, IA, MO, WI, IL, UP	0.134	0.414	0.323	32.28
9	CR, TH, OK | NI | WY | CO | MO | IL | IN | ON, LP, OH | WA, OR, EK, AL, ID, MT, SK, MB, UT, NM, ND, SD, NE, OL, KS, MN, IA, WI, UP	0.135	0.405	0.313	31.26
10	CR | TH, OK | NI | WA | AL, MT, SK | WY | CO | IN | ON, LP, OH | OR, EK, ID, MB, UT, NM, ND, SD, NE, OL, KS, MN, IA, MO, WI, IL, UP	0.064	0.353	0.309	30.86

Note

Acronyms as in Table 1.

### 3.4 Demographic history

**FIGURE 6:**
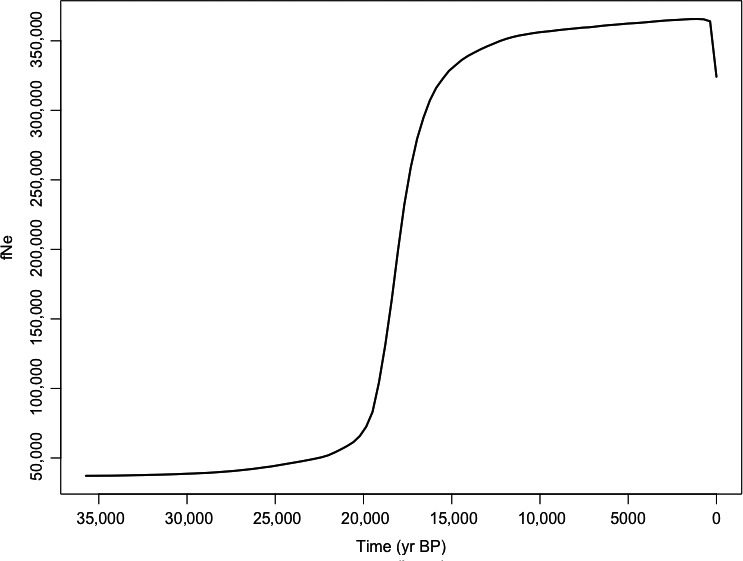
Bayesian skyline plot displaying the past trajectory of effective population size

We found significant evidence for population expansion using Tajima's *D* and Fu's *F* neutrality statistics consistent with a previous hypothesis of a large, panmictic refugium in the center of the species' range (Kierepka & Latch, 2016; Long, 1972); however, no significant association was detected for the differentiated units (SAMOVA Group 1: BC‐Cariboo and BC‐Nicola) at the northwestern periphery in British Columbia (Table 3). The timing of the demographic expansion for the majority of the species' distribution appears to have occurred following the last glacial maximum, approximately 20,000 years ago, based on our Bayesian skyline plot analysis (Figure 6).

**TABLE 3:** Neutrality statistics for a fragment of the mtDNA D‐loop for the entire dataset and the two SAMOVA groupings at *K* = 2

Grouping	Tajima's *D*	*p*‐value	Fu's *Fs*	*p*‐value
Entire Dataset	−1.3139	.05	**−24.3101**	.002
SAMOVA Group1 (BC‐Cariboo and BC‐Nicola)	−0.4483	.27	−0.01937	.25
SAMOVA Group2	−1.3389	.05	−**24.2456**	.002

Note

Significance was assessed using 1,000 random permutations. Values in bold are significant at *α* = .05.

## 4 DISCUSSION

The American badger has had a long history in North America, with the earliest fossil specimens dating back to the early Pliocene, ~3.5 million years ago (Long, 1972). American badgers are thought to have survived the Pleistocene ice ages by residing in a single refugium, south of glacial extents, from which they recolonized northern latitudes following glacial retreat (Kierepka & Latch, 2016). By expanding on previous studies with newly acquired genetic data, we shed light on more complex patterns across the species' range.

Overall, higher levels of genetic diversity were observed for central sampling units, such as Colorado, Nebraska, and Kansas, compared to units at the range periphery, such as British Columbia, Ontario, and Ohio. Moreover, haplotype diversity generally decreased with increasing peripherality. In addition, the largely unresolved tree and star‐like haplotype network both displayed little discrete geographic structuring, indicative of a largely panmictic glacial refugium and recent population expansion (Figure 4) (Slatkin & Hudson, 1991). Furthermore, coalescent‐based analyses suggested a population expansion following the last glacial maximum. These patterns are consistent with another highly mobile generalist carnivore, the coyote (*Canis latrans*), which also lacks distinct phylogeographic structure and faced population size changes coinciding with historic changes in climate (Koblmüller et al., 2016).

On a more regional level, we expected to see shared or connected haplotypes from ancient populations in eastern Beringia with those from British Columbia, Canada, based on previous studies of North American postglacial colonization in other species (Shafer et al., 2010). Contrary to these expectations, we discovered that haplotypes recovered from Alaska and the Yukon were identical to haplotypes found in the Prairie provinces, the American Midwest, and the American West. Other species' populations have displayed this connectivity (Heintzman et al., 2016), owing to a hypothesized ice‐free corridor between the Cordilleran and Laurentide ice sheets through which animals migrated during the last glacial maximum, just east of present‐day British Columbia (Stalker, 1977). Recent evidence, however, suggests that the Cordilleran and Laurentide glaciers coalesced between 23,000 and 13,000 years ago, completely blocking any migration during this time period (Heintzman et al., 2016). Therefore, the shared haplotypes between ancient and contemporary populations suggest one of three possibilities: (1) badgers migrated to eastern Beringia from the grasslands of central North America when the ice‐free corridor reopened 13,000 years ago; (2) badgers from central North America migrated north more than 23,000 years ago prior to the closing of the ice‐free corridor; or (3) badgers from eastern Beringia colonized central North America after the corridor reopened 13,000 years ago, with badgers being extirpated from eastern Beringia following each scenario. Unfortunately, we did not have radiocarbon dates for the ancient specimens from which full sequences were recovered, but they are suggested to be late Wisconsin in age, with radiocarbon dates for other species ranging between 12,600 and 59,000 ybp at the Alaska site (Anderson, 1977), and between 20,900 and 39,000 ybp at the Yukon site (Fox‐Dobbs, Leonard, & Koch, 2008). Furthermore, radiocarbon dating for unsampled Yukon badger specimens date to 37,930 and 15,190 years ago (Harington & Clulow, 1973). These lines of evidence suggest that scenarios 2 or 3 are more plausible, as badgers were present in eastern Beringia before the corridor reopened. In terms of teasing apart the direction of migration, mitochondrial DNA‐based evidence in other studies suggests both directions are plausible. Bison exhibit no evidence of northward expansion into Alaska and the Yukon during the Holocene (Heintzman et al., 2016), whereas gray wolves show complete replacement of northern populations by those south of the glacial extent, after the glacial period (Koblmüller et al., 2016; Leonard et al., 2007). Although we cannot unequivocally infer directionality in American badgers, the moderate to high levels of genetic variation, alongside the low frequency of haplotypes with indels in the Midwest (Kierepka & Latch, 2016; also present in the Yukon) suggest a potential recolonization of American badgers into central North America after the ice‐free corridor reopened (scenario 3). Additional analyses, incorporating genetic information from Pleistocene fossil specimens south of glacial extents and in the region of the proposed ice‐free corridor, with explicit radiocarbon dates, are needed to further test this hypothesis.

Interestingly, we also discovered that peripheral populations in northwestern British Columbia have the highest level of differentiation from all other sampling units in North America (Table 2). These results suggest that populations in British Columbia either had a long history of isolation relative to all other sampled areas, or the potential existence of a cryptic glacial refugium within British Columbia. Similar genetic patterns showing a discrete genetic break in the southern interior of British Columbia have been observed for many other species (Gayathri Samarasekera et al., 2012; Jensen, Govindarajulu, & Russello, 2014; Parks, Wallin, Cushman, & McRae, 2015; Schmidt, Govindarajulu, Larsen, & Russello, 2020; Warren, Wallin, Beausoleil, & Warheit, 2016). The cause of this break, however, was attributed to various influences, including biological or climatic factors, isolation by distance, anthropogenic landscape features, or unsuitable habitat. Alternatively, this genetic pattern, observed across multiple species with different ecologies, may reflect a common glacial refugium within ice sheets. Mounting evidence of complex biogeographic patterns in British Columbia suggests that cryptic refugia may have existed within the Cordilleran ice sheet (Shafer et al., 2010). Yet, most species suggested to exist in such cryptic refugia have been either alpine or arctic, indicating a predisposition for tolerating cold climates. Badgers are typically restricted to dry grassland–shrubland ecosystems within North America, but their ability to tolerate unsuitable environmental conditions (Committee on the Status of Endangered Wildlife in Canada, 2012), alongside their semifossorial lifestyle, may have permitted badgers to live in refugia within ice sheets. Badgers in the BC‐Cariboo show particular hardiness for these environmental conditions, inhabiting atypical alpine and wetland habitats (Committee on the Status of Endangered Wildlife in Canada, 2012) in a region with harsh winter conditions (Symes, 2013). Additional studies involving denser geographic sampling and nuclear genome‐wide data for multiple species with overlapping distributions in the area are required to further test this hypothesis.

Overall, the expanded reconstruction of phylogeographic history of American badgers in North America offers a broader understanding of contemporary range‐wide patterns and provides insights for conservation management. The lack of geographic structuring, evidence of population expansion, and distinct peripheral populations with low genetic variation largely suggest that badgers resided in a single central refugium. Our results further indicate that patterns of mtDNA haplotype diversity do not coincide with current subspecies designations (Long, 1972), which has also been previously suggested (Kierepka & Latch, 2016). Spatial differentiation appears to be strongest between central and peripheral populations in western British Columbia, perhaps due to long‐term isolation. The preservation of this distinct genetic variation should be a priority. Currently, badger populations within the Canadian Prairie provinces (subspecies *T. t. taxus*) are listed as “Special Concern” under the Species at Risk Act (Government of Canada, 2011). The potential gene flow from ancient populations in eastern Beringia into populations in the Prairie provinces suggests that ongoing population monitoring should continue in this region if the unique genetic variation is to be preserved. Furthermore, the distinct genetic variation within the central plateau of British Columbia further supports a heightened conservation status for badgers in western British Columbia. Future work should further investigate these patterns with nuclear genome‐wide data collected across a denser sampling distribution around the geographic regions of most drastic genetic change.

## CONFLICT OF INTEREST

None declared.

## AUTHOR CONTRIBUTIONS

**Brett M. Ford:** Data curation (lead); formal analysis (lead); writing – original draft (lead); writing – review and editing (equal). **Anna Cornellas:** Data curation (supporting); formal analysis (supporting). **Jennifer A. Leonard:** Data curation (supporting); formal analysis (supporting); funding acquisition (supporting); resources (supporting); writing – review and editing (supporting). **Richard D. Weir:** Data curation (supporting); funding acquisition (supporting); resources (supporting); visualization (supporting); writing – review and editing (supporting). **Michael A. Russello:** Conceptualization (lead); formal analysis (supporting); funding acquisition (lead); project administration (lead); resources (supporting); supervision (lead); writing – original draft (supporting); writing – review and editing (equal).

## Supporting information

Appendix S1

Table S1

## Data Availability

All new mitochondrial DNA sequences generated have been deposited in GenBank (accession numbers MT632694–MT632752).
