# Supplementary material for: Spatiotemporal analyses suggest the role of glacial history and the ice‐free corridor in shaping American badger population genetic variation
Source: Ecol Evol. 2020 Jul 9;10(15):8345–57. doi: 10.1002/ece3.6541 (PMC7417222; doi:10.1002/ece3.6541)
Supplement: Supplementary file 1 — Appendix S1 [file ECE3-10-8345-s002.docx]

**Appendix S1**

Catalog number, museum codes, locality, and year of collection for recent specimens or age for ancient specimens (LP; late Pleistocene). Museums that kindly allowed sampling for this project include: Royal British Columbia Museum, Victoria, British Columbia (RBCM); Burke Museum of Natural History & Culture, Seattle, Washington (BMNHC); Beaty Biodiversity Museum, Vancouver, British Columbia (BBM); Charles R. Conner Museum, Pullman, Washington (CRCM); James R. Slater Museum of Natural History, Tacoma, Washington (PSM); Phillip L. Wright Zoological Museum, Missoula, Montana (PLWZM); American Museum of Natural History, New York, New York (AMNH); Canadian Museum of Nature, Ottawa, Canada (CMN).

| Catalog # | Museum | Location | State/Province | Year/ age |
| --- | --- | --- | --- | --- |
| A.C.C 552 | AMNH | Cripple Creek | Alaska | Late Pleistocene |
| F:AM 117098 | AMNH | Goldhill | Alaska | Late Pleistocene |
| F:AM 30786 | AMNH | Fairbanks | Alaska | Late Pleistocene |
| F:AM 30789 | AMNH | Cleary | Alaska | Late Pleistocene |
| F:AM 30826 | AMNH | Cripple Creek | Alaska | Late Pleistocene |
| F:AM 30829 | AMNH | Ester Creek | Alaska | Late Pleistocene |
| F:AM 30831 | AMNH | Cripple Creek | Alaska | Late Pleistocene |
| F:AM 30836 | AMNH | Cripple Creek | Alaska | Late Pleistocene |
| F:AM 30837-A | AMNH | Ester Creek | Alaska | Late Pleistocene |
| F:AM 30839 | AMNH | Fairbanks Creek | Alaska | Late Pleistocene |
| F:AM 30840 | AMNH | Cripple Creek | Alaska | Late Pleistocene |
| CMN 13486 | CMN | Gold Run Creek | Yukon | Late Pleistocene |
| CMN 35319 | CMN | Dawson Creek | Yukon | Late Pleistocene |
| 198118 | BMNHC | Madison County | Montana | 1981 |
| 34281 | BMNHC | Adams County | Washington | 1982 |
| 34282 | BMNHC | Franklin County | Washington | 1984 |
| 41395 | BMNHC | Walla Walla County | Washington | NA |
| 41396 | BMNHC | Canyon County | Idaho | 1951 |
| 81782 | BMNHC | Okanogan County | Washington | 2009 |
| 82298 | BMNHC | Grant County | Washington | 2012 |
| 03-3404 | CRCM | Moscow | Idaho | 1949 |
| 03-3405 | CRCM | Latah | Idaho | 1967 |
| 03-3406 | CRCM | Ferry | Idaho | 1954 |
| 03-3408 | CRCM | Elmore | Idaho | 1949 |
| 03-3409 | CRCM | Mt. View Guard Station | Idaho | 1950 |
| 13-222 | CRCM | Garfield County | Washington | 2005 |
| 47-38 | CRCM | Rosalia | Washington | 1947 |
| 48-420 | CRCM | Wawawai | Washington | 1948 |
| 51-321 | CRCM | Klickitat | Washington | 1951 |
| 52-125 | CRCM | Bonneville | Idaho | 1952 |
| 54-282 | CRCM | Pullman | Washington | 1954 |
| 57-245 | CRCM | Latah | Idaho | 1957 |
| 61-221 | CRCM | Wawawai | Washington | 1960 |
| 78-501 | CRCM | Pullman | Washington | 1978 |
| 82-144 | CRCM | Nespelem | Washington | 1981 |
| 84-1 | CRCM | Central Ferry | Washington | 1977 |
| 84-2 | CRCM | Ewan | Washington | 1982 |
| 1242 | PLWZM | Browns Lake | Montana | 1949 |
| 12532 | PLWZM | Miller Creek | Montana | 1967 |
| 1421 | PLWZM | Florence | Montana | 1950 |
| 1422 | PLWZM | Stony Cr | Montana | 1950 |
| 3411 | PLWZM | Norris | Montana | 1950 |
| 3416 | PLWZM | Gallatin County | Montana | 1950 |
| 3874 | PLWZM | Willow Cr Camp | Montana | 1952 |
| 6477 | PLWZM | Krumholz | Montana | 1961 |
| 7027 | PLWZM | Nine Mile Creek | Montana | 1961 |
| 7028 | PLWZM | Ft Benton | Montana | 1961 |
| 758 | PLWZM | S. Yellow Bay | Montana | 1948 |
| 8877 | PLWZM | Bitterroot Valley | Montana | 1964 |
| 9929 | PLWZM | Townsend | Montana | 1965 |
| Mammal-01358 | PSM | Yakima | Washington | 1948 |
| Mammal-03485 | PSM | Sema Meadows | Washington | 1952 |
| Mammal-04007 | PSM | Pack River | Idaho | 1953 |
| Mammal-04318 | PSM | Douglas | Washington | 1953 |
| Mammal-06337 | PSM | Garfield | Washington | 1953 |
| Mammal-09252 | PSM | Douglas | Washington | 1962 |
| 015658Z | RBCM | Cranbrook | British Columbia | 1985 |
| 10230 | RBCM | Fernie | British Columbia | NA |
| 11403 | RBCM | Vernon | British Columbia | 1982 |
| 11438 | RBCM | Vernon | British Columbia | 1983 |
| 11839 | RBCM | Pend d’Oreille River | British Columbia | 1983 |
| 12349 | RBCM | Savona | British Columbia | 1981 |
| 13720 | RBCM | Trail | British Columbia | 1984 |
| 13721 | RBCM | Trail | British Columbia | 1984 |
| 14862 | RBCM | Anarchist Mt | British Columbia | 1984 |
| 15659 | RBCM | Fernie | British Columbia | 1985 |
| 15675 | RBCM | Lumby | British Columbia | 1985 |
| 15679 | RBCM | Anarchist Mt | British Columbia | 1985 |
| 16289 | RBCM | Lumby | British Columbia | 1987 |
| 16741 | RBCM | Kamloops | British Columbia | 1988 |
| 16742 | RBCM | Kamloops | British Columbia | 1988 |
| 16744 | RBCM | Kamloops | British Columbia | 1988 |
| 16756 | RBCM | Trail | British Columbia | 1988 |
| 16757 | RBCM | Trail | British Columbia | 1988 |
| 16758 | RBCM | Princeton | British Columbia | 1988 |
| 1736 | RBCM | Anarchist Mt | British Columbia | 1936 |
| 6833 | RBCM | Williams Lake | British Columbia | 1960 |
| 9076 | RBCM | Manning Province Park | British Columbia | 1974 |
| 9875 | RBCM | Pinewoods | British Columbia | 1978 |
| M000001 | BBM | Kamloops | British Columbia | 1934 |
| M000540 | BBM | Anarchist Mt | British Columbia | 1931 |
| M001499 | BBM | Invermere | British Columbia | 1945 |
| M004122 | BBM | Penticton | British Columbia | 1952 |
| M006010 | BBM | Kamloops | British Columbia | 1928 |
| M007490 | BBM | Invermere | British Columbia | 1959 |
| M009080 | BBM | Grindrod | British Columbia | 1947 |
| M016813 | BBM | Armstrong | British Columbia | 1989 |
