# Supplementary material for: Spatiotemporal analyses suggest the role of glacial history and the ice‐free corridor in shaping American badger population genetic variation
Source: Ecol Evol. 2020 Jul 9;10(15):8345–57. doi: 10.1002/ece3.6541 (PMC7417222; doi:10.1002/ece3.6541)
Supplement: Supplementary file 2 — Table S1 [file ECE3-10-8345-s001.docx]

Table S1. Sample information for all American badgers (n=1207) for which mtDNA haplotype data were obtained.

| Sample | Collection | Catalog Number | Subspecies | Haplotype | Latitude | Longitude | Category | Source | Genbank |
| --- | --- | --- | --- | --- | --- | --- | --- | --- | --- |
| JAL146 | AMNH | F:AM 30786 | NA | 48 | 64.831365 | -147.68755 | New ancient | This study | MT632737 |
| PERM607 | CMN | 13486 | NA | 97 | 63.694878 | -138.60463 | New ancient | This study | MT632747 |
| CA15_7416 | BC MOECC | CA15_7416 | *jeffersonii* | 116 | 39.8847701 | -122.54503 | New contemporary | This study | MT632750 |
| CA15_7417 | BC MOECC | CA15_7417 | *jeffersonii* | 112 | 39.9291773 | -122.26957 | New contemporary | This study | MT632749 |
| CO15_7353 | BC MOECC | CO15_7353 | *jeffersonii* | 47 | 39.0512964 | -108.55161 | New contemporary | This study | MT632733 |
| CO15_7354 | BC MOECC | CO15_7354 | *jeffersonii* | 34 | 40.578736 | -105.10634 | New contemporary | This study | MT632727 |
| NM15_7364 | BC MOECC | NM15_7364 | *berlandieri* | 12 | 32.5784743 | -104.41904 | New contemporary | This study | MT632720 |
| OR15_7350 | BC MOECC | OR15_7350 | *jeffersonii* | 53 | 42.1739835 | -121.75652 | New contemporary | This study | MT632738 |
| SK15_7349 | BC MOECC | SK15_7349 | *taxus* | 2 | 52.033444 | -106.67044 | New contemporary | This study | MT632694 |
| UT15_7365 | BC MOECC | UT15_7365 | *jeffersonii* | 91 | 41.0350558 | -111.67496 | New contemporary | This study | MT632745 |
| UT15_7366 | BC MOECC | UT15_7366 | *jeffersonii* | 93 | 41.0389564 | -111.93317 | New contemporary | This study | MT632746 |
| WI15_7367 | BC MOECC | WI15_7367 | *taxus* | 12 | 45.7179094 | -91.799366 | New contemporary | This study | MT632721 |
| WI15_7368 | BC MOECC | WI15_7368 | *taxus* | 12 | 45.751976 | -91.758856 | New contemporary | This study | MT632722 |
| WI15_7369 | BC MOECC | WI15_7369 | *taxus* | 26 | 45.1929826 | -91.827782 | New contemporary | This study | MT632724 |
| CR6833 | RBCM | 6833 | *jeffersonii* | 4 | 52.1294 | -122.1383 | New historical | This study | MT632711 |
| EK007490 | UBCBBM | M007490 | *jeffersonii* | 44 | 50.508619 | -116.03133 | New historical | This study | MT632729 |
| EK1499 | UBCBBM | M001499 | *jeffersonii* | 2 | 50.481187 | -116.0369 | New historical | This study | MT632695 |
| EK15658 | RBCM | 15658 | *jeffersonii* | 2 | 49.513 | -115.7694 | New historical | This study | MT632696 |
| EK15659 | RBCM | 15659 | *jeffersonii* | 44 | 49.481785 | -115.07211 | New historical | This study | MT632730 |
| ID03_3405 | CRCM | 03-3405 | *jeffersonii* | 58 | 46.746358 | -116.97877 | New historical | This study | MT632739 |
| ID03_3406 | CRCM | 03-3406 | *jeffersonii* | 47 | 44.7604369 | -116.90433 | New historical | This study | MT632734 |
| ID03_3408 | CRCM | 03-3408 | *jeffersonii* | 118 | 43.34 | -115.47 | New historical | This study | MT632752 |
| ID57_245 | CRCM | 57-245 | *jeffersonii* | 2 | 46.943 | -116.6499 | New historical | This study | MT632697 |
| IDMamm04007 | PSM | Mamm04007 | *jeffersonii* | 2 | 48.4635 | -116.5412 | New historical | This study | MT632698 |
| MT1242 | PLWZM | 1242 | *jeffersonii* | 105 | 46.960126 | -113.00092 | New historical | This study | MT632748 |
| MT12532 | PLWZM | 12532 | *jeffersonii* | 2 | 46.807244 | -114.07204 | New historical | This study | MT632699 |
| MT1421 | PLWZM | 1421 | *jeffersonii* | 31 | 46.867282 | -113.94503 | New historical | This study | MT632725 |
| MT198118 | BMNHC | 198118 | *jeffersonii* | 2 | 45.3367 | -111.74 | New historical | This study | MT632700 |
| MT3874 | PLWZM | 3874 | *jeffersonii* | 12 | 47.454651 | -114.15616 | New historical | This study | MT632723 |
| MT6477 | PLWZM | 6477 | *jeffersonii* | 39 | 45.169894 | -109.25033 | New historical | This study | MT632728 |
| MT758 | PLWZM | 758 | *jeffersonii* | 44 | 47.72706 | -114.03809 | New historical | This study | MT632731 |
| MT8877 | PLWZM | T8877 | *jeffersonii* | 2 | 46.457194 | -114.07308 | New historical | This study | MT632701 |
| NI9076 | RBCM | 9076 | *jeffersonii* | 2 | 49.0667 | -120.7833 | New historical | This study | MT632702 |
| NI9875 | RBCM | 9875 | *jeffersonii* | 2 | 49.05 | -120.7667 | New historical | This study | MT632703 |
| OK11403 | RBCM | 11403 | *jeffersonii* | 58 | 50.268666 | -119.29068 | New historical | This study | MT632740 |
| OK11438 | RBCM | 11438 | *jeffersonii* | 4 | 50.246448 | -119.2333 | New historical | This study | MT632712 |
| OK13720 | RBCM | 13720 | *jeffersonii* | 2 | 49.103399 | -117.71493 | New historical | This study | MT632704 |
| OK13721 | RBCM | 13721 | *jeffersonii* | 58 | 49.090588 | -117.72462 | New historical | This study | MT632741 |
| OK14862 | RBCM | 14862 | *jeffersonii* | 2 | 49.043362 | -119.34677 | New historical | This study | MT632705 |
| OK15675 | RBCM | 15675 | *jeffersonii* | 2 | 50.264621 | -118.9629 | New historical | This study | MT632706 |
| OK15679 | RBCM | 15679 | *jeffersonii* | 4 | 49.032676 | -119.32402 | New historical | This study | MT632713 |
| OK16289 | RBCM | 16289 | *jeffersonii* | 2 | 50.238301 | -118.96988 | New historical | This study | MT632707 |
| OK16756 | RBCM | 16756 | *jeffersonii* | 4 | 49.101147 | -117.67075 | New historical | This study | MT632714 |
| OK16757 | RBCM | 16757 | *jeffersonii* | 58 | 49.105888 | -117.70518 | New historical | This study | MT632742 |
| OK16758 | RBCM | 16758 | *jeffersonii* | 58 | 49.4911 | -119.5886 | New historical | This study | MT632743 |
| OK1736 | RBCM | 1736 | *jeffersonii* | 2 | 49.0386 | -119.3361 | New historical | This study | MT632708 |
| OK4122 | UBCBBM | M004122 | *jeffersonii* | 2 | 49.49882 | -119.59378 | New historical | This study | MT632709 |
| OK540 | UBCBBM | M00540 | *jeffersonii* | 4 | 49.021465 | -119.33775 | New historical | This study | MT632715 |
| OK9080 | UBCBBM | M009080 | *jeffersonii* | 2 | 50.628094 | -119.12345 | New historical | This study | MT632710 |
| TH000001 | UBCBBM | M000001 | *jeffersonii* | 117 | 50.661985 | -120.3533 | New historical | This study | MT632751 |
| TH006010 | UBCBBM | M006010 | *jeffersonii* | 4 | 50.674522 | -120.32727 | New historical | This study | MT632716 |
| TH12349 | RBCM | 12349 | *jeffersonii* | 4 | 50.75 | -120.85 | New historical | This study | MT632717 |
| TH16744 | RBCM | 16744 | *jeffersonii* | 44 | 50.683978 | -120.31028 | New historical | This study | MT632732 |
| WA51_321 | CRCM | 51-321 | *jeffersonii* | 33 | 45.8170668 | -121.15257 | New historical | This study | MT632726 |
| WA54_282 | CRCM | 54-282 | *jeffersonii* | 4 | 46.73686 | -117.19855 | New historical | This study | MT632718 |
| WA78_501 | CRCM | 78-501 | *jeffersonii* | 47 | 46.718178 | -117.15152 | New historical | This study | MT632735 |
| WA842 | CRCM | 84-2 | *jeffersonii* | 47 | 47.1168 | -117.7349 | New historical | This study | MT632736 |
| WAMamm03485 | PSM | Mamm03485 | *jeffersonii* | 58 | 48.6821418 | -117.06939 | New historical | This study | MT632744 |
| WAMamm09252 | PSM | Mamm09252 | *jeffersonii* | 4 | 47.64028 | -120.21639 | New historical | This study | MT632719 |
| AB206 | - | - | *taxus* | 2 | 51.8171771 | -112.8544 | Previously published | Kierepka & Latch (2016) | KU763538.1 |
| AB216 | - | - | *taxus* | 2 | 51.8171771 | -112.8544 | Previously published | Kierepka & Latch (2016) | KU763539.1 |
| AB249 | - | - | *taxus* | 24 | 51.8171771 | -112.8544 | Previously published | Kierepka & Latch (2016) | KU763540.1 |
| AB51 | - | - | *taxus* | 25 | 51.8171771 | -112.8544 | Previously published | Kierepka & Latch (2016) | KU763541.1 |
| AB53 | - | - | *taxus* | 44 | 51.8171771 | -112.8544 | Previously published | Kierepka & Latch (2016) | KU763542.1 |
| AL_4DGY320 | - | - | *taxus* | 2 | 50.261534 | -113.92747 | Previously published | Ethier et al. (2012) | GU901531.1 |
| AL_4DHU114 | - | - | *taxus* | 2 | 50.261534 | -113.92747 | Previously published | Ethier et al. (2012) | GU901530.1 |
| AL_4DHU115 | - | - | *taxus* | 2 | 50.261534 | -113.92747 | Previously published | Ethier et al. (2012) | GU901529.1 |
| AL_4DHU328 | - | - | *taxus* | 2 | 50.261534 | -113.92747 | Previously published | Ethier et al. (2012) | GU901524.1 |
| AL_4ITX683 | - | - | *taxus* | 103 | 50.261534 | -113.92747 | Previously published | Ethier et al. (2012) | GU901528.1 |
| AL_4ITX684 | - | - | *taxus* | 2 | 50.261534 | -113.92747 | Previously published | Ethier et al. (2012) | GU901527.1 |
| AL_4ITX685 | - | - | *taxus* | 73 | 50.261534 | -113.92747 | Previously published | Ethier et al. (2012) | GU901526.1 |
| AL_4ITX686 | - | - | *taxus* | 73 | 50.261534 | -113.92747 | Previously published | Ethier et al. (2012) | GU901525.1 |
| AL_4ITZ770 | - | - | *taxus* | 2 | 50.261534 | -113.92747 | Previously published | Ethier et al. (2012) | GU901523.1 |
| AL_AB_BAIRU069 | - | - | *taxus* | 2 | 50.261534 | -113.92747 | Previously published | Ethier et al. (2012) | GU901485.1 |
| AL_AB_BAIRU172 | - | - | *taxus* | 2 | 50.261534 | -113.92747 | Previously published | Ethier et al. (2012) | GU901484.1 |
| AL_ABBA_CKBA | - | - | *taxus* | 52 | 50.261534 | -113.92747 | Previously published | Ethier et al. (2012) | GU901492.1 |
| AL_ABBA_IRB679 | - | - | *taxus* | 2 | 50.261534 | -113.92747 | Previously published | Ethier et al. (2012) | GU901498.1 |
| AL_ABBA_IRISB116 | - | - | *taxus* | 2 | 50.261534 | -113.92747 | Previously published | Ethier et al. (2012) | GU901497.1 |
| AL_ABBA_IRT120 | - | - | *taxus* | 2 | 50.261534 | -113.92747 | Previously published | Ethier et al. (2012) | GU901496.1 |
| AL_ABBA_IRT122 | - | - | *taxus* | 12 | 50.261534 | -113.92747 | Previously published | Ethier et al. (2012) | GU901489.1 |
| AL_ABBA_IRT123 | - | - | *taxus* | 2 | 50.261534 | -113.92747 | Previously published | Ethier et al. (2012) | GU901495.1 |
| AL_ABBA_IRU070 | - | - | *taxus* | 2 | 50.261534 | -113.92747 | Previously published | Ethier et al. (2012) | GU901493.1 |
| AL_ABBA_IRU088 | - | - | *taxus* | 2 | 50.261534 | -113.92747 | Previously published | Ethier et al. (2012) | GU901494.1 |
| AL_ABBA_IRU168 | - | - | *taxus* | 12 | 50.261534 | -113.92747 | Previously published | Ethier et al. (2012) | GU901490.1 |
| AL_ABBA_IRU677 | - | - | *taxus* | 2 | 50.261534 | -113.92747 | Previously published | Ethier et al. (2012) | GU901499.1 |
| AL_ABBA_IRU678 | - | - | *taxus* | 2 | 50.261534 | -113.92747 | Previously published | Ethier et al. (2012) | GU901500.1 |
| AL_ABBA_IRU681 | - | - | *taxus* | 50 | 50.261534 | -113.92747 | Previously published | Ethier et al. (2012) | GU901486.1 |
| AL_ABBA_IRU683 | - | - | *taxus* | 2 | 50.261534 | -113.92747 | Previously published | Ethier et al. (2012) | GU901501.1 |
| AL_ABBA_IRU684 | - | - | *taxus* | 52 | 50.261534 | -113.92747 | Previously published | Ethier et al. (2012) | GU901491.1 |
| AL_ABBA_IRZ308 | - | - | *taxus* | 36 | 50.261534 | -113.92747 | Previously published | Ethier et al. (2012) | GU901488.1 |
| AL_ABBA_ISA700 | - | - | *taxus* | 39 | 50.261534 | -113.92747 | Previously published | Ethier et al. (2012) | GU901487.1 |
| AL_ABBA12c | - | - | *taxus* | 2 | 50.261534 | -113.92747 | Previously published | Ethier et al. (2012) | GU901503.1 |
| AL_ABBA2a | - | - | *taxus* | 2 | 50.261534 | -113.92747 | Previously published | Ethier et al. (2012) | GU901502.1 |
| AL_ABBA71a | - | - | *taxus* | 2 | 50.261534 | -113.92747 | Previously published | Ethier et al. (2012) | GU901504.1 |
| AL_Badger15_AB | - | - | *taxus* | 2 | 50.261534 | -113.92747 | Previously published | Ethier et al. (2012) | GU901514.1 |
| AL_Badgers_13 | - | - | *taxus* | 2 | 50.261534 | -113.92747 | Previously published | Ethier et al. (2012) | GU901509.1 |
| AL_Badgers_16 | - | - | *taxus* | 36 | 50.261534 | -113.92747 | Previously published | Ethier et al. (2012) | GU901508.1 |
| AL_Badgers_17 | - | - | *taxus* | 50 | 50.261534 | -113.92747 | Previously published | Ethier et al. (2012) | GU901507.1 |
| AL_Badgers_3 | - | - | *taxus* | 2 | 50.261534 | -113.92747 | Previously published | Ethier et al. (2012) | GU901513.1 |
| AL_Badgers_4 | - | - | *taxus* | 105 | 50.261534 | -113.92747 | Previously published | Ethier et al. (2012) | GU901506.1 |
| AL_Badgers_5 | - | - | *taxus* | 2 | 50.261534 | -113.92747 | Previously published | Ethier et al. (2012) | GU901512.1 |
| AL_Badgers_7 | - | - | *taxus* | 50 | 50.261534 | -113.92747 | Previously published | Ethier et al. (2012) | GU901505.1 |
| AL_Badgers_8 | - | - | *taxus* | 52 | 50.261534 | -113.92747 | Previously published | Ethier et al. (2012) | GU901510.1 |
| AL_Badgers_9 | - | - | *taxus* | 2 | 50.261534 | -113.92747 | Previously published | Ethier et al. (2012) | GU901511.1 |
| AL_ILC796_2_ | - | - | *taxus* | 109 | 50.261534 | -113.92747 | Previously published | Ethier et al. (2012) | GU901532.1 |
| AL15_7342 | - | - | *taxus* | 2 | 51.110772 | -114.00423 | Previously published | Ford et al. (2019) | MK882700.1 |
| AL15_7343 | - | - | *taxus* | 44 | 51.1249402 | -114.01692 | Previously published | Ford et al. (2019) | MK882701.1 |
| AL15_7344 | - | - | *taxus* | 2 | 51.1197727 | -114.01252 | Previously published | Ford et al. (2019) | MK882702.1 |
| AL15_7345 | - | - | *taxus* | 113 | 52.4179222 | -113.73252 | Previously published | Ford et al. (2019) | MK882703.1 |
| AL15_7348 | - | - | *taxus* | 31 | 49.7814806 | -114.15838 | Previously published | Ford et al. (2019) | MK882704.1 |
| AZ001 | - | - | *berlandieri* | 13 | 32.405669 | -110.07132 | Previously published | Kierepka & Latch (2016) | KU764092.1 |
| AZ002 | - | - | *berlandieri* | 1 | 32.405669 | -110.07132 | Previously published | Kierepka & Latch (2016) | KU764093.1 |
| CA_001 | - | - | *berlandieri* | 58 | 40.565597 | -121.95079 | Previously published | Kierepka & Latch (2016) | KU764145.1 |
| CO001 | - | - | *jeffersonii* | 95 | 38.9954611 | -105.54788 | Previously published | Kierepka & Latch (2016) | Direct from authors |
| CO251 | - | - | *jeffersonii* | 80 | 38.9954611 | -105.54788 | Previously published | Kierepka & Latch (2016) | KU763617.1 |
| CO270 | - | - | *jeffersonii* | 78 | 38.9954611 | -105.54788 | Previously published | Kierepka & Latch (2016) | KU763618.1 |
| CO282 | - | - | *jeffersonii* | 1 | 38.9954611 | -105.54788 | Previously published | Kierepka & Latch (2016) | KU763619.1 |
| CR08_1054 | - | - | *jeffersonii* | 4 | 52.010321 | -121.86719 | Previously published | Ford et al. (2019) | MK882705.1 |
| CR08_1056 | - | - | *jeffersonii* | 4 | 51.643586 | -121.2973 | Previously published | Ford et al. (2019) | MK882706.1 |
| CR08_1057 | - | - | *jeffersonii* | 4 | 51.647849 | -121.29798 | Previously published | Ford et al. (2019) | MK882707.1 |
| CR09_2392 | - | - | *jeffersonii* | 4 | 51.64508 | -121.29799 | Previously published | Ford et al. (2019) | MK882708.1 |
| CR09_2394 | - | - | *jeffersonii* | 4 | 52.0210987 | -121.88092 | Previously published | Ford et al. (2019) | MK882709.1 |
| CR09_2395 | - | - | *jeffersonii* | 4 | 52.021298 | -121.88118 | Previously published | Ford et al. (2019) | MK882710.1 |
| CR09_2397 | - | - | *jeffersonii* | 4 | 52.0299856 | -121.88604 | Previously published | Ford et al. (2019) | MK882711.1 |
| CR09_2398 | - | - | *jeffersonii* | 4 | 51.7987201 | -121.42356 | Previously published | Ford et al. (2019) | MK882712.1 |
| CR10_2574 | - | - | *jeffersonii* | 4 | 52.0700406 | -121.9197 | Previously published | Ford et al. (2019) | MK882713.1 |
| CR10_2575 | - | - | *jeffersonii* | 4 | 52.0354541 | -121.89962 | Previously published | Ford et al. (2019) | MK882714.1 |
| CR10_2579 | - | - | *jeffersonii* | 4 | 51.661019 | -121.28777 | Previously published | Ford et al. (2019) | MK882715.1 |
| CR11_2977 | - | - | *jeffersonii* | 4 | 51.7734897 | -121.39114 | Previously published | Ford et al. (2019) | MK882716.1 |
| CR13_4211 | - | - | *jeffersonii* | 4 | 51.6544018 | -121.32716 | Previously published | Ford et al. (2019) | MK882717.1 |
| CR13_4212 | - | - | *jeffersonii* | 4 | 51.6485542 | -121.29634 | Previously published | Ford et al. (2019) | MK882718.1 |
| CR13_4213 | - | - | *jeffersonii* | 4 | 51.8074482 | -121.44493 | Previously published | Ford et al. (2019) | MK882719.1 |
| CR13_4214 | - | - | *jeffersonii* | 4 | 52.0956514 | -121.92504 | Previously published | Ford et al. (2019) | MK882720.1 |
| CR13_4215 | - | - | *jeffersonii* | 4 | 52.0944031 | -121.92323 | Previously published | Ford et al. (2019) | MK882721.1 |
| CR15_6292 | - | - | *jeffersonii* | 4 | 51.50788 | -121.92249 | Previously published | Ford et al. (2019) | MK882722.1 |
| CR15_6294 | - | - | *jeffersonii* | 4 | 51.5760681 | -121.13561 | Previously published | Ford et al. (2019) | MK882723.1 |
| CR15_6298 | - | - | *jeffersonii* | 4 | 51.7514008 | -121.34679 | Previously published | Ford et al. (2019) | MK882724.1 |
| CR15_6772 | - | - | *jeffersonii* | 4 | 51.6830436 | -121.30965 | Previously published | Ford et al. (2019) | MK882725.1 |
| CR15_6775 | - | - | *jeffersonii* | 4 | 52.1184099 | -122.02343 | Previously published | Ford et al. (2019) | MK882726.1 |
| CR15_6776 | - | - | *jeffersonii* | 4 | 52.0982143 | -121.92805 | Previously published | Ford et al. (2019) | MK882727.1 |
| CR15_6777 | - | - | *jeffersonii* | 4 | 51.6508614 | -121.28927 | Previously published | Ford et al. (2019) | MK882728.1 |
| CR15_7347 | - | - | *jeffersonii* | 4 | 51.6555145 | -121.29264 | Previously published | Ford et al. (2019) | MK882729.1 |
| EK10230 | - | - | *jeffersonii* | 2 | 49.534751 | -115.04009 | Previously published | Ford et al. (2019) | MK882730.1 |
| EK12_3449 | - | - | *jeffersonii* | 44 | 50.001307 | -115.7581 | Previously published | Ford et al. (2019) | MK882731.1 |
| EK12_3555 | - | - | *jeffersonii* | 2 | 49.782394 | -115.7387 | Previously published | Ford et al. (2019) | MK882732.1 |
| EK12_3556 | - | - | *jeffersonii* | 2 | 49.629073 | -115.93235 | Previously published | Ford et al. (2019) | MK882733.1 |
| EK12_3557 | - | - | *jeffersonii* | 44 | 49.7823945 | -115.7387 | Previously published | Ford et al. (2019) | MK882734.1 |
| EK12_3558 | - | - | *jeffersonii* | 58 | 49.629073 | -115.93235 | Previously published | Ford et al. (2019) | MK882735.1 |
| EK12_3559 | - | - | *jeffersonii* | 44 | 49.7823945 | -115.7387 | Previously published | Ford et al. (2019) | MK882736.1 |
| EK12_3560 | - | - | *jeffersonii* | 44 | 49.3695861 | -115.23399 | Previously published | Ford et al. (2019) | MK882737.1 |
| EK12_3561 | - | - | *jeffersonii* | 44 | 49.8380695 | -115.73932 | Previously published | Ford et al. (2019) | MK882738.1 |
| EK12_3562 | - | - | *jeffersonii* | 2 | 49.6179944 | -115.62489 | Previously published | Ford et al. (2019) | MK882739.1 |
| EK12_3563 | - | - | *jeffersonii* | 12 | 49.893973 | -114.89294 | Previously published | Ford et al. (2019) | MK882740.1 |
| EK13_4355 | - | - | *jeffersonii* | 12 | 49.305351 | -114.99604 | Previously published | Ford et al. (2019) | MK882741.1 |
| EK14_4602 | - | - | *jeffersonii* | 44 | 46.899719 | -115.04853 | Previously published | Ford et al. (2019) | MK882742.1 |
| EK14_4603 | - | - | *jeffersonii* | 44 | 49.3053513 | -114.99604 | Previously published | Ford et al. (2019) | MK882743.1 |
| EK14_4604 | - | - | *jeffersonii* | 44 | 49.50641 | -115.06699 | Previously published | Ford et al. (2019) | MK882744.1 |
| EK14_4606 | - | - | *jeffersonii* | 44 | 49.5114378 | -115.59568 | Previously published | Ford et al. (2019) | MK882745.1 |
| EK14_4607 | - | - | *jeffersonii* | 58 | 49.5064101 | -115.06699 | Previously published | Ford et al. (2019) | MK882746.1 |
| EK14_4608 | - | - | *jeffersonii* | 44 | 49.8380695 | -115.73932 | Previously published | Ford et al. (2019) | MK882747.1 |
| EK14_4610 | - | - | *jeffersonii* | 44 | 49.696714 | -115.6841 | Previously published | Ford et al. (2019) | MK882748.1 |
| EK14_4611 | - | - | *jeffersonii* | 2 | 49.4039631 | -115.37537 | Previously published | Ford et al. (2019) | MK882749.1 |
| EK14_4612 | - | - | *jeffersonii* | 44 | 49.449161 | -115.44304 | Previously published | Ford et al. (2019) | MK882750.1 |
| EK14_4613 | - | - | *jeffersonii* | 44 | 49.299895 | -114.90554 | Previously published | Ford et al. (2019) | MK882751.1 |
| EK14_4614 | - | - | *jeffersonii* | 12 | 50.5084222 | -116.09041 | Previously published | Ford et al. (2019) | MK882752.1 |
| EK15_6186 | - | - | *jeffersonii* | 44 | 49.529323 | -115.04091 | Previously published | Ford et al. (2019) | MK882753.1 |
| EK15_6187 | - | - | *jeffersonii* | 44 | 50.095998 | -115.53218 | Previously published | Ford et al. (2019) | MK882754.1 |
| EK15_6331 | - | - | *jeffersonii* | 2 | 49.5117544 | -114.65642 | Previously published | Ford et al. (2019) | MK882755.1 |
| EK15_6332 | - | - | *jeffersonii* | 44 | 49.587432 | -115.8103 | Previously published | Ford et al. (2019) | MK882756.1 |
| EK15_6782 | - | - | *jeffersonii* | 2 | 49.584475 | -114.96635 | Previously published | Ford et al. (2019) | MK882757.1 |
| EK15_7394 | - | - | *jeffersonii* | 44 | 49.611882 | -115.89389 | Previously published | Ford et al. (2019) | MK882758.1 |
| EK15_7414 | - | - | *jeffersonii* | 44 | 49.1411179 | -115.4628 | Previously published | Ford et al. (2019) | MK882759.1 |
| IA05 | - | - | *taxus* | 1 | 42.0751151 | -93.497193 | Previously published | Kierepka & Latch (2016) | KU763620.1 |
| IA07 | - | - | *taxus* | 1 | 42.0751151 | -93.497193 | Previously published | Kierepka & Latch (2016) | KU763621.1 |
| IA10 | - | - | *taxus* | 1 | 42.0751151 | -93.497193 | Previously published | Kierepka & Latch (2016) | KU763622.1 |
| IA118 | - | - | *taxus* | 82 | 42.0751151 | -93.497193 | Previously published | Kierepka & Latch (2016) | KU763623.1 |
| IA12 | - | - | *taxus* | 45 | 42.0751151 | -93.497193 | Previously published | Kierepka & Latch (2016) | KU763624.1 |
| IA14 | - | - | *taxus* | 46 | 42.0751151 | -93.497193 | Previously published | Kierepka & Latch (2016) | KU763625.1 |
| IA157 | - | - | *taxus* | 82 | 42.0751151 | -93.497193 | Previously published | Kierepka & Latch (2016) | KU763626.1 |
| IA163 | - | - | *taxus* | 1 | 42.0751151 | -93.497193 | Previously published | Kierepka & Latch (2016) | KU763627.1 |
| IA167 | - | - | *taxus* | 1 | 42.0751151 | -93.497193 | Previously published | Kierepka & Latch (2016) | KU763628.1 |
| IA168 | - | - | *taxus* | 2 | 42.0751151 | -93.497193 | Previously published | Kierepka & Latch (2016) | KU763629.1 |
| IA176 | - | - | *taxus* | 1 | 42.0751151 | -93.497193 | Previously published | Kierepka & Latch (2016) | KU763630.1 |
| IA177 | - | - | *taxus* | 2 | 42.0751151 | -93.497193 | Previously published | Kierepka & Latch (2016) | KU763631.1 |
| IA189 | - | - | *taxus* | 71 | 42.0751151 | -93.497193 | Previously published | Kierepka & Latch (2016) | KU763632.1 |
| IA192_10 | - | - | *taxus* | 2 | 42.0751151 | -93.497193 | Previously published | Kierepka & Latch (2016) | KU763633.1 |
| IA192_12 | - | - | *taxus* | 1 | 42.0751151 | -93.497193 | Previously published | Kierepka & Latch (2016) | KU763634.1 |
| IA193 | - | - | *taxus* | 46 | 42.0751151 | -93.497193 | Previously published | Kierepka & Latch (2016) | KU763635.1 |
| IA194 | - | - | *taxus* | 1 | 42.0751151 | -93.497193 | Previously published | Kierepka & Latch (2016) | KU763636.1 |
| IA201 | - | - | *taxus* | 46 | 42.0751151 | -93.497193 | Previously published | Kierepka & Latch (2016) | KU763637.1 |
| IA203_10 | - | - | *taxus* | 46 | 42.0751151 | -93.497193 | Previously published | Kierepka & Latch (2016) | KU763638.1 |
| IA203_12 | - | - | *taxus* | 46 | 42.0751151 | -93.497193 | Previously published | Kierepka & Latch (2016) | KU763639.1 |
| IA204 | - | - | *taxus* | 26 | 42.0751151 | -93.497193 | Previously published | Kierepka & Latch (2016) | KU763640.1 |
| IA205 | - | - | *taxus* | 26 | 42.0751151 | -93.497193 | Previously published | Kierepka & Latch (2016) | KU763641.1 |
| IA206 | - | - | *taxus* | 82 | 42.0751151 | -93.497193 | Previously published | Kierepka & Latch (2016) | KU763642.1 |
| IA207 | - | - | *taxus* | 65 | 42.0751151 | -93.497193 | Previously published | Kierepka & Latch (2016) | KU763643.1 |
| IA208_10 | - | - | *taxus* | 2 | 42.0751151 | -93.497193 | Previously published | Kierepka & Latch (2016) | KU763644.1 |
| IA208_12 | - | - | *taxus* | 1 | 42.0751151 | -93.497193 | Previously published | Kierepka & Latch (2016) | KU763645.1 |
| IA209_10 | - | - | *taxus* | 82 | 42.0751151 | -93.497193 | Previously published | Kierepka & Latch (2016) | KU763646.1 |
| IA209_12 | - | - | *taxus* | 2 | 42.0751151 | -93.497193 | Previously published | Kierepka & Latch (2016) | KU763647.1 |
| IA21 | - | - | *taxus* | 8 | 42.0751151 | -93.497193 | Previously published | Kierepka & Latch (2016) | KU763648.1 |
| IA210 | - | - | *taxus* | 26 | 42.0751151 | -93.497193 | Previously published | Kierepka & Latch (2016) | KU763649.1 |
| IA217 | - | - | *taxus* | 82 | 42.0751151 | -93.497193 | Previously published | Kierepka & Latch (2016) | KU763650.1 |
| IA224 | - | - | *taxus* | 71 | 42.0751151 | -93.497193 | Previously published | Kierepka & Latch (2016) | KU763651.1 |
| IA226 | - | - | *taxus* | 46 | 42.0751151 | -93.497193 | Previously published | Kierepka & Latch (2016) | KU763652.1 |
| IA229 | - | - | *taxus* | 87 | 42.0751151 | -93.497193 | Previously published | Kierepka & Latch (2016) | KU763653.1 |
| IA232 | - | - | *taxus* | 46 | 42.0751151 | -93.497193 | Previously published | Kierepka & Latch (2016) | KU763654.1 |
| IA234 | - | - | *taxus* | 2 | 42.0751151 | -93.497193 | Previously published | Kierepka & Latch (2016) | KU763655.1 |
| IA235 | - | - | *taxus* | 82 | 42.0751151 | -93.497193 | Previously published | Kierepka & Latch (2016) | KU763656.1 |
| IA236 | - | - | *taxus* | 82 | 42.0751151 | -93.497193 | Previously published | Kierepka & Latch (2016) | KU763657.1 |
| IA242 | - | - | *taxus* | 87 | 42.0751151 | -93.497193 | Previously published | Kierepka & Latch (2016) | KU763658.1 |
| IA246 | - | - | *taxus* | 1 | 42.0751151 | -93.497193 | Previously published | Kierepka & Latch (2016) | KU763659.1 |
| IA247 | - | - | *taxus* | 1 | 42.0751151 | -93.497193 | Previously published | Kierepka & Latch (2016) | KU763660.1 |
| IA263 | - | - | *taxus* | 1 | 42.0751151 | -93.497193 | Previously published | Kierepka & Latch (2016) | KU763661.1 |
| IA267 | - | - | *taxus* | 1 | 42.0751151 | -93.497193 | Previously published | Kierepka & Latch (2016) | KU763662.1 |
| IA271 | - | - | *taxus* | 2 | 42.0751151 | -93.497193 | Previously published | Kierepka & Latch (2016) | KU763663.1 |
| IA272 | - | - | *taxus* | 2 | 42.0751151 | -93.497193 | Previously published | Kierepka & Latch (2016) | KU763664.1 |
| IA330 | - | - | *taxus* | 8 | 42.0751151 | -93.497193 | Previously published | Kierepka & Latch (2016) | KU763665.1 |
| IA334 | - | - | *taxus* | 82 | 42.0751151 | -93.497193 | Previously published | Kierepka & Latch (2016) | KU763666.1 |
| IA340 | - | - | *taxus* | 1 | 42.0751151 | -93.497193 | Previously published | Kierepka & Latch (2016) | KU763667.1 |
| IA341 | - | - | *taxus* | 1 | 42.0751151 | -93.497193 | Previously published | Kierepka & Latch (2016) | KU763668.1 |
| IA346 | - | - | *taxus* | 1 | 42.0751151 | -93.497193 | Previously published | Kierepka & Latch (2016) | KU763669.1 |
| IA357 | - | - | *taxus* | 87 | 42.0751151 | -93.497193 | Previously published | Kierepka & Latch (2016) | KU763670.1 |
| IA38 | - | - | *taxus* | 1 | 42.0751151 | -93.497193 | Previously published | Kierepka & Latch (2016) | KU763671.1 |
| IA45 | - | - | *taxus* | 2 | 42.0751151 | -93.497193 | Previously published | Kierepka & Latch (2016) | KU763672.1 |
| IA53 | - | - | *taxus* | 45 | 42.0751151 | -93.497193 | Previously published | Kierepka & Latch (2016) | KU763673.1 |
| IA56 | - | - | *taxus* | 1 | 42.0751151 | -93.497193 | Previously published | Kierepka & Latch (2016) | KU763674.1 |
| IA60 | - | - | *taxus* | 2 | 42.0751151 | -93.497193 | Previously published | Kierepka & Latch (2016) | KU763675.1 |
| IA65 | - | - | *taxus* | 1 | 42.0751151 | -93.497193 | Previously published | Kierepka & Latch (2016) | KU763676.1 |
| IA77 | - | - | *taxus* | 1 | 42.0751151 | -93.497193 | Previously published | Kierepka & Latch (2016) | KU763677.1 |
| IA78 | - | - | *taxus* | 1 | 42.0751151 | -93.497193 | Previously published | Kierepka & Latch (2016) | KU763678.1 |
| ID_01 | - | - | *jeffersonii* | 80 | 44.3436562 | -114.61889 | Previously published | Kierepka & Latch (2016) | KU763681.1 |
| ID_02 | - | - | *jeffersonii* | 80 | 44.3436562 | -114.61889 | Previously published | Kierepka & Latch (2016) | KU763682.1 |
| ID_03 | - | - | *jeffersonii* | 80 | 44.3436562 | -114.61889 | Previously published | Kierepka & Latch (2016) | KU763683.1 |
| ID_04 | - | - | *jeffersonii* | 90 | 44.3436562 | -114.61889 | Previously published | Kierepka & Latch (2016) | KU763684.1 |
| ID_05 | - | - | *jeffersonii* | 33 | 44.3436562 | -114.61889 | Previously published | Kierepka & Latch (2016) | KU763685.1 |
| ID_06 | - | - | *jeffersonii* | 7 | 44.3436562 | -114.61889 | Previously published | Kierepka & Latch (2016) | KU763686.1 |
| ID_07 | - | - | *jeffersonii* | 53 | 44.3436562 | -114.61889 | Previously published | Kierepka & Latch (2016) | KU763687.1 |
| ID_08 | - | - | *jeffersonii* | 59 | 44.3436562 | -114.61889 | Previously published | Kierepka & Latch (2016) | KU763688.1 |
| ID_09 | - | - | *jeffersonii* | 33 | 44.3436562 | -114.61889 | Previously published | Kierepka & Latch (2016) | KU763689.1 |
| ID_10 | - | - | *jeffersonii* | 58 | 44.3436562 | -114.61889 | Previously published | Kierepka & Latch (2016) | KU763690.1 |
| ID_11 | - | - | *jeffersonii* | 47 | 44.3436562 | -114.61889 | Previously published | Kierepka & Latch (2016) | KU763691.1 |
| ID_13 | - | - | *jeffersonii* | 90 | 44.3436562 | -114.61889 | Previously published | Kierepka & Latch (2016) | KU763692.1 |
| ID_14 | - | - | *jeffersonii* | 80 | 44.3436562 | -114.61889 | Previously published | Kierepka & Latch (2016) | KU763693.1 |
| ID_15 | - | - | *jeffersonii* | 33 | 44.3436562 | -114.61889 | Previously published | Kierepka & Latch (2016) | KU763694.1 |
| ID_16 | - | - | *jeffersonii* | 90 | 44.3436562 | -114.61889 | Previously published | Kierepka & Latch (2016) | KU763695.1 |
| ID_17 | - | - | *jeffersonii* | 80 | 44.3436562 | -114.61889 | Previously published | Kierepka & Latch (2016) | KU763696.1 |
| ID_18 | - | - | *jeffersonii* | 58 | 44.3436562 | -114.61889 | Previously published | Kierepka & Latch (2016) | KU763697.1 |
| ID_19 | - | - | *jeffersonii* | 86 | 44.3436562 | -114.61889 | Previously published | Kierepka & Latch (2016) | KU763698.1 |
| ID145 | - | - | *jeffersonii* | 34 | 44.3436562 | -114.61889 | Previously published | Kierepka & Latch (2016) | KU763679.1 |
| ID15_7355 | - | - | *jeffersonii* | 52 | 42.75319 | -114.4327 | Previously published | Ford et al. (2019) | MK882760.1 |
| ID15_7356 | - | - | *jeffersonii* | 4 | 42.7643316 | -114.43242 | Previously published | Ford et al. (2019) | MK882761.1 |
| ID15_7357 | - | - | *jeffersonii* | 30 | 42.1735635 | -113.00561 | Previously published | Ford et al. (2019) | MK882762.1 |
| ID15_7387 | - | - | *jeffersonii* | 4 | 47.588791 | -118.46319 | Previously published | Ford et al. (2019) | MK882763.1 |
| ID30 | - | - | *jeffersonii* | 96 | 44.3436562 | -114.61889 | Previously published | Kierepka & Latch (2016) | KU763680.1 |
| IL01 | - | - | *taxus* | 1 | 40.0406497 | -89.202055 | Previously published | Kierepka & Latch (2016) | KU763699.1 |
| IL06 | - | - | *taxus* | 1 | 40.0406497 | -89.202055 | Previously published | Kierepka & Latch (2016) | KU763700.1 |
| IL111 | - | - | *taxus* | 76 | 40.0406497 | -89.202055 | Previously published | Kierepka & Latch (2016) | KU763701.1 |
| IL127 | - | - | *taxus* | 67 | 40.0406497 | -89.202055 | Previously published | Kierepka & Latch (2016) | KU763702.1 |
| IL147 | - | - | *taxus* | 39 | 40.0406497 | -89.202055 | Previously published | Kierepka & Latch (2016) | KU763703.1 |
| IL152 | - | - | *taxus* | 1 | 40.0406497 | -89.202055 | Previously published | Kierepka & Latch (2016) | KU763704.1 |
| IL181 | - | - | *taxus* | 65 | 40.0406497 | -89.202055 | Previously published | Kierepka & Latch (2016) | KU763705.1 |
| IL194 | - | - | *taxus* | 14 | 40.0406497 | -89.202055 | Previously published | Kierepka & Latch (2016) | KU763706.1 |
| IL201 | - | - | *taxus* | 74 | 40.0406497 | -89.202055 | Previously published | Kierepka & Latch (2016) | KU763707.1 |
| IL245 | - | - | *taxus* | 87 | 40.0406497 | -89.202055 | Previously published | Kierepka & Latch (2016) | KU763708.1 |
| IL55 | - | - | *taxus* | 67 | 40.0406497 | -89.202055 | Previously published | Kierepka & Latch (2016) | KU763709.1 |
| IN01 | - | - | *taxus* | 1 | 39.8985685 | -86.281913 | Previously published | Kierepka & Latch (2016) | KU763710.1 |
| IN04 | - | - | *taxus* | 3 | 39.8985685 | -86.281913 | Previously published | Kierepka & Latch (2016) | KU763711.1 |
| IN05 | - | - | *taxus* | 1 | 39.8985685 | -86.281913 | Previously published | Kierepka & Latch (2016) | KU763712.1 |
| IN178 | - | - | *taxus* | 71 | 39.8985685 | -86.281913 | Previously published | Kierepka & Latch (2016) | KU763713.1 |
| KS001 | - | - | *taxus* | 1 | 38.4908704 | -98.381702 | Previously published | Kierepka & Latch (2016) | KU763714.1 |
| KS112 | - | - | *taxus* | 15 | 38.4908704 | -98.381702 | Previously published | Kierepka & Latch (2016) | KU763715.1 |
| KS113 | - | - | *taxus* | 16 | 38.4908704 | -98.381702 | Previously published | Kierepka & Latch (2016) | KU763716.1 |
| KS114 | - | - | *taxus* | 25 | 38.4908704 | -98.381702 | Previously published | Kierepka & Latch (2016) | KU763717.1 |
| KS115 | - | - | *taxus* | 87 | 38.4908704 | -98.381702 | Previously published | Kierepka & Latch (2016) | KU763718.1 |
| KS117 | - | - | *taxus* | 1 | 38.4908704 | -98.381702 | Previously published | Kierepka & Latch (2016) | KU763719.1 |
| KS120 | - | - | *taxus* | 74 | 38.4908704 | -98.381702 | Previously published | Kierepka & Latch (2016) | KU763720.1 |
| KS131 | - | - | *taxus* | 25 | 38.4908704 | -98.381702 | Previously published | Kierepka & Latch (2016) | KU763721.1 |
| KS136 | - | - | *taxus* | 89 | 38.4908704 | -98.381702 | Previously published | Kierepka & Latch (2016) | KU763722.1 |
| KS143 | - | - | *taxus* | 16 | 38.4908704 | -98.381702 | Previously published | Kierepka & Latch (2016) | KU763723.1 |
| KS16 | - | - | *taxus* | 1 | 38.4908704 | -98.381702 | Previously published | Kierepka & Latch (2016) | KU763724.1 |
| KS163 | - | - | *taxus* | 89 | 38.4908704 | -98.381702 | Previously published | Kierepka & Latch (2016) | KU763725.1 |
| KS183 | - | - | *taxus* | 1 | 38.4908704 | -98.381702 | Previously published | Kierepka & Latch (2016) | KU763726.1 |
| KS235 | - | - | *taxus* | 63 | 38.4908704 | -98.381702 | Previously published | Kierepka & Latch (2016) | KU763727.1 |
| KS240 | - | - | *taxus* | 87 | 38.4908704 | -98.381702 | Previously published | Kierepka & Latch (2016) | KU763728.1 |
| KS249 | - | - | *taxus* | 56 | 38.4908704 | -98.381702 | Previously published | Kierepka & Latch (2016) | KU763729.1 |
| KS268 | - | - | *taxus* | 77 | 38.4908704 | -98.381702 | Previously published | Kierepka & Latch (2016) | KU763730.1 |
| KS288 | - | - | *taxus* | 66 | 38.4908704 | -98.381702 | Previously published | Kierepka & Latch (2016) | KU763731.1 |
| KS296 | - | - | *taxus* | 100 | 38.4908704 | -98.381702 | Previously published | Kierepka & Latch (2016) | KU763732.1 |
| KS305 | - | - | *taxus* | 1 | 38.4908704 | -98.381702 | Previously published | Kierepka & Latch (2016) | KU763733.1 |
| KS32 | - | - | *taxus* | 1 | 38.4908704 | -98.381702 | Previously published | Kierepka & Latch (2016) | KU763734.1 |
| KS358 | - | - | *taxus* | 89 | 38.4908704 | -98.381702 | Previously published | Kierepka & Latch (2016) | KU763735.1 |
| KS364 | - | - | *taxus* | 1 | 38.4908704 | -98.381702 | Previously published | Kierepka & Latch (2016) | KU763736.1 |
| KS368 | - | - | *taxus* | 1 | 38.4908704 | -98.381702 | Previously published | Kierepka & Latch (2016) | KU763737.1 |
| KS38 | - | - | *taxus* | 1 | 38.4908704 | -98.381702 | Previously published | Kierepka & Latch (2016) | KU763738.1 |
| KS56 | - | - | *taxus* | 1 | 38.4908704 | -98.381702 | Previously published | Kierepka & Latch (2016) | KU763739.1 |
| KS64 | - | - | *taxus* | 1 | 38.4908704 | -98.381702 | Previously published | Kierepka & Latch (2016) | KU763740.1 |
| KS65 | - | - | *taxus* | 1 | 38.4908704 | -98.381702 | Previously published | Kierepka & Latch (2016) | KU763741.1 |
| KS71 | - | - | *taxus* | 71 | 38.4908704 | -98.381702 | Previously published | Kierepka & Latch (2016) | KU763742.1 |
| KS96 | - | - | *taxus* | 15 | 38.4908704 | -98.381702 | Previously published | Kierepka & Latch (2016) | KU763743.1 |
| KS97 | - | - | *taxus* | 10 | 38.4908704 | -98.381702 | Previously published | Kierepka & Latch (2016) | KU763744.1 |
| LP_0115 | - | - | *jacksoni* | 3 | 43.4626842 | -84.633923 | Previously published | Kierepka & Latch (2016) | KU763745.1 |
| LP_0117 | - | - | *jacksoni* | 3 | 43.4626842 | -84.633923 | Previously published | Kierepka & Latch (2016) | KU763746.1 |
| LP_0124 | - | - | *jacksoni* | 1 | 43.4626842 | -84.633923 | Previously published | Kierepka & Latch (2016) | KU763747.1 |
| LP_0127 | - | - | *jacksoni* | 1 | 43.4626842 | -84.633923 | Previously published | Kierepka & Latch (2016) | KU763748.1 |
| LP_0129 | - | - | *jacksoni* | 1 | 43.4626842 | -84.633923 | Previously published | Kierepka & Latch (2016) | KU763749.1 |
| LP_0134 | - | - | *jacksoni* | 1 | 43.4626842 | -84.633923 | Previously published | Kierepka & Latch (2016) | KU763750.1 |
| LP_0135 | - | - | *jacksoni* | 1 | 43.4626842 | -84.633923 | Previously published | Kierepka & Latch (2016) | KU763751.1 |
| LP_0136 | - | - | *jacksoni* | 3 | 43.4626842 | -84.633923 | Previously published | Kierepka & Latch (2016) | KU763752.1 |
| LP_0138 | - | - | *jacksoni* | 3 | 43.4626842 | -84.633923 | Previously published | Kierepka & Latch (2016) | KU763753.1 |
| LP_0141 | - | - | *jacksoni* | 1 | 43.4626842 | -84.633923 | Previously published | Kierepka & Latch (2016) | KU763754.1 |
| LP_0145 | - | - | *jacksoni* | 1 | 43.4626842 | -84.633923 | Previously published | Kierepka & Latch (2016) | KU763755.1 |
| LP_0147 | - | - | *jacksoni* | 3 | 43.4626842 | -84.633923 | Previously published | Kierepka & Latch (2016) | KU763756.1 |
| LP_0151 | - | - | *jacksoni* | 3 | 43.4626842 | -84.633923 | Previously published | Kierepka & Latch (2016) | KU763757.1 |
| LP_0155 | - | - | *jacksoni* | 1 | 43.4626842 | -84.633923 | Previously published | Kierepka & Latch (2016) | KU763758.1 |
| LP_0156 | - | - | *jacksoni* | 3 | 43.4626842 | -84.633923 | Previously published | Kierepka & Latch (2016) | KU763759.1 |
| LP_0159 | - | - | *jacksoni* | 3 | 43.4626842 | -84.633923 | Previously published | Kierepka & Latch (2016) | KU763760.1 |
| LP_0160 | - | - | *jacksoni* | 3 | 43.4626842 | -84.633923 | Previously published | Kierepka & Latch (2016) | KU763761.1 |
| LP_0161 | - | - | *jacksoni* | 3 | 43.4626842 | -84.633923 | Previously published | Kierepka & Latch (2016) | KU763762.1 |
| LP_0165 | - | - | *jacksoni* | 1 | 43.4626842 | -84.633923 | Previously published | Kierepka & Latch (2016) | KU763763.1 |
| LP_0166 | - | - | *jacksoni* | 3 | 43.4626842 | -84.633923 | Previously published | Kierepka & Latch (2016) | KU763764.1 |
| LP_0168 | - | - | *jacksoni* | 1 | 43.4626842 | -84.633923 | Previously published | Kierepka & Latch (2016) | KU763765.1 |
| LP_0170 | - | - | *jacksoni* | 3 | 43.4626842 | -84.633923 | Previously published | Kierepka & Latch (2016) | KU763766.1 |
| LP_0175 | - | - | *jacksoni* | 1 | 43.4626842 | -84.633923 | Previously published | Kierepka & Latch (2016) | KU763767.1 |
| LP_0178 | - | - | *jacksoni* | 1 | 43.4626842 | -84.633923 | Previously published | Kierepka & Latch (2016) | KU763768.1 |
| LP_0184 | - | - | *jacksoni* | 3 | 43.4626842 | -84.633923 | Previously published | Kierepka & Latch (2016) | KU763769.1 |
| LP_0185 | - | - | *jacksoni* | 1 | 43.4626842 | -84.633923 | Previously published | Kierepka & Latch (2016) | KU763770.1 |
| LP_0187 | - | - | *jacksoni* | 1 | 43.4626842 | -84.633923 | Previously published | Kierepka & Latch (2016) | KU763771.1 |
| LP_0190 | - | - | *jacksoni* | 1 | 43.4626842 | -84.633923 | Previously published | Kierepka & Latch (2016) | KU763772.1 |
| LP_0191 | - | - | *jacksoni* | 1 | 43.4626842 | -84.633923 | Previously published | Kierepka & Latch (2016) | KU763773.1 |
| LP_0197 | - | - | *jacksoni* | 1 | 43.4626842 | -84.633923 | Previously published | Kierepka & Latch (2016) | KU763774.1 |
| LP_0210 | - | - | *jacksoni* | 3 | 43.4626842 | -84.633923 | Previously published | Kierepka & Latch (2016) | KU763775.1 |
| LP_0211 | - | - | *jacksoni* | 3 | 43.4626842 | -84.633923 | Previously published | Kierepka & Latch (2016) | KU763776.1 |
| LP_0213 | - | - | *jacksoni* | 3 | 43.4626842 | -84.633923 | Previously published | Kierepka & Latch (2016) | KU763777.1 |
| LP_0214 | - | - | *jacksoni* | 3 | 43.4626842 | -84.633923 | Previously published | Kierepka & Latch (2016) | KU763778.1 |
| LP_0215 | - | - | *jacksoni* | 3 | 43.4626842 | -84.633923 | Previously published | Kierepka & Latch (2016) | KU763779.1 |
| LP_0216 | - | - | *jacksoni* | 3 | 43.4626842 | -84.633923 | Previously published | Kierepka & Latch (2016) | KU763780.1 |
| LP_0217 | - | - | *jacksoni* | 1 | 43.4626842 | -84.633923 | Previously published | Kierepka & Latch (2016) | KU763781.1 |
| LP_0218 | - | - | *jacksoni* | 1 | 43.4626842 | -84.633923 | Previously published | Kierepka & Latch (2016) | KU763782.1 |
| LP_0219 | - | - | *jacksoni* | 3 | 43.4626842 | -84.633923 | Previously published | Kierepka & Latch (2016) | KU763783.1 |
| LP_0220 | - | - | *jacksoni* | 3 | 43.4626842 | -84.633923 | Previously published | Kierepka & Latch (2016) | KU763784.1 |
| LP_0221 | - | - | *jacksoni* | 1 | 43.4626842 | -84.633923 | Previously published | Kierepka & Latch (2016) | KU763785.1 |
| LP_0222 | - | - | *jacksoni* | 1 | 43.4626842 | -84.633923 | Previously published | Kierepka & Latch (2016) | KU763786.1 |
| LP_0223 | - | - | *jacksoni* | 40 | 43.4626842 | -84.633923 | Previously published | Kierepka & Latch (2016) | KU763787.1 |
| LP_0224 | - | - | *jacksoni* | 3 | 43.4626842 | -84.633923 | Previously published | Kierepka & Latch (2016) | KU763788.1 |
| LP_0227 | - | - | *jacksoni* | 3 | 43.4626842 | -84.633923 | Previously published | Kierepka & Latch (2016) | KU763789.1 |
| LP_0228 | - | - | *jacksoni* | 1 | 43.4626842 | -84.633923 | Previously published | Kierepka & Latch (2016) | KU763790.1 |
| LP_0229 | - | - | *jacksoni* | 3 | 43.4626842 | -84.633923 | Previously published | Kierepka & Latch (2016) | KU763791.1 |
| LP_023 | - | - | *jacksoni* | 3 | 43.4626842 | -84.633923 | Previously published | Kierepka & Latch (2016) | KU763792.1 |
| LP_0230 | - | - | *jacksoni* | 3 | 43.4626842 | -84.633923 | Previously published | Kierepka & Latch (2016) | KU763793.1 |
| LP_0231 | - | - | *jacksoni* | 1 | 43.4626842 | -84.633923 | Previously published | Kierepka & Latch (2016) | KU763794.1 |
| LP_0232 | - | - | *jacksoni* | 3 | 43.4626842 | -84.633923 | Previously published | Kierepka & Latch (2016) | KU763795.1 |
| LP_0233 | - | - | *jacksoni* | 1 | 43.4626842 | -84.633923 | Previously published | Kierepka & Latch (2016) | KU763796.1 |
| LP_0234 | - | - | *jacksoni* | 3 | 43.4626842 | -84.633923 | Previously published | Kierepka & Latch (2016) | KU763797.1 |
| LP_0235 | - | - | *jacksoni* | 3 | 43.4626842 | -84.633923 | Previously published | Kierepka & Latch (2016) | KU763798.1 |
| LP_0236 | - | - | *jacksoni* | 1 | 43.4626842 | -84.633923 | Previously published | Kierepka & Latch (2016) | KU763799.1 |
| LP_0237 | - | - | *jacksoni* | 3 | 43.4626842 | -84.633923 | Previously published | Kierepka & Latch (2016) | KU763800.1 |
| LP_0238 | - | - | *jacksoni* | 3 | 43.4626842 | -84.633923 | Previously published | Kierepka & Latch (2016) | KU763801.1 |
| LP_0239 | - | - | *jacksoni* | 1 | 43.4626842 | -84.633923 | Previously published | Kierepka & Latch (2016) | KU763802.1 |
| LP_024 | - | - | *jacksoni* | 3 | 43.4626842 | -84.633923 | Previously published | Kierepka & Latch (2016) | KU763803.1 |
| LP_0242 | - | - | *jacksoni* | 3 | 43.4626842 | -84.633923 | Previously published | Kierepka & Latch (2016) | KU763804.1 |
| LP_0243 | - | - | *jacksoni* | 3 | 43.4626842 | -84.633923 | Previously published | Kierepka & Latch (2016) | KU763805.1 |
| LP_0244 | - | - | *jacksoni* | 1 | 43.4626842 | -84.633923 | Previously published | Kierepka & Latch (2016) | KU763806.1 |
| LP_0245 | - | - | *jacksoni* | 3 | 43.4626842 | -84.633923 | Previously published | Kierepka & Latch (2016) | KU763807.1 |
| LP_0246 | - | - | *jacksoni* | 3 | 43.4626842 | -84.633923 | Previously published | Kierepka & Latch (2016) | KU763808.1 |
| LP_0251 | - | - | *jacksoni* | 1 | 43.4626842 | -84.633923 | Previously published | Kierepka & Latch (2016) | KU763809.1 |
| LP_0252 | - | - | *jacksoni* | 1 | 43.4626842 | -84.633923 | Previously published | Kierepka & Latch (2016) | KU763810.1 |
| LP_0253 | - | - | *jacksoni* | 3 | 43.4626842 | -84.633923 | Previously published | Kierepka & Latch (2016) | KU763811.1 |
| LP_0254 | - | - | *jacksoni* | 3 | 43.4626842 | -84.633923 | Previously published | Kierepka & Latch (2016) | KU763812.1 |
| LP_0255 | - | - | *jacksoni* | 1 | 43.4626842 | -84.633923 | Previously published | Kierepka & Latch (2016) | KU763813.1 |
| LP_0256 | - | - | *jacksoni* | 1 | 43.4626842 | -84.633923 | Previously published | Kierepka & Latch (2016) | KU763814.1 |
| LP_0257 | - | - | *jacksoni* | 1 | 43.4626842 | -84.633923 | Previously published | Kierepka & Latch (2016) | KU763815.1 |
| LP_0258 | - | - | *jacksoni* | 3 | 43.4626842 | -84.633923 | Previously published | Kierepka & Latch (2016) | KU763816.1 |
| LP_0259 | - | - | *jacksoni* | 3 | 43.4626842 | -84.633923 | Previously published | Kierepka & Latch (2016) | KU763817.1 |
| LP_026 | - | - | *jacksoni* | 3 | 43.4626842 | -84.633923 | Previously published | Kierepka & Latch (2016) | KU763818.1 |
| LP_0260 | - | - | *jacksoni* | 1 | 43.4626842 | -84.633923 | Previously published | Kierepka & Latch (2016) | KU763819.1 |
| LP_0262 | - | - | *jacksoni* | 1 | 43.4626842 | -84.633923 | Previously published | Kierepka & Latch (2016) | KU763820.1 |
| LP_0263 | - | - | *jacksoni* | 1 | 43.4626842 | -84.633923 | Previously published | Kierepka & Latch (2016) | KU763821.1 |
| LP_0264 | - | - | *jacksoni* | 1 | 43.4626842 | -84.633923 | Previously published | Kierepka & Latch (2016) | KU763822.1 |
| LP_0265 | - | - | *jacksoni* | 3 | 43.4626842 | -84.633923 | Previously published | Kierepka & Latch (2016) | KU763823.1 |
| LP_0266 | - | - | *jacksoni* | 3 | 43.4626842 | -84.633923 | Previously published | Kierepka & Latch (2016) | KU763824.1 |
| LP_0267 | - | - | *jacksoni* | 1 | 43.4626842 | -84.633923 | Previously published | Kierepka & Latch (2016) | KU763825.1 |
| LP_0268 | - | - | *jacksoni* | 3 | 43.4626842 | -84.633923 | Previously published | Kierepka & Latch (2016) | KU763826.1 |
| LP_0269 | - | - | *jacksoni* | 3 | 43.4626842 | -84.633923 | Previously published | Kierepka & Latch (2016) | KU763827.1 |
| LP_027 | - | - | *jacksoni* | 3 | 43.4626842 | -84.633923 | Previously published | Kierepka & Latch (2016) | KU763828.1 |
| LP_0270 | - | - | *jacksoni* | 1 | 43.4626842 | -84.633923 | Previously published | Kierepka & Latch (2016) | KU763829.1 |
| LP_0271 | - | - | *jacksoni* | 3 | 43.4626842 | -84.633923 | Previously published | Kierepka & Latch (2016) | KU763830.1 |
| LP_0272 | - | - | *jacksoni* | 1 | 43.4626842 | -84.633923 | Previously published | Kierepka & Latch (2016) | KU763831.1 |
| LP_0273 | - | - | *jacksoni* | 3 | 43.4626842 | -84.633923 | Previously published | Kierepka & Latch (2016) | KU763832.1 |
| LP_0274 | - | - | *jacksoni* | 3 | 43.4626842 | -84.633923 | Previously published | Kierepka & Latch (2016) | KU763833.1 |
| LP_028 | - | - | *jacksoni* | 3 | 43.4626842 | -84.633923 | Previously published | Kierepka & Latch (2016) | KU763834.1 |
| LP_0283 | - | - | *jacksoni* | 1 | 43.4626842 | -84.633923 | Previously published | Kierepka & Latch (2016) | KU763835.1 |
| LP_0284 | - | - | *jacksoni* | 1 | 43.4626842 | -84.633923 | Previously published | Kierepka & Latch (2016) | KU763836.1 |
| LP_0286 | - | - | *jacksoni* | 3 | 43.4626842 | -84.633923 | Previously published | Kierepka & Latch (2016) | KU763837.1 |
| LP_0287 | - | - | *jacksoni* | 3 | 43.4626842 | -84.633923 | Previously published | Kierepka & Latch (2016) | KU763838.1 |
| LP_029 | - | - | *jacksoni* | 1 | 43.4626842 | -84.633923 | Previously published | Kierepka & Latch (2016) | KU763839.1 |
| LP_BALP19 | - | - | *jacksoni* | 39 | 44.850765 | -84.199138 | Previously published | Ethier et al. (2012) | GU901593.1 |
| LP_BAUP5 | - | - | *jacksoni* | 12 | 44.850765 | -84.199138 | Previously published | Ethier et al. (2012) | GU901594.1 |
| LP_LP01_23 | - | - | *jacksoni* | 12 | 44.850765 | -84.199138 | Previously published | Ethier et al. (2012) | GU901581.1 |
| LP_LP0118 | - | - | *jacksoni* | 39 | 44.850765 | -84.199138 | Previously published | Ethier et al. (2012) | GU901590.1 |
| LP_LP0133 | - | - | *jacksoni* | 39 | 44.850765 | -84.199138 | Previously published | Ethier et al. (2012) | GU901589.1 |
| LP_LP0135 | - | - | *jacksoni* | 12 | 44.850765 | -84.199138 | Previously published | Ethier et al. (2012) | GU901591.1 |
| LP_LP0172 | - | - | *jacksoni* | 39 | 44.850765 | -84.199138 | Previously published | Ethier et al. (2012) | GU901585.1 |
| LP_LP02_46 | - | - | *jacksoni* | 39 | 44.850765 | -84.199138 | Previously published | Ethier et al. (2012) | GU901580.1 |
| LP_LP02_89 | - | - | *jacksoni* | 39 | 44.850765 | -84.199138 | Previously published | Ethier et al. (2012) | GU901579.1 |
| LP_LP0203 | - | - | *jacksoni* | 39 | 44.850765 | -84.199138 | Previously published | Ethier et al. (2012) | GU901584.1 |
| LP_LP0206 | - | - | *jacksoni* | 39 | 44.850765 | -84.199138 | Previously published | Ethier et al. (2012) | GU901583.1 |
| LP_LP02106 | - | - | *jacksoni* | 39 | 44.850765 | -84.199138 | Previously published | Ethier et al. (2012) | GU901578.1 |
| LP_LP0242 | - | - | *jacksoni* | 39 | 44.850765 | -84.199138 | Previously published | Ethier et al. (2012) | GU901588.1 |
| LP_LP0259 | - | - | *jacksoni* | 39 | 44.850765 | -84.199138 | Previously published | Ethier et al. (2012) | GU901587.1 |
| LP_LP0283 | - | - | *jacksoni* | 12 | 44.850765 | -84.199138 | Previously published | Ethier et al. (2012) | GU901582.1 |
| LP_LP0286 | - | - | *jacksoni* | 39 | 44.850765 | -84.199138 | Previously published | Ethier et al. (2012) | GU901586.1 |
| LP_MI196 | - | - | *jacksoni* | 1 | 43.4626842 | -84.633923 | Previously published | Kierepka & Latch (2016) | KU763840.1 |
| LP_MI4 | - | - | *jacksoni* | 1 | 43.4626842 | -84.633923 | Previously published | Kierepka & Latch (2016) | KU763841.1 |
| LP_MI51 | - | - | *jacksoni* | 3 | 43.4626842 | -84.633923 | Previously published | Kierepka & Latch (2016) | KU763842.1 |
| LP_MI86 | - | - | *jacksoni* | 3 | 43.4626842 | -84.633923 | Previously published | Kierepka & Latch (2016) | KU763843.1 |
| LP_MIBA_lp81 | - | - | *jacksoni* | 39 | 44.850765 | -84.199138 | Previously published | Ethier et al. (2012) | GU901592.1 |
| LP_MIBA_lp88r | - | - | *jacksoni* | 12 | 44.850765 | -84.199138 | Previously published | Ethier et al. (2012) | GU901577.1 |
| LP_MIBA_up80 | - | - | *jacksoni* | 12 | 46.329041 | -85.989556 | Previously published | Ethier et al. (2012) | GU901576.1 |
| MB_4ILR157 | - | - | *taxus* | 2 | 49.444858 | -98.191389 | Previously published | Ethier et al. (2012) | GU901657.1 |
| MB_4ILR756 | - | - | *taxus* | 109 | 49.444858 | -98.191389 | Previously published | Ethier et al. (2012) | GU901656.1 |
| MB_4ILR763 | - | - | *taxus* | 39 | 49.444858 | -98.191389 | Previously published | Ethier et al. (2012) | GU901655.1 |
| MB_4ILR956 | - | - | *taxus* | 2 | 49.444858 | -98.191389 | Previously published | Ethier et al. (2012) | GU901654.1 |
| MB_4ILR957 | - | - | *taxus* | 39 | 49.444858 | -98.191389 | Previously published | Ethier et al. (2012) | GU901662.1 |
| MB_4ILS034 | - | - | *taxus* | 2 | 49.444858 | -98.191389 | Previously published | Ethier et al. (2012) | GU901653.1 |
| MB_4ILS106 | - | - | *taxus* | 48 | 49.444858 | -98.191389 | Previously published | Ethier et al. (2012) | GU901652.1 |
| MB_4ILS107 | - | - | *taxus* | 109 | 49.444858 | -98.191389 | Previously published | Ethier et al. (2012) | GU901651.1 |
| MB_4ILS108 | - | - | *taxus* | 109 | 49.444858 | -98.191389 | Previously published | Ethier et al. (2012) | GU901661.1 |
| MB_4ILS164 | - | - | *taxus* | 2 | 49.444858 | -98.191389 | Previously published | Ethier et al. (2012) | GU901650.1 |
| MB_4ILS529 | - | - | *taxus* | 12 | 49.444858 | -98.191389 | Previously published | Ethier et al. (2012) | GU901649.1 |
| MB_4ILS530 | - | - | *taxus* | 12 | 49.444858 | -98.191389 | Previously published | Ethier et al. (2012) | GU901648.1 |
| MB_4ILT974 | - | - | *taxus* | 39 | 49.444858 | -98.191389 | Previously published | Ethier et al. (2012) | GU901647.1 |
| MB_4ILU463 | - | - | *taxus* | 39 | 49.444858 | -98.191389 | Previously published | Ethier et al. (2012) | GU901646.1 |
| MB_4ILU698 | - | - | *taxus* | 109 | 49.444858 | -98.191389 | Previously published | Ethier et al. (2012) | GU901660.1 |
| MB_4ILV017 | - | - | *taxus* | 104 | 49.444858 | -98.191389 | Previously published | Ethier et al. (2012) | GU901659.1 |
| MB_4ILV019 | - | - | *taxus* | 109 | 49.444858 | -98.191389 | Previously published | Ethier et al. (2012) | GU901658.1 |
| MB_4ILV081 | - | - | *taxus* | 39 | 49.444858 | -98.191389 | Previously published | Ethier et al. (2012) | GU901645.1 |
| MB_4ILV082 | - | - | *taxus* | 48 | 49.444858 | -98.191389 | Previously published | Ethier et al. (2012) | GU901644.1 |
| MB_4ILV083 | - | - | *taxus* | 50 | 49.444858 | -98.191389 | Previously published | Ethier et al. (2012) | GU901643.1 |
| MB_4ILV084 | - | - | *taxus* | 50 | 49.444858 | -98.191389 | Previously published | Ethier et al. (2012) | GU901642.1 |
| MB_4ILV085 | - | - | *taxus* | 12 | 49.444858 | -98.191389 | Previously published | Ethier et al. (2012) | GU901641.1 |
| MB_4ILV086 | - | - | *taxus* | 2 | 49.444858 | -98.191389 | Previously published | Ethier et al. (2012) | GU901640.1 |
| MB_4ILV395 | - | - | *taxus* | 2 | 49.444858 | -98.191389 | Previously published | Ethier et al. (2012) | GU901639.1 |
| MB_4ILV397 | - | - | *taxus* | 12 | 49.444858 | -98.191389 | Previously published | Ethier et al. (2012) | GU901638.1 |
| MB_4ILX512 | - | - | *taxus* | 12 | 49.444858 | -98.191389 | Previously published | Ethier et al. (2012) | GU901663.1 |
| MB_4ILX655 | - | - | *taxus* | 2 | 49.444858 | -98.191389 | Previously published | Ethier et al. (2012) | GU901637.1 |
| MB_4IMU042 | - | - | *taxus* | 50 | 49.444858 | -98.191389 | Previously published | Ethier et al. (2012) | GU901636.1 |
| MB_4IMV015 | - | - | *taxus* | 107 | 49.444858 | -98.191389 | Previously published | Ethier et al. (2012) | GU901635.1 |
| MB_4IMV016 | - | - | *taxus* | 12 | 49.444858 | -98.191389 | Previously published | Ethier et al. (2012) | GU901634.1 |
| MB_4IMV017_40_ | - | - | *taxus* | 2 | 49.444858 | -98.191389 | Previously published | Ethier et al. (2012) | GU901664.1 |
| MB_4IMV019 | - | - | *taxus* | 50 | 49.444858 | -98.191389 | Previously published | Ethier et al. (2012) | GU901665.1 |
| MB_4IMW044 | - | - | *taxus* | 50 | 49.444858 | -98.191389 | Previously published | Ethier et al. (2012) | GU901633.1 |
| MB_4IMW052 | - | - | *taxus* | 50 | 49.444858 | -98.191389 | Previously published | Ethier et al. (2012) | GU901632.1 |
| MB_4IMW668 | - | - | *taxus* | 39 | 49.444858 | -98.191389 | Previously published | Ethier et al. (2012) | GU901631.1 |
| MB_4IMY161 | - | - | *taxus* | 2 | 49.444858 | -98.191389 | Previously published | Ethier et al. (2012) | GU901630.1 |
| MB_4IMY162 | - | - | *taxus* | 48 | 49.444858 | -98.191389 | Previously published | Ethier et al. (2012) | GU901629.1 |
| MB_4IMY163 | - | - | *taxus* | 111 | 49.444858 | -98.191389 | Previously published | Ethier et al. (2012) | GU901628.1 |
| MB_4IMY444 | - | - | *taxus* | 39 | 49.444858 | -98.191389 | Previously published | Ethier et al. (2012) | GU901627.1 |
| MB_4IMY446 | - | - | *taxus* | 44 | 49.444858 | -98.191389 | Previously published | Ethier et al. (2012) | GU901626.1 |
| MB_4IMZ439 | - | - | *taxus* | 44 | 49.444858 | -98.191389 | Previously published | Ethier et al. (2012) | GU901666.1 |
| MB_4INB903 | - | - | *taxus* | 2 | 49.444858 | -98.191389 | Previously published | Ethier et al. (2012) | GU901667.1 |
| MB_4IND294 | - | - | *taxus* | 2 | 49.444858 | -98.191389 | Previously published | Ethier et al. (2012) | GU901668.1 |
| MB_4IND973 | - | - | *taxus* | 50 | 49.444858 | -98.191389 | Previously published | Ethier et al. (2012) | GU901625.1 |
| MB_4IND974 | - | - | *taxus* | 39 | 49.444858 | -98.191389 | Previously published | Ethier et al. (2012) | GU901622.1 |
| MB_4INE839 | - | - | *taxus* | 50 | 49.444858 | -98.191389 | Previously published | Ethier et al. (2012) | GU901669.1 |
| MB_ILN474 | - | - | *taxus* | 2 | 49.444858 | -98.191389 | Previously published | Ethier et al. (2012) | GU901624.1 |
| MB_ILP157 | - | - | *taxus* | 2 | 49.444858 | -98.191389 | Previously published | Ethier et al. (2012) | GU901521.1 |
| MB_ILP574 | - | - | *taxus* | 12 | 49.444858 | -98.191389 | Previously published | Ethier et al. (2012) | GU901522.1 |
| MB_ILP575 | - | - | *taxus* | 2 | 49.444858 | -98.191389 | Previously published | Ethier et al. (2012) | GU901520.1 |
| MB_ILP953 | - | - | *taxus* | 2 | 49.444858 | -98.191389 | Previously published | Ethier et al. (2012) | GU901519.1 |
| MB_ILQ468 | - | - | *taxus* | 2 | 49.444858 | -98.191389 | Previously published | Ethier et al. (2012) | GU901518.1 |
| MB_ILQ488 | - | - | *taxus* | 106 | 49.444858 | -98.191389 | Previously published | Ethier et al. (2012) | GU901623.1 |
| MB_ILQ902 | - | - | *taxus* | 2 | 49.444858 | -98.191389 | Previously published | Ethier et al. (2012) | GU901517.1 |
| MB_ILR156 | - | - | *taxus* | 2 | 49.444858 | -98.191389 | Previously published | Ethier et al. (2012) | GU901516.1 |
| MB_IRL102 | - | - | *taxus* | 2 | 49.444858 | -98.191389 | Previously published | Ethier et al. (2012) | GU901515.1 |
| MB213 | - | - | *taxus* | 89 | 50.0573753 | -98.88867 | Previously published | Kierepka & Latch (2016) | KU763586.1 |
| MB215 | - | - | *taxus* | 2 | 50.0573753 | -98.88867 | Previously published | Kierepka & Latch (2016) | KU763587.1 |
| MB230 | - | - | *taxus* | 2 | 50.0573753 | -98.88867 | Previously published | Kierepka & Latch (2016) | KU763588.1 |
| MB233 | - | - | *taxus* | 2 | 50.0573753 | -98.88867 | Previously published | Kierepka & Latch (2016) | KU763589.1 |
| MN02 | - | - | *taxus* | 96 | 46.2753613 | -94.302644 | Previously published | Kierepka & Latch (2016) | KU763844.1 |
| MN08 | - | - | *taxus* | 83 | 46.2753613 | -94.302644 | Previously published | Kierepka & Latch (2016) | KU763903.1 |
| MN104 | - | - | *taxus* | 2 | 46.2753613 | -94.302644 | Previously published | Kierepka & Latch (2016) | KU763845.1 |
| MN114 | - | - | *taxus* | 1 | 46.2753613 | -94.302644 | Previously published | Kierepka & Latch (2016) | KU763846.1 |
| MN116 | - | - | *taxus* | 80 | 46.2753613 | -94.302644 | Previously published | Kierepka & Latch (2016) | KU763847.1 |
| MN127 | - | - | *taxus* | 1 | 46.2753613 | -94.302644 | Previously published | Kierepka & Latch (2016) | KU763848.1 |
| MN138 | - | - | *taxus* | 1 | 46.2753613 | -94.302644 | Previously published | Kierepka & Latch (2016) | KU763849.1 |
| MN140 | - | - | *taxus* | 2 | 46.2753613 | -94.302644 | Previously published | Kierepka & Latch (2016) | KU763850.1 |
| MN142 | - | - | *taxus* | 82 | 46.2753613 | -94.302644 | Previously published | Kierepka & Latch (2016) | KU763851.1 |
| MN149 | - | - | *taxus* | 65 | 46.2753613 | -94.302644 | Previously published | Kierepka & Latch (2016) | KU763852.1 |
| MN150 | - | - | *taxus* | 1 | 46.2753613 | -94.302644 | Previously published | Kierepka & Latch (2016) | KU763853.1 |
| MN152 | - | - | *taxus* | 2 | 46.2753613 | -94.302644 | Previously published | Kierepka & Latch (2016) | KU763854.1 |
| MN155 | - | - | *taxus* | 2 | 46.2753613 | -94.302644 | Previously published | Kierepka & Latch (2016) | KU763855.1 |
| MN159 | - | - | *taxus* | 2 | 46.2753613 | -94.302644 | Previously published | Kierepka & Latch (2016) | KU763856.1 |
| MN166 | - | - | *taxus* | 46 | 46.2753613 | -94.302644 | Previously published | Kierepka & Latch (2016) | KU763857.1 |
| MN170 | - | - | *taxus* | 1 | 46.2753613 | -94.302644 | Previously published | Kierepka & Latch (2016) | KU763858.1 |
| MN171 | - | - | *taxus* | 2 | 46.2753613 | -94.302644 | Previously published | Kierepka & Latch (2016) | KU763859.1 |
| MN173 | - | - | *taxus* | 2 | 46.2753613 | -94.302644 | Previously published | Kierepka & Latch (2016) | KU763860.1 |
| MN174 | - | - | *taxus* | 45 | 46.2753613 | -94.302644 | Previously published | Kierepka & Latch (2016) | KU763861.1 |
| MN178 | - | - | *taxus* | 1 | 46.2753613 | -94.302644 | Previously published | Kierepka & Latch (2016) | KU763862.1 |
| MN180 | - | - | *taxus* | 1 | 46.2753613 | -94.302644 | Previously published | Kierepka & Latch (2016) | KU763863.1 |
| MN186 | - | - | *taxus* | 2 | 46.2753613 | -94.302644 | Previously published | Kierepka & Latch (2016) | KU763864.1 |
| MN187 | - | - | *taxus* | 45 | 46.2753613 | -94.302644 | Previously published | Kierepka & Latch (2016) | KU763865.1 |
| MN188 | - | - | *taxus* | 1 | 46.2753613 | -94.302644 | Previously published | Kierepka & Latch (2016) | KU763866.1 |
| MN190 | - | - | *taxus* | 1 | 46.2753613 | -94.302644 | Previously published | Kierepka & Latch (2016) | KU763867.1 |
| MN191 | - | - | *taxus* | 1 | 46.2753613 | -94.302644 | Previously published | Kierepka & Latch (2016) | KU763868.1 |
| MN195 | - | - | *taxus* | 73 | 46.2753613 | -94.302644 | Previously published | Kierepka & Latch (2016) | KU763869.1 |
| MN198 | - | - | *taxus* | 2 | 46.2753613 | -94.302644 | Previously published | Kierepka & Latch (2016) | KU763870.1 |
| MN199 | - | - | *taxus* | 1 | 46.2753613 | -94.302644 | Previously published | Kierepka & Latch (2016) | KU763871.1 |
| MN210 | - | - | *taxus* | 45 | 46.2753613 | -94.302644 | Previously published | Kierepka & Latch (2016) | KU763872.1 |
| MN211 | - | - | *taxus* | 1 | 46.2753613 | -94.302644 | Previously published | Kierepka & Latch (2016) | KU763873.1 |
| MN213 | - | - | *taxus* | 1 | 46.2753613 | -94.302644 | Previously published | Kierepka & Latch (2016) | KU763874.1 |
| MN214 | - | - | *taxus* | 46 | 46.2753613 | -94.302644 | Previously published | Kierepka & Latch (2016) | KU763875.1 |
| MN218 | - | - | *taxus* | 26 | 46.2753613 | -94.302644 | Previously published | Kierepka & Latch (2016) | KU763876.1 |
| MN225 | - | - | *taxus* | 1 | 46.2753613 | -94.302644 | Previously published | Kierepka & Latch (2016) | KU763877.1 |
| MN226 | - | - | *taxus* | 1 | 46.2753613 | -94.302644 | Previously published | Kierepka & Latch (2016) | KU763878.1 |
| MN23 | - | - | *taxus* | 82 | 46.2753613 | -94.302644 | Previously published | Kierepka & Latch (2016) | KU763879.1 |
| MN231 | - | - | *taxus* | 1 | 46.2753613 | -94.302644 | Previously published | Kierepka & Latch (2016) | KU763880.1 |
| MN232 | - | - | *taxus* | 1 | 46.2753613 | -94.302644 | Previously published | Kierepka & Latch (2016) | KU763881.1 |
| MN24_10 | - | - | *taxus* | 84 | 46.2753613 | -94.302644 | Previously published | Kierepka & Latch (2016) | KU763882.1 |
| MN24_12 | - | - | *taxus* | 73 | 46.2753613 | -94.302644 | Previously published | Kierepka & Latch (2016) | KU763883.1 |
| MN26 | - | - | *taxus* | 2 | 46.2753613 | -94.302644 | Previously published | Kierepka & Latch (2016) | KU763884.1 |
| MN32 | - | - | *taxus* | 1 | 46.2753613 | -94.302644 | Previously published | Kierepka & Latch (2016) | KU763885.1 |
| MN325 | - | - | *taxus* | 1 | 46.2753613 | -94.302644 | Previously published | Kierepka & Latch (2016) | KU763886.1 |
| MN326 | - | - | *taxus* | 71 | 46.2753613 | -94.302644 | Previously published | Kierepka & Latch (2016) | KU763887.1 |
| MN328 | - | - | *taxus* | 26 | 46.2753613 | -94.302644 | Previously published | Kierepka & Latch (2016) | KU763888.1 |
| MN337 | - | - | *taxus* | 1 | 46.2753613 | -94.302644 | Previously published | Kierepka & Latch (2016) | KU763889.1 |
| MN338 | - | - | *taxus* | 1 | 46.2753613 | -94.302644 | Previously published | Kierepka & Latch (2016) | KU763890.1 |
| MN339 | - | - | *taxus* | 1 | 46.2753613 | -94.302644 | Previously published | Kierepka & Latch (2016) | KU763891.1 |
| MN34 | - | - | *taxus* | 1 | 46.2753613 | -94.302644 | Previously published | Kierepka & Latch (2016) | KU763892.1 |
| MN342 | - | - | *taxus* | 1 | 46.2753613 | -94.302644 | Previously published | Kierepka & Latch (2016) | KU763893.1 |
| MN42 | - | - | *taxus* | 88 | 46.2753613 | -94.302644 | Previously published | Kierepka & Latch (2016) | KU763894.1 |
| MN43 | - | - | *taxus* | 1 | 46.2753613 | -94.302644 | Previously published | Kierepka & Latch (2016) | KU763895.1 |
| MN44 | - | - | *taxus* | 2 | 46.2753613 | -94.302644 | Previously published | Kierepka & Latch (2016) | KU763896.1 |
| MN50 | - | - | *taxus* | 26 | 46.2753613 | -94.302644 | Previously published | Kierepka & Latch (2016) | KU763897.1 |
| MN55 | - | - | *taxus* | 1 | 46.2753613 | -94.302644 | Previously published | Kierepka & Latch (2016) | KU763898.1 |
| MN68 | - | - | *taxus* | 25 | 46.2753613 | -94.302644 | Previously published | Kierepka & Latch (2016) | KU763899.1 |
| MN73 | - | - | *taxus* | 84 | 46.2753613 | -94.302644 | Previously published | Kierepka & Latch (2016) | KU763900.1 |
| MN74 | - | - | *taxus* | 2 | 46.2753613 | -94.302644 | Previously published | Kierepka & Latch (2016) | KU763901.1 |
| MN76 | - | - | *taxus* | 48 | 46.2753613 | -94.302644 | Previously published | Kierepka & Latch (2016) | KU763902.1 |
| MN80 | - | - | *taxus* | 1 | 46.2753613 | -94.302644 | Previously published | Kierepka & Latch (2016) | KU763904.1 |
| MN81 | - | - | *taxus* | 93 | 46.2753613 | -94.302644 | Previously published | Kierepka & Latch (2016) | KU763905.1 |
| MN83 | - | - | *taxus* | 73 | 46.2753613 | -94.302644 | Previously published | Kierepka & Latch (2016) | KU763906.1 |
| MN85 | - | - | *taxus* | 1 | 46.2753613 | -94.302644 | Previously published | Kierepka & Latch (2016) | KU763907.1 |
| MN93 | - | - | *taxus* | 2 | 46.2753613 | -94.302644 | Previously published | Kierepka & Latch (2016) | KU763908.1 |
| MN97 | - | - | *taxus* | 26 | 46.2753613 | -94.302644 | Previously published | Kierepka & Latch (2016) | KU763909.1 |
| MN98 | - | - | *taxus* | 1 | 46.2753613 | -94.302644 | Previously published | Kierepka & Latch (2016) | KU763910.1 |
| MO110 | - | - | *taxus* | 80 | 38.6071591 | -92.730097 | Previously published | Kierepka & Latch (2016) | KU763911.1 |
| MO146 | - | - | *taxus* | 1 | 38.6071591 | -92.730097 | Previously published | Kierepka & Latch (2016) | KU763912.1 |
| MO165 | - | - | *taxus* | 1 | 38.6071591 | -92.730097 | Previously published | Kierepka & Latch (2016) | KU763913.1 |
| MO205 | - | - | *taxus* | 1 | 38.6071591 | -92.730097 | Previously published | Kierepka & Latch (2016) | KU763914.1 |
| MO215 | - | - | *taxus* | 58 | 38.6071591 | -92.730097 | Previously published | Kierepka & Latch (2016) | KU763915.1 |
| MO239 | - | - | *taxus* | 1 | 38.6071591 | -92.730097 | Previously published | Kierepka & Latch (2016) | KU763916.1 |
| MO240 | - | - | *taxus* | 47 | 38.6071591 | -92.730097 | Previously published | Kierepka & Latch (2016) | KU763917.1 |
| MO242 | - | - | *taxus* | 1 | 38.6071591 | -92.730097 | Previously published | Kierepka & Latch (2016) | KU763918.1 |
| MT_001 | - | - | *taxus* | 2 | 47.0422427 | -109.64229 | Previously published | Kierepka & Latch (2016) | KU763968.1 |
| MT_002 | - | - | *taxus* | 2 | 47.0422427 | -109.64229 | Previously published | Kierepka & Latch (2016) | KU763969.1 |
| MT_003 | - | - | *taxus* | 73 | 47.0422427 | -109.64229 | Previously published | Kierepka & Latch (2016) | KU763970.1 |
| MT_004 | - | - | *taxus* | 2 | 47.0422427 | -109.64229 | Previously published | Kierepka & Latch (2016) | KU763971.1 |
| MT_005 | - | - | *taxus* | 2 | 47.0422427 | -109.64229 | Previously published | Kierepka & Latch (2016) | KU763972.1 |
| MT_006 | - | - | *taxus* | 2 | 47.0422427 | -109.64229 | Previously published | Kierepka & Latch (2016) | KU763973.1 |
| MT_Montanabadger1 | - | - | *jeffersonii* | 2 | 48.433075 | -111.01089 | Previously published | Ethier et al. (2012) | GU901473.1 |
| MT_Montanabadger4 | - | - | *jeffersonii* | 2 | 48.433075 | -111.01089 | Previously published | Ethier et al. (2012) | GU901472.1 |
| MT_Montanabadger6 | - | - | *jeffersonii* | 105 | 48.433075 | -111.01089 | Previously published | Ethier et al. (2012) | GU901474.1 |
| MT_MT_BA10 | - | - | *jeffersonii* | 2 | 48.433075 | -111.01089 | Previously published | Ethier et al. (2012) | GU901480.1 |
| MT_MT_BA15 | - | - | *jeffersonii* | 44 | 48.433075 | -111.01089 | Previously published | Ethier et al. (2012) | GU901479.1 |
| MT_MT_BA16 | - | - | *jeffersonii* | 44 | 48.433075 | -111.01089 | Previously published | Ethier et al. (2012) | GU901476.1 |
| MT_MT_BA17 | - | - | *jeffersonii* | 31 | 48.433075 | -111.01089 | Previously published | Ethier et al. (2012) | GU901478.1 |
| MT_MT_BA18 | - | - | *jeffersonii* | 52 | 48.433075 | -111.01089 | Previously published | Ethier et al. (2012) | GU901475.1 |
| MT_MT_BA20 | - | - | *jeffersonii* | 2 | 48.433075 | -111.01089 | Previously published | Ethier et al. (2012) | GU901477.1 |
| MT_MT_BA21 | - | - | *jeffersonii* | 44 | 48.433075 | -111.01089 | Previously published | Ethier et al. (2012) | GU901482.1 |
| MT_MT_BA9 | - | - | *jeffersonii* | 2 | 48.433075 | -111.01089 | Previously published | Ethier et al. (2012) | GU901481.1 |
| MT_MTBA12a | - | - | *jeffersonii* | 2 | 48.433075 | -111.01089 | Previously published | Ethier et al. (2012) | GU901483.1 |
| MT100_10 | - | - | *taxus* | 71 | 47.0422427 | -109.64229 | Previously published | Kierepka & Latch (2016) | KU763919.1 |
| MT100_12 | - | - | *taxus* | 2 | 47.0422427 | -109.64229 | Previously published | Kierepka & Latch (2016) | KU763920.1 |
| MT102 | - | - | *taxus* | 2 | 47.0422427 | -109.64229 | Previously published | Kierepka & Latch (2016) | KU763921.1 |
| MT106 | - | - | *taxus* | 1 | 47.0422427 | -109.64229 | Previously published | Kierepka & Latch (2016) | KU763922.1 |
| MT107 | - | - | *taxus* | 49 | 47.0422427 | -109.64229 | Previously published | Kierepka & Latch (2016) | KU763923.1 |
| MT109 | - | - | *taxus* | 2 | 47.0422427 | -109.64229 | Previously published | Kierepka & Latch (2016) | KU763924.1 |
| MT111 | - | - | *taxus* | 2 | 47.0422427 | -109.64229 | Previously published | Kierepka & Latch (2016) | KU763925.1 |
| MT119 | - | - | *taxus* | 1 | 47.0422427 | -109.64229 | Previously published | Kierepka & Latch (2016) | KU763926.1 |
| MT123 | - | - | *taxus* | 2 | 47.0422427 | -109.64229 | Previously published | Kierepka & Latch (2016) | KU763927.1 |
| MT124 | - | - | *taxus* | 2 | 47.0422427 | -109.64229 | Previously published | Kierepka & Latch (2016) | KU763928.1 |
| MT126 | - | - | *taxus* | 2 | 47.0422427 | -109.64229 | Previously published | Kierepka & Latch (2016) | KU763929.1 |
| MT130 | - | - | *taxus* | 1 | 47.0422427 | -109.64229 | Previously published | Kierepka & Latch (2016) | KU763930.1 |
| MT131 | - | - | *taxus* | 2 | 47.0422427 | -109.64229 | Previously published | Kierepka & Latch (2016) | KU763931.1 |
| MT132 | - | - | *taxus* | 2 | 47.0422427 | -109.64229 | Previously published | Kierepka & Latch (2016) | KU763932.1 |
| MT134 | - | - | *taxus* | 2 | 47.0422427 | -109.64229 | Previously published | Kierepka & Latch (2016) | KU763933.1 |
| MT135 | - | - | *taxus* | 2 | 47.0422427 | -109.64229 | Previously published | Kierepka & Latch (2016) | KU763934.1 |
| MT137 | - | - | *taxus* | 2 | 47.0422427 | -109.64229 | Previously published | Kierepka & Latch (2016) | KU763935.1 |
| MT138 | - | - | *taxus* | 2 | 47.0422427 | -109.64229 | Previously published | Kierepka & Latch (2016) | KU763936.1 |
| MT14 | - | - | *taxus* | 65 | 47.0422427 | -109.64229 | Previously published | Kierepka & Latch (2016) | KU763937.1 |
| MT143 | - | - | *taxus* | 2 | 47.0422427 | -109.64229 | Previously published | Kierepka & Latch (2016) | KU763938.1 |
| MT145 | - | - | *taxus* | 2 | 47.0422427 | -109.64229 | Previously published | Kierepka & Latch (2016) | KU763939.1 |
| MT147 | - | - | *taxus* | 68 | 47.0422427 | -109.64229 | Previously published | Kierepka & Latch (2016) | KU763940.1 |
| MT15_7359 | - | - | *jeffersonii* | 2 | 46.869765 | -113.97869 | Previously published | Ford et al. (2019) | MK882764.1 |
| MT15_7360 | - | - | *jeffersonii* | 2 | 48.8514094 | -113.30516 | Previously published | Ford et al. (2019) | MK882765.1 |
| MT151 | - | - | *taxus* | 2 | 47.0422427 | -109.64229 | Previously published | Kierepka & Latch (2016) | KU763941.1 |
| MT153 | - | - | *taxus* | 2 | 47.0422427 | -109.64229 | Previously published | Kierepka & Latch (2016) | KU763942.1 |
| MT154 | - | - | *taxus* | 36 | 47.0422427 | -109.64229 | Previously published | Kierepka & Latch (2016) | KU763943.1 |
| MT158 | - | - | *taxus* | 1 | 47.0422427 | -109.64229 | Previously published | Kierepka & Latch (2016) | KU763944.1 |
| MT16 | - | - | *taxus* | 1 | 47.0422427 | -109.64229 | Previously published | Kierepka & Latch (2016) | KU763945.1 |
| MT166 | - | - | *taxus* | 31 | 47.0422427 | -109.64229 | Previously published | Kierepka & Latch (2016) | KU763946.1 |
| MT17 | - | - | *taxus* | 43 | 47.0422427 | -109.64229 | Previously published | Kierepka & Latch (2016) | KU763947.1 |
| MT18 | - | - | *taxus* | 1 | 47.0422427 | -109.64229 | Previously published | Kierepka & Latch (2016) | KU763948.1 |
| MT236 | - | - | *taxus* | 2 | 47.0422427 | -109.64229 | Previously published | Kierepka & Latch (2016) | KU763949.1 |
| MT32613 | - | - | *jeffersonii* | 2 | 45.349705 | -111.89205 | Previously published | Ford et al. (2019) | MK882766.1 |
| MT34 | - | - | *taxus* | 71 | 47.0422427 | -109.64229 | Previously published | Kierepka & Latch (2016) | KU763950.1 |
| MT35 | - | - | *taxus* | 2 | 47.0422427 | -109.64229 | Previously published | Kierepka & Latch (2016) | KU763951.1 |
| MT37 | - | - | *taxus* | 2 | 47.0422427 | -109.64229 | Previously published | Kierepka & Latch (2016) | KU763952.1 |
| MT40 | - | - | *taxus* | 2 | 47.0422427 | -109.64229 | Previously published | Kierepka & Latch (2016) | KU763953.1 |
| MT49 | - | - | *taxus* | 31 | 47.0422427 | -109.64229 | Previously published | Kierepka & Latch (2016) | KU763954.1 |
| MT54 | - | - | *taxus* | 1 | 47.0422427 | -109.64229 | Previously published | Kierepka & Latch (2016) | KU763955.1 |
| MT57 | - | - | *taxus* | 48 | 47.0422427 | -109.64229 | Previously published | Kierepka & Latch (2016) | KU763956.1 |
| MT60 | - | - | *taxus* | 2 | 47.0422427 | -109.64229 | Previously published | Kierepka & Latch (2016) | KU763957.1 |
| MT63 | - | - | *taxus* | 69 | 47.0422427 | -109.64229 | Previously published | Kierepka & Latch (2016) | KU763958.1 |
| MT66 | - | - | *taxus* | 69 | 47.0422427 | -109.64229 | Previously published | Kierepka & Latch (2016) | KU763959.1 |
| MT67 | - | - | *taxus* | 2 | 47.0422427 | -109.64229 | Previously published | Kierepka & Latch (2016) | KU763960.1 |
| MT70 | - | - | *taxus* | 80 | 47.0422427 | -109.64229 | Previously published | Kierepka & Latch (2016) | KU763961.1 |
| MT84 | - | - | *taxus* | 1 | 47.0422427 | -109.64229 | Previously published | Kierepka & Latch (2016) | KU763962.1 |
| MT85 | - | - | *taxus* | 2 | 47.0422427 | -109.64229 | Previously published | Kierepka & Latch (2016) | KU763963.1 |
| MT89 | - | - | *taxus* | 1 | 47.0422427 | -109.64229 | Previously published | Kierepka & Latch (2016) | KU763964.1 |
| MT92 | - | - | *taxus* | 101 | 47.0422427 | -109.64229 | Previously published | Kierepka & Latch (2016) | KU763965.1 |
| MT95 | - | - | *taxus* | 2 | 47.0422427 | -109.64229 | Previously published | Kierepka & Latch (2016) | KU763966.1 |
| MT99 | - | - | *taxus* | 96 | 47.0422427 | -109.64229 | Previously published | Kierepka & Latch (2016) | KU763967.1 |
| ND02 | - | - | *taxus* | 84 | 47.4461179 | -100.46762 | Previously published | Kierepka & Latch (2016) | KU763974.1 |
| ND03 | - | - | *taxus* | 1 | 47.4461179 | -100.46762 | Previously published | Kierepka & Latch (2016) | KU763975.1 |
| ND09 | - | - | *taxus* | 3 | 47.4461179 | -100.46762 | Previously published | Kierepka & Latch (2016) | KU763976.1 |
| ND103 | - | - | *taxus* | 2 | 47.4461179 | -100.46762 | Previously published | Kierepka & Latch (2016) | KU763977.1 |
| ND108 | - | - | *taxus* | 3 | 47.4461179 | -100.46762 | Previously published | Kierepka & Latch (2016) | KU763978.1 |
| ND112 | - | - | *taxus* | 9 | 47.4461179 | -100.46762 | Previously published | Kierepka & Latch (2016) | KU763979.1 |
| ND113 | - | - | *taxus* | 1 | 47.4461179 | -100.46762 | Previously published | Kierepka & Latch (2016) | KU763980.1 |
| ND116 | - | - | *taxus* | 1 | 47.4461179 | -100.46762 | Previously published | Kierepka & Latch (2016) | KU763981.1 |
| ND117 | - | - | *taxus* | 1 | 47.4461179 | -100.46762 | Previously published | Kierepka & Latch (2016) | KU763982.1 |
| ND120 | - | - | *taxus* | 2 | 47.4461179 | -100.46762 | Previously published | Kierepka & Latch (2016) | KU763983.1 |
| ND121 | - | - | *taxus* | 2 | 47.4461179 | -100.46762 | Previously published | Kierepka & Latch (2016) | KU763984.1 |
| ND128 | - | - | *taxus* | 3 | 47.4461179 | -100.46762 | Previously published | Kierepka & Latch (2016) | KU763985.1 |
| ND129 | - | - | *taxus* | 91 | 47.4461179 | -100.46762 | Previously published | Kierepka & Latch (2016) | KU763986.1 |
| ND139 | - | - | *taxus* | 2 | 47.4461179 | -100.46762 | Previously published | Kierepka & Latch (2016) | KU763987.1 |
| ND140 | - | - | *taxus* | 1 | 47.4461179 | -100.46762 | Previously published | Kierepka & Latch (2016) | KU763988.1 |
| ND148 | - | - | *taxus* | 72 | 47.4461179 | -100.46762 | Previously published | Kierepka & Latch (2016) | KU763989.1 |
| ND149 | - | - | *taxus* | 1 | 47.4461179 | -100.46762 | Previously published | Kierepka & Latch (2016) | KU763990.1 |
| ND160 | - | - | *taxus* | 94 | 47.4461179 | -100.46762 | Previously published | Kierepka & Latch (2016) | KU763991.1 |
| ND161 | - | - | *taxus* | 50 | 47.4461179 | -100.46762 | Previously published | Kierepka & Latch (2016) | KU763992.1 |
| ND162_10 | - | - | *taxus* | 2 | 47.4461179 | -100.46762 | Previously published | Kierepka & Latch (2016) | KU763993.1 |
| ND162_12 | - | - | *taxus* | 2 | 47.4461179 | -100.46762 | Previously published | Kierepka & Latch (2016) | KU763994.1 |
| ND164 | - | - | *taxus* | 23 | 47.4461179 | -100.46762 | Previously published | Kierepka & Latch (2016) | KU763995.1 |
| ND168 | - | - | *taxus* | 3 | 47.4461179 | -100.46762 | Previously published | Kierepka & Latch (2016) | KU763996.1 |
| ND17 | - | - | *taxus* | 26 | 47.4461179 | -100.46762 | Previously published | Kierepka & Latch (2016) | KU763997.1 |
| ND170 | - | - | *taxus* | 3 | 47.4461179 | -100.46762 | Previously published | Kierepka & Latch (2016) | KU763998.1 |
| ND172_10 | - | - | *taxus* | 3 | 47.4461179 | -100.46762 | Previously published | Kierepka & Latch (2016) | KU763999.1 |
| ND172_12 | - | - | *taxus* | 2 | 47.4461179 | -100.46762 | Previously published | Kierepka & Latch (2016) | KU764000.1 |
| ND216 | - | - | *taxus* | 2 | 47.4461179 | -100.46762 | Previously published | Kierepka & Latch (2016) | KU764001.1 |
| ND29 | - | - | *taxus* | 1 | 47.4461179 | -100.46762 | Previously published | Kierepka & Latch (2016) | KU764002.1 |
| ND30 | - | - | *taxus* | 2 | 47.4461179 | -100.46762 | Previously published | Kierepka & Latch (2016) | KU764003.1 |
| ND327 | - | - | *taxus* | 2 | 47.4461179 | -100.46762 | Previously published | Kierepka & Latch (2016) | KU764004.1 |
| ND332 | - | - | *taxus* | 2 | 47.4461179 | -100.46762 | Previously published | Kierepka & Latch (2016) | KU764005.1 |
| ND333 | - | - | *taxus* | 22 | 47.4461179 | -100.46762 | Previously published | Kierepka & Latch (2016) | KU764006.1 |
| ND335 | - | - | *taxus* | 22 | 47.4461179 | -100.46762 | Previously published | Kierepka & Latch (2016) | KU764007.1 |
| ND343 | - | - | *taxus* | 2 | 47.4461179 | -100.46762 | Previously published | Kierepka & Latch (2016) | KU764008.1 |
| ND344 | - | - | *taxus* | 2 | 47.4461179 | -100.46762 | Previously published | Kierepka & Latch (2016) | KU764009.1 |
| ND345 | - | - | *taxus* | 50 | 47.4461179 | -100.46762 | Previously published | Kierepka & Latch (2016) | KU764010.1 |
| ND351 | - | - | *taxus* | 2 | 47.4461179 | -100.46762 | Previously published | Kierepka & Latch (2016) | KU764011.1 |
| ND36 | - | - | *taxus* | 41 | 47.4461179 | -100.46762 | Previously published | Kierepka & Latch (2016) | KU764012.1 |
| ND37 | - | - | *taxus* | 1 | 47.4461179 | -100.46762 | Previously published | Kierepka & Latch (2016) | KU764013.1 |
| ND46 | - | - | *taxus* | 2 | 47.4461179 | -100.46762 | Previously published | Kierepka & Latch (2016) | KU764014.1 |
| ND48 | - | - | *taxus* | 73 | 47.4461179 | -100.46762 | Previously published | Kierepka & Latch (2016) | KU764015.1 |
| ND52 | - | - | *taxus* | 2 | 47.4461179 | -100.46762 | Previously published | Kierepka & Latch (2016) | KU764016.1 |
| ND58 | - | - | *taxus* | 3 | 47.4461179 | -100.46762 | Previously published | Kierepka & Latch (2016) | KU764017.1 |
| ND64 | - | - | *taxus* | 2 | 47.4461179 | -100.46762 | Previously published | Kierepka & Latch (2016) | KU764018.1 |
| ND69 | - | - | *taxus* | 2 | 47.4461179 | -100.46762 | Previously published | Kierepka & Latch (2016) | KU764019.1 |
| ND72 | - | - | *taxus* | 1 | 47.4461179 | -100.46762 | Previously published | Kierepka & Latch (2016) | KU764020.1 |
| ND75 | - | - | *taxus* | 2 | 47.4461179 | -100.46762 | Previously published | Kierepka & Latch (2016) | KU764021.1 |
| ND81 | - | - | *taxus* | 2 | 47.4461179 | -100.46762 | Previously published | Kierepka & Latch (2016) | KU764022.1 |
| ND82 | - | - | *taxus* | 3 | 47.4461179 | -100.46762 | Previously published | Kierepka & Latch (2016) | KU764023.1 |
| ND83 | - | - | *taxus* | 3 | 47.4461179 | -100.46762 | Previously published | Kierepka & Latch (2016) | KU764024.1 |
| ND86 | - | - | *taxus* | 48 | 47.4461179 | -100.46762 | Previously published | Kierepka & Latch (2016) | KU764025.1 |
| ND88 | - | - | *taxus* | 2 | 47.4461179 | -100.46762 | Previously published | Kierepka & Latch (2016) | KU764026.1 |
| ND90 | - | - | *taxus* | 3 | 47.4461179 | -100.46762 | Previously published | Kierepka & Latch (2016) | KU764027.1 |
| ND91 | - | - | *taxus* | 2 | 47.4461179 | -100.46762 | Previously published | Kierepka & Latch (2016) | KU764028.1 |
| ND92 | - | - | *taxus* | 101 | 47.4461179 | -100.46762 | Previously published | Kierepka & Latch (2016) | KU764029.1 |
| ND94 | - | - | *taxus* | 2 | 47.4461179 | -100.46762 | Previously published | Kierepka & Latch (2016) | KU764030.1 |
| ND99 | - | - | *taxus* | 2 | 47.4461179 | -100.46762 | Previously published | Kierepka & Latch (2016) | KU764031.1 |
| NE01 | - | - | *taxus* | 1 | 41.5316946 | -99.792553 | Previously published | Kierepka & Latch (2016) | KU764032.1 |
| NE10 | - | - | *taxus* | 1 | 41.5316946 | -99.792553 | Previously published | Kierepka & Latch (2016) | KU764033.1 |
| NE11 | - | - | *taxus* | 67 | 41.5316946 | -99.792553 | Previously published | Kierepka & Latch (2016) | KU764034.1 |
| NE115 | - | - | *taxus* | 85 | 41.5316946 | -99.792553 | Previously published | Kierepka & Latch (2016) | KU764035.1 |
| NE12 | - | - | *taxus* | 99 | 41.5316946 | -99.792553 | Previously published | Kierepka & Latch (2016) | KU764036.1 |
| NE122 | - | - | *taxus* | 2 | 41.5316946 | -99.792553 | Previously published | Kierepka & Latch (2016) | KU764037.1 |
| NE134 | - | - | *taxus* | 89 | 41.5316946 | -99.792553 | Previously published | Kierepka & Latch (2016) | KU764038.1 |
| NE137 | - | - | *taxus* | 1 | 41.5316946 | -99.792553 | Previously published | Kierepka & Latch (2016) | KU764039.1 |
| NE15_10 | - | - | *taxus* | 65 | 41.5316946 | -99.792553 | Previously published | Kierepka & Latch (2016) | KU764041.1 |
| NE15_12 | - | - | *taxus* | 89 | 41.5316946 | -99.792553 | Previously published | Kierepka & Latch (2016) | KU764042.1 |
| NE150 | - | - | *taxus* | 95 | 41.5316946 | -99.792553 | Previously published | Kierepka & Latch (2016) | KU764040.1 |
| NE169 | - | - | *taxus* | 1 | 41.5316946 | -99.792553 | Previously published | Kierepka & Latch (2016) | KU764043.1 |
| NE182_10 | - | - | *taxus* | 1 | 41.5316946 | -99.792553 | Previously published | Kierepka & Latch (2016) | KU764044.1 |
| NE182_12 | - | - | *taxus* | 88 | 41.5316946 | -99.792553 | Previously published | Kierepka & Latch (2016) | KU764045.1 |
| NE184 | - | - | *taxus* | 1 | 41.5316946 | -99.792553 | Previously published | Kierepka & Latch (2016) | KU764046.1 |
| NE185 | - | - | *taxus* | 89 | 41.5316946 | -99.792553 | Previously published | Kierepka & Latch (2016) | KU764047.1 |
| NE19 | - | - | *taxus* | 48 | 41.5316946 | -99.792553 | Previously published | Kierepka & Latch (2016) | KU764048.1 |
| NE20 | - | - | *taxus* | 1 | 41.5316946 | -99.792553 | Previously published | Kierepka & Latch (2016) | KU764049.1 |
| NE204 | - | - | *taxus* | 2 | 41.5316946 | -99.792553 | Previously published | Kierepka & Latch (2016) | KU764050.1 |
| NE21 | - | - | *taxus* | 88 | 41.5316946 | -99.792553 | Previously published | Kierepka & Latch (2016) | KU764051.1 |
| NE219 | - | - | *taxus* | 1 | 41.5316946 | -99.792553 | Previously published | Kierepka & Latch (2016) | KU764052.1 |
| NE220 | - | - | *taxus* | 87 | 41.5316946 | -99.792553 | Previously published | Kierepka & Latch (2016) | KU764053.1 |
| NE221 | - | - | *taxus* | 37 | 41.5316946 | -99.792553 | Previously published | Kierepka & Latch (2016) | KU764054.1 |
| NE223 | - | - | *taxus* | 3 | 41.5316946 | -99.792553 | Previously published | Kierepka & Latch (2016) | KU764055.1 |
| NE224 | - | - | *taxus* | 45 | 41.5316946 | -99.792553 | Previously published | Kierepka & Latch (2016) | KU764056.1 |
| NE233 | - | - | *taxus* | 48 | 41.5316946 | -99.792553 | Previously published | Kierepka & Latch (2016) | KU764057.1 |
| NE243_10 | - | - | *taxus* | 1 | 41.5316946 | -99.792553 | Previously published | Kierepka & Latch (2016) | KU764058.1 |
| NE243_12 | - | - | *taxus* | 83 | 41.5316946 | -99.792553 | Previously published | Kierepka & Latch (2016) | KU764059.1 |
| NE244 | - | - | *taxus* | 60 | 41.5316946 | -99.792553 | Previously published | Kierepka & Latch (2016) | KU764060.1 |
| NE245 | - | - | *taxus* | 1 | 41.5316946 | -99.792553 | Previously published | Kierepka & Latch (2016) | KU764061.1 |
| NE250 | - | - | *taxus* | 1 | 41.5316946 | -99.792553 | Previously published | Kierepka & Latch (2016) | KU764062.1 |
| NE252 | - | - | *taxus* | 71 | 41.5316946 | -99.792553 | Previously published | Kierepka & Latch (2016) | KU764063.1 |
| NE254 | - | - | *taxus* | 88 | 41.5316946 | -99.792553 | Previously published | Kierepka & Latch (2016) | KU764064.1 |
| NE255 | - | - | *taxus* | 88 | 41.5316946 | -99.792553 | Previously published | Kierepka & Latch (2016) | KU764065.1 |
| NE256 | - | - | *taxus* | 1 | 41.5316946 | -99.792553 | Previously published | Kierepka & Latch (2016) | KU764066.1 |
| NE257 | - | - | *taxus* | 1 | 41.5316946 | -99.792553 | Previously published | Kierepka & Latch (2016) | KU764067.1 |
| NE259 | - | - | *taxus* | 37 | 41.5316946 | -99.792553 | Previously published | Kierepka & Latch (2016) | KU764068.1 |
| NE261 | - | - | *taxus* | 48 | 41.5316946 | -99.792553 | Previously published | Kierepka & Latch (2016) | KU764069.1 |
| NE262 | - | - | *taxus* | 26 | 41.5316946 | -99.792553 | Previously published | Kierepka & Latch (2016) | KU764070.1 |
| NE266 | - | - | *taxus* | 65 | 41.5316946 | -99.792553 | Previously published | Kierepka & Latch (2016) | KU764071.1 |
| NE27_10 | - | - | *taxus* | 1 | 41.5316946 | -99.792553 | Previously published | Kierepka & Latch (2016) | KU764074.1 |
| NE27_12 | - | - | *taxus* | 1 | 41.5316946 | -99.792553 | Previously published | Kierepka & Latch (2016) | KU764075.1 |
| NE273 | - | - | *taxus* | 92 | 41.5316946 | -99.792553 | Previously published | Kierepka & Latch (2016) | KU764072.1 |
| NE276 | - | - | *taxus* | 62 | 41.5316946 | -99.792553 | Previously published | Kierepka & Latch (2016) | KU764073.1 |
| NE281 | - | - | *taxus* | 1 | 41.5316946 | -99.792553 | Previously published | Kierepka & Latch (2016) | KU764076.1 |
| NE289 | - | - | *taxus* | 48 | 41.5316946 | -99.792553 | Previously published | Kierepka & Latch (2016) | KU764077.1 |
| NE291 | - | - | *taxus* | 46 | 41.5316946 | -99.792553 | Previously published | Kierepka & Latch (2016) | KU764078.1 |
| NE294 | - | - | *taxus* | 1 | 41.5316946 | -99.792553 | Previously published | Kierepka & Latch (2016) | KU764079.1 |
| NE302 | - | - | *taxus* | 98 | 41.5316946 | -99.792553 | Previously published | Kierepka & Latch (2016) | KU764080.1 |
| NE31 | - | - | *taxus* | 3 | 41.5316946 | -99.792553 | Previously published | Kierepka & Latch (2016) | KU764081.1 |
| NE33 | - | - | *taxus* | 1 | 41.5316946 | -99.792553 | Previously published | Kierepka & Latch (2016) | KU764082.1 |
| NE331 | - | - | *taxus* | 1 | 41.5316946 | -99.792553 | Previously published | Kierepka & Latch (2016) | KU764083.1 |
| NE336 | - | - | *taxus* | 92 | 41.5316946 | -99.792553 | Previously published | Kierepka & Latch (2016) | KU764084.1 |
| NE359 | - | - | *taxus* | 1 | 41.5316946 | -99.792553 | Previously published | Kierepka & Latch (2016) | KU764085.1 |
| NE365 | - | - | *taxus* | 1 | 41.5316946 | -99.792553 | Previously published | Kierepka & Latch (2016) | KU764086.1 |
| NE366 | - | - | *taxus* | 1 | 41.5316946 | -99.792553 | Previously published | Kierepka & Latch (2016) | KU764087.1 |
| NE369 | - | - | *taxus* | 55 | 41.5316946 | -99.792553 | Previously published | Kierepka & Latch (2016) | KU764088.1 |
| NE39_10 | - | - | *taxus* | 3 | 41.5316946 | -99.792553 | Previously published | Kierepka & Latch (2016) | KU764089.1 |
| NE39_12 | - | - | *taxus* | 1 | 41.5316946 | -99.792553 | Previously published | Kierepka & Latch (2016) | KU764090.1 |
| NE47 | - | - | *taxus* | 88 | 41.5316946 | -99.792553 | Previously published | Kierepka & Latch (2016) | KU764091.1 |
| NI10_2573 | - | - | *jeffersonii* | 2 | 50.133429 | -120.83854 | Previously published | Ford et al. (2019) | MK882767.1 |
| NI15_6149 | - | - | *jeffersonii* | 4 | 49.4251603 | -120.56384 | Previously published | Ford et al. (2019) | MK882768.1 |
| NI15_6297 | - | - | *jeffersonii* | 4 | 49.9516954 | -120.617 | Previously published | Ford et al. (2019) | MK882769.1 |
| NI15_7421 | - | - | *jeffersonii* | 4 | 49.4251603 | -120.56384 | Previously published | Ford et al. (2019) | MK882770.1 |
| NM001 | - | - | *berlandieri* | 11 | 34.4121746 | -106.10669 | Previously published | Kierepka & Latch (2016) | Direct from authors |
| NM002 | - | - | *berlandieri* | 80 | 34.4121746 | -106.10669 | Previously published | Kierepka & Latch (2016) | Direct from authors |
| NM003 | - | - | *berlandieri* | 4 | 34.4121746 | -106.10669 | Previously published | Kierepka & Latch (2016) | Direct from authors |
| NM004 | - | - | *berlandieri* | 80 | 34.4121746 | -106.10669 | Previously published | Kierepka & Latch (2016) | Direct from authors |
| NM005 | - | - | *berlandieri* | 80 | 34.4121746 | -106.10669 | Previously published | Kierepka & Latch (2016) | Direct from authors |
| NM006 | - | - | *berlandieri* | 70 | 34.4121746 | -106.10669 | Previously published | Kierepka & Latch (2016) | Direct from authors |
| NM007 | - | - | *berlandieri* | 62 | 34.4121746 | -106.10669 | Previously published | Kierepka & Latch (2016) | Direct from authors |
| NM008 | - | - | *berlandieri* | 80 | 34.4121746 | -106.10669 | Previously published | Kierepka & Latch (2016) | Direct from authors |
| NM009 | - | - | *berlandieri* | 80 | 34.4121746 | -106.10669 | Previously published | Kierepka & Latch (2016) | Direct from authors |
| NM010 | - | - | *berlandieri* | 80 | 34.4121746 | -106.10669 | Previously published | Kierepka & Latch (2016) | KU764103.1 |
| NM141 | - | - | *berlandieri* | 62 | 34.4121746 | -106.10669 | Previously published | Kierepka & Latch (2016) | KU764104.1 |
| OH03 | - | - | *jacksoni* | 89 | 40.3566727 | -83.78846 | Previously published | Kierepka & Latch (2016) | KU764105.1 |
| OH1 | - | - | *jacksoni* | 3 | 40.3566727 | -83.78846 | Previously published | Kierepka & Latch (2016) | KU764106.1 |
| OH11 | - | - | *jacksoni* | 1 | 40.3566727 | -83.78846 | Previously published | Kierepka & Latch (2016) | KU764107.1 |
| OH12 | - | - | *jacksoni* | 3 | 40.3566727 | -83.78846 | Previously published | Kierepka & Latch (2016) | KU764108.1 |
| OH13 | - | - | *jacksoni* | 3 | 40.3566727 | -83.78846 | Previously published | Kierepka & Latch (2016) | KU764109.1 |
| OH14 | - | - | *jacksoni* | 3 | 40.3566727 | -83.78846 | Previously published | Kierepka & Latch (2016) | KU764110.1 |
| OH15 | - | - | *jacksoni* | 3 | 40.3566727 | -83.78846 | Previously published | Kierepka & Latch (2016) | KU764111.1 |
| OH16 | - | - | *jacksoni* | 3 | 40.3566727 | -83.78846 | Previously published | Kierepka & Latch (2016) | KU764112.1 |
| OH18 | - | - | *jacksoni* | 3 | 40.3566727 | -83.78846 | Previously published | Kierepka & Latch (2016) | KU764113.1 |
| OH19 | - | - | *jacksoni* | 3 | 40.3566727 | -83.78846 | Previously published | Kierepka & Latch (2016) | KU764114.1 |
| OH2 | - | - | *jacksoni* | 3 | 40.3566727 | -83.78846 | Previously published | Kierepka & Latch (2016) | KU764115.1 |
| OH20 | - | - | *jacksoni* | 3 | 40.3566727 | -83.78846 | Previously published | Kierepka & Latch (2016) | KU764116.1 |
| OH21 | - | - | *jacksoni* | 3 | 40.3566727 | -83.78846 | Previously published | Kierepka & Latch (2016) | KU764117.1 |
| OH23 | - | - | *jacksoni* | 1 | 40.3566727 | -83.78846 | Previously published | Kierepka & Latch (2016) | KU764118.1 |
| OH24 | - | - | *jacksoni* | 3 | 40.3566727 | -83.78846 | Previously published | Kierepka & Latch (2016) | KU764119.1 |
| OH25 | - | - | *jacksoni* | 1 | 40.3566727 | -83.78846 | Previously published | Kierepka & Latch (2016) | KU764120.1 |
| OH27 | - | - | *jacksoni* | 3 | 40.3566727 | -83.78846 | Previously published | Kierepka & Latch (2016) | KU764121.1 |
| OH29 | - | - | *jacksoni* | 3 | 40.3566727 | -83.78846 | Previously published | Kierepka & Latch (2016) | KU764122.1 |
| OH30 | - | - | *jacksoni* | 89 | 40.3566727 | -83.78846 | Previously published | Kierepka & Latch (2016) | KU764123.1 |
| OH31 | - | - | *jacksoni* | 3 | 40.3566727 | -83.78846 | Previously published | Kierepka & Latch (2016) | KU764124.1 |
| OH34 | - | - | *jacksoni* | 3 | 40.3566727 | -83.78846 | Previously published | Kierepka & Latch (2016) | KU764125.1 |
| OH4 | - | - | *jacksoni* | 3 | 40.3566727 | -83.78846 | Previously published | Kierepka & Latch (2016) | KU764126.1 |
| OH5 | - | - | *jacksoni* | 1 | 40.3566727 | -83.78846 | Previously published | Kierepka & Latch (2016) | KU764127.1 |
| OH6 | - | - | *jacksoni* | 3 | 40.3566727 | -83.78846 | Previously published | Kierepka & Latch (2016) | KU764128.1 |
| OH66 | - | - | *jacksoni* | 77 | 40.3566727 | -83.78846 | Previously published | Kierepka & Latch (2016) | KU764129.1 |
| OH7 | - | - | *jacksoni* | 3 | 40.3566727 | -83.78846 | Previously published | Kierepka & Latch (2016) | KU764130.1 |
| OH8 | - | - | *jacksoni* | 3 | 40.3566727 | -83.78846 | Previously published | Kierepka & Latch (2016) | KU764131.1 |
| OH9 | - | - | *jacksoni* | 3 | 40.3566727 | -83.78846 | Previously published | Kierepka & Latch (2016) | KU764132.1 |
| OK001 | - | - | *berlandieri* | 88 | 49.6939775 | -119.15347 | Previously published | Kierepka & Latch (2016) | KU764133.1 |
| OK002 | - | - | *berlandieri* | 102 | 49.6939775 | -119.15347 | Previously published | Kierepka & Latch (2016) | KU764134.1 |
| OK003 | - | - | *berlandieri* | 1 | 49.6939775 | -119.15347 | Previously published | Kierepka & Latch (2016) | KU764135.1 |
| OK004 | - | - | *berlandieri* | 1 | 49.6939775 | -119.15347 | Previously published | Kierepka & Latch (2016) | KU764136.1 |
| OK0040 | - | - | *jeffersonii* | 58 | 49.025922 | -119.24521 | Previously published | Ford et al. (2019) | MK882771.1 |
| OK0042 | - | - | *jeffersonii* | 4 | 49.457813 | -120.46538 | Previously published | Ford et al. (2019) | MK882772.1 |
| OK0043 | - | - | *jeffersonii* | 58 | 49.0154 | -119.24453 | Previously published | Ford et al. (2019) | MK882773.1 |
| OK0046 | - | - | *jeffersonii* | 4 | 49.90151 | -120.10007 | Previously published | Ford et al. (2019) | MK882774.1 |
| OK005 | - | - | *berlandieri* | 1 | 49.6939775 | -119.15347 | Previously published | Kierepka & Latch (2016) | KU764137.1 |
| OK006 | - | - | *berlandieri* | 61 | 49.6939775 | -119.15347 | Previously published | Kierepka & Latch (2016) | KU764138.1 |
| OK007 | - | - | *berlandieri* | 1 | 49.6939775 | -119.15347 | Previously published | Kierepka & Latch (2016) | KU764139.1 |
| OK008 | - | - | *berlandieri* | 67 | 49.6939775 | -119.15347 | Previously published | Kierepka & Latch (2016) | KU764140.1 |
| OK009 | - | - | *berlandieri* | 67 | 49.6939775 | -119.15347 | Previously published | Kierepka & Latch (2016) | KU764141.1 |
| OK010 | - | - | *berlandieri* | 1 | 49.6939775 | -119.15347 | Previously published | Kierepka & Latch (2016) | KU764142.1 |
| OK02_Dyer | - | - | *jeffersonii* | 4 | 49.6939775 | -119.15347 | Previously published | Ford et al. (2019) | MK882775.1 |
| OK0401 | - | - | *jeffersonii* | 4 | 49.937308 | -119.39493 | Previously published | Ford et al. (2019) | MK882776.1 |
| OK0407 | - | - | *jeffersonii* | 58 | 49.03735 | -119.14582 | Previously published | Ford et al. (2019) | MK882777.1 |
| OK0423 | - | - | *jeffersonii* | 4 | 49.935475 | -120.0695 | Previously published | Ford et al. (2019) | MK882778.1 |
| OK0428 | - | - | *jeffersonii* | 58 | 49.010452 | -119.13265 | Previously published | Ford et al. (2019) | MK882779.1 |
| OK10_2457 | - | - | *jeffersonii* | 4 | 50.4516423 | -119.44768 | Previously published | Ford et al. (2019) | MK882780.1 |
| OK10_2578 | - | - | *jeffersonii* | 4 | 50.2393795 | -118.61409 | Previously published | Ford et al. (2019) | MK882781.1 |
| OK12_3551 | - | - | *jeffersonii* | 44 | 50.2243133 | -119.24114 | Previously published | Ford et al. (2019) | MK882782.1 |
| OK15_6249 | - | - | *jeffersonii* | 44 | 50.2645949 | -119.27076 | Previously published | Ford et al. (2019) | MK882783.1 |
| OK15_6964 | - | - | *jeffersonii* | 4 | 50.056291 | -119.40795 | Previously published | Ford et al. (2019) | MK882784.1 |
| OK15_7395 | - | - | *jeffersonii* | 2 | 50.250855 | -118.89693 | Previously published | Ford et al. (2019) | MK882785.1 |
| OK15_7401 | - | - | *jeffersonii* | 4 | 50.454497 | -119.44278 | Previously published | Ford et al. (2019) | MK882786.1 |
| OK15_7424 | - | - | *jeffersonii* | 4 | 50.056291 | -119.40795 | Previously published | Ford et al. (2019) | MK882787.1 |
| OK16_8479 | - | - | *jeffersonii* | 4 | 50.248946 | -118.70623 | Previously published | Ford et al. (2019) | MK882788.1 |
| OK16_8489 | - | - | *jeffersonii* | 4 | 50.233442 | -119.00923 | Previously published | Ford et al. (2019) | MK882789.1 |
| OK16_8491 | - | - | *jeffersonii* | 4 | 49.937002 | -119.39488 | Previously published | Ford et al. (2019) | MK882790.1 |
| OK16_8492 | - | - | *jeffersonii* | 4 | 49.928339 | -119.38832 | Previously published | Ford et al. (2019) | MK882791.1 |
| OK16_8494 | - | - | *jeffersonii* | 4 | 49.939295 | -119.38907 | Previously published | Ford et al. (2019) | MK882792.1 |
| OK16_8495 | - | - | *jeffersonii* | 4 | 49.939352 | -119.38941 | Previously published | Ford et al. (2019) | MK882793.1 |
| OK16_8496 | - | - | *jeffersonii* | 4 | 49.94328 | -119.3871 | Previously published | Ford et al. (2019) | MK882794.1 |
| OK16_8497 | - | - | *jeffersonii* | 4 | 49.939295 | -119.38907 | Previously published | Ford et al. (2019) | MK882795.1 |
| OK202 | - | - | *berlandieri* | 35 | 49.6939775 | -119.15347 | Previously published | Kierepka & Latch (2016) | KU764143.1 |
| OK61 | - | - | *berlandieri* | 2 | 49.6939775 | -119.15347 | Previously published | Kierepka & Latch (2016) | KU764144.1 |
| ON_AB12 | - | - | *jacksoni* | 39 | 43.416884 | -80.783217 | Previously published | Ethier et al. (2012) | GU901546.1 |
| ON_AB13 | - | - | *jacksoni* | 39 | 43.416884 | -80.783217 | Previously published | Ethier et al. (2012) | GU901545.1 |
| ON_AB18 | - | - | *jacksoni* | 39 | 43.416884 | -80.783217 | Previously published | Ethier et al. (2012) | GU901547.1 |
| ON_FT1 | - | - | *jacksoni* | 39 | 43.416884 | -80.783217 | Previously published | Ethier et al. (2012) | GU901558.1 |
| ON_FT2 | - | - | *jacksoni* | 39 | 43.416884 | -80.783217 | Previously published | Ethier et al. (2012) | GU901557.1 |
| ON_FT23 | - | - | *jacksoni* | 39 | 43.416884 | -80.783217 | Previously published | Ethier et al. (2012) | GU901556.1 |
| ON_FT27 | - | - | *jacksoni* | 39 | 43.416884 | -80.783217 | Previously published | Ethier et al. (2012) | GU901555.1 |
| ON_FT30 | - | - | *jacksoni* | 39 | 43.416884 | -80.783217 | Previously published | Ethier et al. (2012) | GU901554.1 |
| ON_FT34 | - | - | *jacksoni* | 39 | 43.416884 | -80.783217 | Previously published | Ethier et al. (2012) | GU901553.1 |
| ON_FT39 | - | - | *jacksoni* | 39 | 43.416884 | -80.783217 | Previously published | Ethier et al. (2012) | GU901552.1 |
| ON_FT46 | - | - | *jacksoni* | 39 | 43.416884 | -80.783217 | Previously published | Ethier et al. (2012) | GU901551.1 |
| ON_FT61 | - | - | *jacksoni* | 39 | 43.416884 | -80.783217 | Previously published | Ethier et al. (2012) | GU901550.1 |
| ON_FT79 | - | - | *jacksoni* | 39 | 43.416884 | -80.783217 | Previously published | Ethier et al. (2012) | GU901549.1 |
| ON_FT80 | - | - | *jacksoni* | 39 | 43.416884 | -80.783217 | Previously published | Ethier et al. (2012) | GU901548.1 |
| ON_ON239_2_ON | - | - | *jacksoni* | 39 | 43.416884 | -80.783217 | Previously published | Ethier et al. (2012) | GU901533.1 |
| ON_ON252_2_ | - | - | *jacksoni* | 39 | 43.416884 | -80.783217 | Previously published | Ethier et al. (2012) | GU901544.1 |
| ON_ON266 | - | - | *jacksoni* | 39 | 43.416884 | -80.783217 | Previously published | Ethier et al. (2012) | GU901534.1 |
| ON_ON277 | - | - | *jacksoni* | 39 | 43.416884 | -80.783217 | Previously published | Ethier et al. (2012) | GU901542.1 |
| ON_ON279 | - | - | *jacksoni* | 39 | 43.416884 | -80.783217 | Previously published | Ethier et al. (2012) | GU901541.1 |
| ON_ON280 | - | - | *jacksoni* | 39 | 43.416884 | -80.783217 | Previously published | Ethier et al. (2012) | GU901540.1 |
| ON_ON282 | - | - | *jacksoni* | 39 | 43.416884 | -80.783217 | Previously published | Ethier et al. (2012) | GU901539.1 |
| ON_ON284 | - | - | *jacksoni* | 39 | 43.416884 | -80.783217 | Previously published | Ethier et al. (2012) | GU901538.1 |
| ON_ON286 | - | - | *jacksoni* | 12 | 43.416884 | -80.783217 | Previously published | Ethier et al. (2012) | GU901543.1 |
| ON_ON288 | - | - | *jacksoni* | 39 | 43.416884 | -80.783217 | Previously published | Ethier et al. (2012) | GU901537.1 |
| ON_ON289 | - | - | *jacksoni* | 39 | 43.416884 | -80.783217 | Previously published | Ethier et al. (2012) | GU901536.1 |
| ON_ON291 | - | - | *jacksoni* | 39 | 43.416884 | -80.783217 | Previously published | Ethier et al. (2012) | GU901535.1 |
| OR_02 | - | - | *jeffersonii* | 32 | 43.926387 | -120.56049 | Previously published | Kierepka & Latch (2016) | KU764146.1 |
| OR_03 | - | - | *jeffersonii* | 5 | 43.926387 | -120.56049 | Previously published | Kierepka & Latch (2016) | KU764147.1 |
| OR_04 | - | - | *jeffersonii* | 5 | 43.926387 | -120.56049 | Previously published | Kierepka & Latch (2016) | KU764148.1 |
| OR_05 | - | - | *jeffersonii* | 86 | 43.926387 | -120.56049 | Previously published | Kierepka & Latch (2016) | KU764149.1 |
| OR_07 | - | - | *jeffersonii* | 51 | 43.926387 | -120.56049 | Previously published | Kierepka & Latch (2016) | KU764150.1 |
| OR_08 | - | - | *jeffersonii* | 86 | 43.926387 | -120.56049 | Previously published | Kierepka & Latch (2016) | KU764151.1 |
| OR_09 | - | - | *jeffersonii* | 80 | 43.926387 | -120.56049 | Previously published | Kierepka & Latch (2016) | KU764152.1 |
| OR_11 | - | - | *jeffersonii* | 80 | 43.926387 | -120.56049 | Previously published | Kierepka & Latch (2016) | KU764153.1 |
| OR_12 | - | - | *jeffersonii* | 1 | 43.926387 | -120.56049 | Previously published | Kierepka & Latch (2016) | KU764154.1 |
| OR_13 | - | - | *jeffersonii* | 1 | 43.926387 | -120.56049 | Previously published | Kierepka & Latch (2016) | KU764155.1 |
| OR_14 | - | - | *jeffersonii* | 80 | 43.926387 | -120.56049 | Previously published | Kierepka & Latch (2016) | KU764156.1 |
| OR_15 | - | - | *jeffersonii* | 30 | 43.926387 | -120.56049 | Previously published | Kierepka & Latch (2016) | KU764157.1 |
| OR_16 | - | - | *jeffersonii* | 33 | 43.926387 | -120.56049 | Previously published | Kierepka & Latch (2016) | KU764158.1 |
| SD_001 | - | - | *taxus* | 57 | 44.4383548 | -100.23214 | Previously published | Kierepka & Latch (2016) | KU764237.1 |
| SD03 | - | - | *taxus* | 3 | 44.4383548 | -100.23214 | Previously published | Kierepka & Latch (2016) | KU764160.1 |
| SD11 | - | - | *taxus* | 97 | 44.4383548 | -100.23214 | Previously published | Kierepka & Latch (2016) | KU764161.1 |
| SD118 | - | - | *taxus* | 3 | 44.4383548 | -100.23214 | Previously published | Kierepka & Latch (2016) | KU764162.1 |
| SD119 | - | - | *taxus* | 1 | 44.4383548 | -100.23214 | Previously published | Kierepka & Latch (2016) | KU764163.1 |
| SD13 | - | - | *taxus* | 80 | 44.4383548 | -100.23214 | Previously published | Kierepka & Latch (2016) | KU764169.1 |
| SD132 | - | - | *taxus* | 81 | 44.4383548 | -100.23214 | Previously published | Kierepka & Latch (2016) | KU764164.1 |
| SD133_10 | - | - | *taxus* | 69 | 44.4383548 | -100.23214 | Previously published | Kierepka & Latch (2016) | KU764165.1 |
| SD133_12 | - | - | *taxus* | 69 | 44.4383548 | -100.23214 | Previously published | Kierepka & Latch (2016) | KU764166.1 |
| SD135 | - | - | *taxus* | 3 | 44.4383548 | -100.23214 | Previously published | Kierepka & Latch (2016) | KU764167.1 |
| SD139 | - | - | *taxus* | 2 | 44.4383548 | -100.23214 | Previously published | Kierepka & Latch (2016) | KU764168.1 |
| SD141 | - | - | *taxus* | 1 | 44.4383548 | -100.23214 | Previously published | Kierepka & Latch (2016) | KU764170.1 |
| SD148 | - | - | *taxus* | 8 | 44.4383548 | -100.23214 | Previously published | Kierepka & Latch (2016) | KU764171.1 |
| SD167 | - | - | *taxus* | 99 | 44.4383548 | -100.23214 | Previously published | Kierepka & Latch (2016) | KU764172.1 |
| SD171 | - | - | *taxus* | 2 | 44.4383548 | -100.23214 | Previously published | Kierepka & Latch (2016) | KU764173.1 |
| SD175 | - | - | *taxus* | 2 | 44.4383548 | -100.23214 | Previously published | Kierepka & Latch (2016) | KU764174.1 |
| SD179 | - | - | *taxus* | 48 | 44.4383548 | -100.23214 | Previously published | Kierepka & Latch (2016) | KU764175.1 |
| SD181 | - | - | *taxus* | 73 | 44.4383548 | -100.23214 | Previously published | Kierepka & Latch (2016) | KU764176.1 |
| SD183 | - | - | *taxus* | 2 | 44.4383548 | -100.23214 | Previously published | Kierepka & Latch (2016) | KU764177.1 |
| SD185 | - | - | *taxus* | 79 | 44.4383548 | -100.23214 | Previously published | Kierepka & Latch (2016) | KU764178.1 |
| SD186 | - | - | *taxus* | 1 | 44.4383548 | -100.23214 | Previously published | Kierepka & Latch (2016) | KU764179.1 |
| SD187 | - | - | *taxus* | 1 | 44.4383548 | -100.23214 | Previously published | Kierepka & Latch (2016) | KU764180.1 |
| SD188 | - | - | *taxus* | 88 | 44.4383548 | -100.23214 | Previously published | Kierepka & Latch (2016) | KU764181.1 |
| SD189 | - | - | *taxus* | 99 | 44.4383548 | -100.23214 | Previously published | Kierepka & Latch (2016) | KU764182.1 |
| SD19 | - | - | *taxus* | 2 | 44.4383548 | -100.23214 | Previously published | Kierepka & Latch (2016) | KU764186.1 |
| SD191 | - | - | *taxus* | 75 | 44.4383548 | -100.23214 | Previously published | Kierepka & Latch (2016) | KU764183.1 |
| SD197 | - | - | *taxus* | 1 | 44.4383548 | -100.23214 | Previously published | Kierepka & Latch (2016) | KU764184.1 |
| SD198 | - | - | *taxus* | 64 | 44.4383548 | -100.23214 | Previously published | Kierepka & Latch (2016) | KU764185.1 |
| SD20 | - | - | *taxus* | 1 | 44.4383548 | -100.23214 | Previously published | Kierepka & Latch (2016) | KU764187.1 |
| SD200 | - | - | *taxus* | 16 | 44.4383548 | -100.23214 | Previously published | Kierepka & Latch (2016) | KU764188.1 |
| SD207 | - | - | *taxus* | 33 | 44.4383548 | -100.23214 | Previously published | Kierepka & Latch (2016) | KU764189.1 |
| SD222 | - | - | *taxus* | 3 | 44.4383548 | -100.23214 | Previously published | Kierepka & Latch (2016) | KU764190.1 |
| SD227 | - | - | *taxus* | 99 | 44.4383548 | -100.23214 | Previously published | Kierepka & Latch (2016) | KU764191.1 |
| SD228 | - | - | *taxus* | 1 | 44.4383548 | -100.23214 | Previously published | Kierepka & Latch (2016) | KU764192.1 |
| SD237_10 | - | - | *taxus* | 1 | 44.4383548 | -100.23214 | Previously published | Kierepka & Latch (2016) | KU764193.1 |
| SD237_12 | - | - | *taxus* | 1 | 44.4383548 | -100.23214 | Previously published | Kierepka & Latch (2016) | KU764194.1 |
| SD238 | - | - | *taxus* | 67 | 44.4383548 | -100.23214 | Previously published | Kierepka & Latch (2016) | KU764195.1 |
| SD239 | - | - | *taxus* | 27 | 44.4383548 | -100.23214 | Previously published | Kierepka & Latch (2016) | KU764196.1 |
| SD241 | - | - | *taxus* | 99 | 44.4383548 | -100.23214 | Previously published | Kierepka & Latch (2016) | KU764197.1 |
| SD25 | - | - | *taxus* | 48 | 44.4383548 | -100.23214 | Previously published | Kierepka & Latch (2016) | KU764198.1 |
| SD253 | - | - | *taxus* | 2 | 44.4383548 | -100.23214 | Previously published | Kierepka & Latch (2016) | KU764199.1 |
| SD258 | - | - | *taxus* | 18 | 44.4383548 | -100.23214 | Previously published | Kierepka & Latch (2016) | KU764200.1 |
| SD260 | - | - | *taxus* | 37 | 44.4383548 | -100.23214 | Previously published | Kierepka & Latch (2016) | KU764201.1 |
| SD265 | - | - | *taxus* | 1 | 44.4383548 | -100.23214 | Previously published | Kierepka & Latch (2016) | KU764202.1 |
| SD269 | - | - | *taxus* | 99 | 44.4383548 | -100.23214 | Previously published | Kierepka & Latch (2016) | KU764203.1 |
| SD275 | - | - | *taxus* | 2 | 44.4383548 | -100.23214 | Previously published | Kierepka & Latch (2016) | KU764204.1 |
| SD277 | - | - | *taxus* | 99 | 44.4383548 | -100.23214 | Previously published | Kierepka & Latch (2016) | KU764205.1 |
| SD278 | - | - | *taxus* | 2 | 44.4383548 | -100.23214 | Previously published | Kierepka & Latch (2016) | KU764206.1 |
| SD279 | - | - | *taxus* | 2 | 44.4383548 | -100.23214 | Previously published | Kierepka & Latch (2016) | KU764207.1 |
| SD280 | - | - | *taxus* | 48 | 44.4383548 | -100.23214 | Previously published | Kierepka & Latch (2016) | KU764208.1 |
| SD283 | - | - | *taxus* | 99 | 44.4383548 | -100.23214 | Previously published | Kierepka & Latch (2016) | KU764209.1 |
| SD284 | - | - | *taxus* | 1 | 44.4383548 | -100.23214 | Previously published | Kierepka & Latch (2016) | KU764210.1 |
| SD285 | - | - | *taxus* | 48 | 44.4383548 | -100.23214 | Previously published | Kierepka & Latch (2016) | KU764211.1 |
| SD286 | - | - | *taxus* | 2 | 44.4383548 | -100.23214 | Previously published | Kierepka & Latch (2016) | KU764212.1 |
| SD290 | - | - | *taxus* | 1 | 44.4383548 | -100.23214 | Previously published | Kierepka & Latch (2016) | KU764213.1 |
| SD292 | - | - | *taxus* | 99 | 44.4383548 | -100.23214 | Previously published | Kierepka & Latch (2016) | KU764214.1 |
| SD293 | - | - | *taxus* | 99 | 44.4383548 | -100.23214 | Previously published | Kierepka & Latch (2016) | KU764215.1 |
| SD299 | - | - | *taxus* | 2 | 44.4383548 | -100.23214 | Previously published | Kierepka & Latch (2016) | KU764216.1 |
| SD300 | - | - | *taxus* | 48 | 44.4383548 | -100.23214 | Previously published | Kierepka & Latch (2016) | KU764217.1 |
| SD347 | - | - | *taxus* | 3 | 44.4383548 | -100.23214 | Previously published | Kierepka & Latch (2016) | KU764218.1 |
| SD349 | - | - | *taxus* | 48 | 44.4383548 | -100.23214 | Previously published | Kierepka & Latch (2016) | KU764219.1 |
| SD35 | - | - | *taxus* | 2 | 44.4383548 | -100.23214 | Previously published | Kierepka & Latch (2016) | KU764220.1 |
| SD350 | - | - | *taxus* | 2 | 44.4383548 | -100.23214 | Previously published | Kierepka & Latch (2016) | KU764221.1 |
| SD352 | - | - | *taxus* | 1 | 44.4383548 | -100.23214 | Previously published | Kierepka & Latch (2016) | KU764222.1 |
| SD354 | - | - | *taxus* | 26 | 44.4383548 | -100.23214 | Previously published | Kierepka & Latch (2016) | KU764223.1 |
| SD355 | - | - | *taxus* | 2 | 44.4383548 | -100.23214 | Previously published | Kierepka & Latch (2016) | KU764224.1 |
| SD356 | - | - | *taxus* | 80 | 44.4383548 | -100.23214 | Previously published | Kierepka & Latch (2016) | KU764225.1 |
| SD361 | - | - | *taxus* | 3 | 44.4383548 | -100.23214 | Previously published | Kierepka & Latch (2016) | KU764226.1 |
| SD363 | - | - | *taxus* | 2 | 44.4383548 | -100.23214 | Previously published | Kierepka & Latch (2016) | KU764227.1 |
| SD367 | - | - | *taxus* | 2 | 44.4383548 | -100.23214 | Previously published | Kierepka & Latch (2016) | KU764228.1 |
| SD41 | - | - | *taxus* | 1 | 44.4383548 | -100.23214 | Previously published | Kierepka & Latch (2016) | KU764229.1 |
| SD48 | - | - | *taxus* | 89 | 44.4383548 | -100.23214 | Previously published | Kierepka & Latch (2016) | KU764230.1 |
| SD59 | - | - | *taxus* | 1 | 44.4383548 | -100.23214 | Previously published | Kierepka & Latch (2016) | KU764231.1 |
| SD61 | - | - | *taxus* | 1 | 44.4383548 | -100.23214 | Previously published | Kierepka & Latch (2016) | KU764232.1 |
| SD62 | - | - | *taxus* | 2 | 44.4383548 | -100.23214 | Previously published | Kierepka & Latch (2016) | KU764233.1 |
| SD69 | - | - | *taxus* | 1 | 44.4383548 | -100.23214 | Previously published | Kierepka & Latch (2016) | KU764234.1 |
| SD91 | - | - | *taxus* | 2 | 44.4383548 | -100.23214 | Previously published | Kierepka & Latch (2016) | KU764235.1 |
| SD96 | - | - | *taxus* | 97 | 44.4383548 | -100.23214 | Previously published | Kierepka & Latch (2016) | KU764236.1 |
| SK_4ILT387 | - | - | *taxus* | 2 | 49.923218 | -107.33568 | Previously published | Ethier et al. (2012) | GU901616.1 |
| SK_4ILT388 | - | - | *taxus* | 2 | 49.923218 | -107.33568 | Previously published | Ethier et al. (2012) | GU901615.1 |
| SK_4ILT557 | - | - | *taxus* | 2 | 49.923218 | -107.33568 | Previously published | Ethier et al. (2012) | GU901614.1 |
| SK_4ILT558 | - | - | *taxus* | 2 | 49.923218 | -107.33568 | Previously published | Ethier et al. (2012) | GU901613.1 |
| SK_4ILX227 | - | - | *taxus* | 12 | 49.923218 | -107.33568 | Previously published | Ethier et al. (2012) | GU901612.1 |
| SK_4IMV603 | - | - | *taxus* | 2 | 49.923218 | -107.33568 | Previously published | Ethier et al. (2012) | GU901611.1 |
| SK_4IMV604 | - | - | *taxus* | 2 | 49.923218 | -107.33568 | Previously published | Ethier et al. (2012) | GU901610.1 |
| SK_4INE275 | - | - | *taxus* | 31 | 49.923218 | -107.33568 | Previously published | Ethier et al. (2012) | GU901609.1 |
| SK_4IQU589 | - | - | *taxus* | 2 | 49.923218 | -107.33568 | Previously published | Ethier et al. (2012) | GU901617.1 |
| SK_4IQU719 | - | - | *taxus* | 2 | 49.923218 | -107.33568 | Previously published | Ethier et al. (2012) | GU901618.1 |
| SK_4IQU720 | - | - | *taxus* | 73 | 49.923218 | -107.33568 | Previously published | Ethier et al. (2012) | GU901607.1 |
| SK_4IQU721 | - | - | *taxus* | 2 | 49.923218 | -107.33568 | Previously published | Ethier et al. (2012) | GU901619.1 |
| SK_4IQU722 | - | - | *taxus* | 110 | 49.923218 | -107.33568 | Previously published | Ethier et al. (2012) | GU901606.1 |
| SK_4IQU723 | - | - | *taxus* | 2 | 49.923218 | -107.33568 | Previously published | Ethier et al. (2012) | GU901605.1 |
| SK_4IQU724 | - | - | *taxus* | 31 | 49.923218 | -107.33568 | Previously published | Ethier et al. (2012) | GU901604.1 |
| SK_4IQU725 | - | - | *taxus* | 2 | 49.923218 | -107.33568 | Previously published | Ethier et al. (2012) | GU901603.1 |
| SK_4IQU726 | - | - | *taxus* | 2 | 49.923218 | -107.33568 | Previously published | Ethier et al. (2012) | GU901602.1 |
| SK_4IQU727 | - | - | *taxus* | 111 | 49.923218 | -107.33568 | Previously published | Ethier et al. (2012) | GU901608.1 |
| SK_4IQU728 | - | - | *taxus* | 2 | 49.923218 | -107.33568 | Previously published | Ethier et al. (2012) | GU901601.1 |
| SK_4IQU730 | - | - | *taxus* | 2 | 49.923218 | -107.33568 | Previously published | Ethier et al. (2012) | GU901600.1 |
| SK_4IQU734 | - | - | *taxus* | 111 | 49.923218 | -107.33568 | Previously published | Ethier et al. (2012) | GU901599.1 |
| SK_4IQU890 | - | - | *taxus* | 2 | 49.923218 | -107.33568 | Previously published | Ethier et al. (2012) | GU901598.1 |
| SK_4IQV075 | - | - | *taxus* | 2 | 49.923218 | -107.33568 | Previously published | Ethier et al. (2012) | GU901620.1 |
| SK_4IQV076 | - | - | *taxus* | 44 | 49.923218 | -107.33568 | Previously published | Ethier et al. (2012) | GU901597.1 |
| SK_4ISE271 | - | - | *taxus* | 39 | 49.923218 | -107.33568 | Previously published | Ethier et al. (2012) | GU901621.1 |
| SK_SK12_07A | - | - | *taxus* | 12 | 49.923218 | -107.33568 | Previously published | Ethier et al. (2012) | GU901596.1 |
| SK_SK12_07B | - | - | *taxus* | 12 | 49.923218 | -107.33568 | Previously published | Ethier et al. (2012) | GU901595.1 |
| SK06 | - | - | *taxus* | 2 | 50.898247 | -106.03946 | Previously published | Kierepka & Latch (2016) | KU763590.1 |
| SK160 | - | - | *taxus* | 2 | 50.898247 | -106.03946 | Previously published | Kierepka & Latch (2016) | KU763591.1 |
| SK161 | - | - | *taxus* | 2 | 50.898247 | -106.03946 | Previously published | Kierepka & Latch (2016) | KU763592.1 |
| SK211 | - | - | *taxus* | 86 | 50.898247 | -106.03946 | Previously published | Kierepka & Latch (2016) | KU763593.1 |
| SK212 | - | - | *taxus* | 1 | 50.898247 | -106.03946 | Previously published | Kierepka & Latch (2016) | KU763594.1 |
| SK214 | - | - | *taxus* | 2 | 50.898247 | -106.03946 | Previously published | Kierepka & Latch (2016) | KU763595.1 |
| SK217 | - | - | *taxus* | 2 | 50.898247 | -106.03946 | Previously published | Kierepka & Latch (2016) | KU763596.1 |
| SK218 | - | - | *taxus* | 31 | 50.898247 | -106.03946 | Previously published | Kierepka & Latch (2016) | KU763597.1 |
| SK219 | - | - | *taxus* | 2 | 50.898247 | -106.03946 | Previously published | Kierepka & Latch (2016) | KU763598.1 |
| SK220 | - | - | *taxus* | 2 | 50.898247 | -106.03946 | Previously published | Kierepka & Latch (2016) | KU763599.1 |
| SK221 | - | - | *taxus* | 44 | 50.898247 | -106.03946 | Previously published | Kierepka & Latch (2016) | KU763600.1 |
| SK225 | - | - | *taxus* | 28 | 50.898247 | -106.03946 | Previously published | Kierepka & Latch (2016) | KU763601.1 |
| SK227 | - | - | *taxus* | 2 | 50.898247 | -106.03946 | Previously published | Kierepka & Latch (2016) | KU763602.1 |
| SK228 | - | - | *taxus* | 1 | 50.898247 | -106.03946 | Previously published | Kierepka & Latch (2016) | KU763603.1 |
| SK229 | - | - | *taxus* | 2 | 50.898247 | -106.03946 | Previously published | Kierepka & Latch (2016) | KU763604.1 |
| SK231 | - | - | *taxus* | 2 | 50.898247 | -106.03946 | Previously published | Kierepka & Latch (2016) | KU763605.1 |
| SK234 | - | - | *taxus* | 2 | 50.898247 | -106.03946 | Previously published | Kierepka & Latch (2016) | KU763606.1 |
| SK244 | - | - | *taxus* | 2 | 50.898247 | -106.03946 | Previously published | Kierepka & Latch (2016) | KU763607.1 |
| SK247 | - | - | *taxus* | 2 | 50.898247 | -106.03946 | Previously published | Kierepka & Latch (2016) | KU763608.1 |
| SK248 | - | - | *taxus* | 1 | 50.898247 | -106.03946 | Previously published | Kierepka & Latch (2016) | KU763609.1 |
| SK250 | - | - | *taxus* | 48 | 50.898247 | -106.03946 | Previously published | Kierepka & Latch (2016) | KU763610.1 |
| SK52 | - | - | *taxus* | 2 | 50.898247 | -106.03946 | Previously published | Kierepka & Latch (2016) | KU763611.1 |
| SK54 | - | - | *taxus* | 2 | 50.898247 | -106.03946 | Previously published | Kierepka & Latch (2016) | KU763612.1 |
| SK72 | - | - | *taxus* | 44 | 50.898247 | -106.03946 | Previously published | Kierepka & Latch (2016) | KU763613.1 |
| SK75 | - | - | *taxus* | 2 | 50.898247 | -106.03946 | Previously published | Kierepka & Latch (2016) | KU763614.1 |
| SK76 | - | - | *taxus* | 2 | 50.898247 | -106.03946 | Previously published | Kierepka & Latch (2016) | KU763615.1 |
| TH01_3229 | - | - | *jeffersonii* | 4 | 50.4544977 | -119.44278 | Previously published | Ford et al. (2019) | MK882796.1 |
| TH01_3231 | - | - | *jeffersonii* | 4 | 51.6400159 | -121.31159 | Previously published | Ford et al. (2019) | MK882797.1 |
| TH01_3232 | - | - | *jeffersonii* | 4 | 50.6497438 | -120.00675 | Previously published | Ford et al. (2019) | MK882798.1 |
| TH10_3230 | - | - | *jeffersonii* | 4 | 50.6584273 | -120.39213 | Previously published | Ford et al. (2019) | MK882799.1 |
| TH12_2981 | - | - | *jeffersonii* | 4 | 50.658427 | -120.39213 | Previously published | Ford et al. (2019) | MK882800.1 |
| TH12_2986 | - | - | *jeffersonii* | 2 | 50.6688 | -120.35788 | Previously published | Ford et al. (2019) | MK882801.1 |
| TH15_6146 | - | - | *jeffersonii* | 2 | 50.8128156 | -120.29434 | Previously published | Ford et al. (2019) | MK882802.1 |
| TH15_6203 | - | - | *jeffersonii* | 4 | 50.815066 | -120.29249 | Previously published | Ford et al. (2019) | MK882803.1 |
| TH15_6250 | - | - | *jeffersonii* | 4 | 51.0427887 | -120.72954 | Previously published | Ford et al. (2019) | MK882804.1 |
| TH15_6293 | - | - | *jeffersonii* | 4 | 50.7445475 | -119.75469 | Previously published | Ford et al. (2019) | MK882805.1 |
| TH15_6299 | - | - | *jeffersonii* | 2 | 51.0998094 | -120.14438 | Previously published | Ford et al. (2019) | MK882806.1 |
| TH15_7396 | - | - | *jeffersonii* | 2 | 50.694599 | -119.80886 | Previously published | Ford et al. (2019) | MK882807.1 |
| TH15_7399 | - | - | *jeffersonii* | 4 | 50.755158 | -120.6196 | Previously published | Ford et al. (2019) | MK882808.1 |
| TH15_7402 | - | - | *jeffersonii* | 44 | 50.805374 | -120.39818 | Previously published | Ford et al. (2019) | MK882809.1 |
| TH15_7403 | - | - | *jeffersonii* | 2 | 51.148931 | -120.11661 | Previously published | Ford et al. (2019) | MK882810.1 |
| TH15_7405 | - | - | *jeffersonii* | 4 | 50.47201 | -119.77148 | Previously published | Ford et al. (2019) | MK882811.1 |
| TH15_7415 | - | - | *jeffersonii* | 4 | 50.716267 | -119.78195 | Previously published | Ford et al. (2019) | MK882812.1 |
| UP_0225 | - | - | *taxus* | 1 | 46.3051266 | -87.359296 | Previously published | Kierepka & Latch (2016) | KU764238.1 |
| UP_0226 | - | - | *taxus* | 1 | 46.3051266 | -87.359296 | Previously published | Kierepka & Latch (2016) | KU764239.1 |
| UP_0240 | - | - | *taxus* | 1 | 46.3051266 | -87.359296 | Previously published | Kierepka & Latch (2016) | KU764240.1 |
| UP_0241 | - | - | *taxus* | 1 | 46.3051266 | -87.359296 | Previously published | Kierepka & Latch (2016) | KU764241.1 |
| UP_0247 | - | - | *taxus* | 1 | 46.3051266 | -87.359296 | Previously published | Kierepka & Latch (2016) | KU764242.1 |
| UP_0248 | - | - | *taxus* | 1 | 46.3051266 | -87.359296 | Previously published | Kierepka & Latch (2016) | KU764243.1 |
| UP_0249 | - | - | *taxus* | 3 | 46.3051266 | -87.359296 | Previously published | Kierepka & Latch (2016) | KU764244.1 |
| UP_025 | - | - | *taxus* | 1 | 46.3051266 | -87.359296 | Previously published | Kierepka & Latch (2016) | KU764245.1 |
| UP_0250 | - | - | *taxus* | 1 | 46.3051266 | -87.359296 | Previously published | Kierepka & Latch (2016) | KU764246.1 |
| UP_0261 | - | - | *taxus* | 1 | 46.3051266 | -87.359296 | Previously published | Kierepka & Latch (2016) | KU764247.1 |
| UP_0275 | - | - | *taxus* | 2 | 46.3051266 | -87.359296 | Previously published | Kierepka & Latch (2016) | KU764248.1 |
| UP_0276 | - | - | *taxus* | 1 | 46.3051266 | -87.359296 | Previously published | Kierepka & Latch (2016) | KU764249.1 |
| UP_0277 | - | - | *taxus* | 1 | 46.3051266 | -87.359296 | Previously published | Kierepka & Latch (2016) | KU764250.1 |
| UP_0278 | - | - | *taxus* | 2 | 46.3051266 | -87.359296 | Previously published | Kierepka & Latch (2016) | KU764251.1 |
| UP_0279 | - | - | *taxus* | 1 | 46.3051266 | -87.359296 | Previously published | Kierepka & Latch (2016) | KU764252.1 |
| UP_0280 | - | - | *taxus* | 1 | 46.3051266 | -87.359296 | Previously published | Kierepka & Latch (2016) | KU764253.1 |
| UP_0281 | - | - | *taxus* | 1 | 46.3051266 | -87.359296 | Previously published | Kierepka & Latch (2016) | KU764254.1 |
| UP_0285 | - | - | *taxus* | 1 | 46.3051266 | -87.359296 | Previously published | Kierepka & Latch (2016) | KU764255.1 |
| UP_UP0173 | - | - | *taxus* | 12 | 46.329041 | -85.989556 | Previously published | Ethier et al. (2012) | GU901569.1 |
| UP_UP02_26 | - | - | *taxus* | 12 | 46.329041 | -85.989556 | Previously published | Ethier et al. (2012) | GU901561.1 |
| UP_UP02_78 | - | - | *taxus* | 2 | 46.329041 | -85.989556 | Previously published | Ethier et al. (2012) | GU901560.1 |
| UP_UP0205 | - | - | *taxus* | 12 | 46.329041 | -85.989556 | Previously published | Ethier et al. (2012) | GU901563.1 |
| UP_UP0215 | - | - | *taxus* | 12 | 46.329041 | -85.989556 | Previously published | Ethier et al. (2012) | GU901564.1 |
| UP_UP0218 | - | - | *taxus* | 2 | 46.329041 | -85.989556 | Previously published | Ethier et al. (2012) | GU901566.1 |
| UP_UP0225 | - | - | *taxus* | 12 | 46.329041 | -85.989556 | Previously published | Ethier et al. (2012) | GU901574.1 |
| UP_UP0240 | - | - | *taxus* | 12 | 46.329041 | -85.989556 | Previously published | Ethier et al. (2012) | GU901562.1 |
| UP_UP0241 | - | - | *taxus* | 12 | 46.329041 | -85.989556 | Previously published | Ethier et al. (2012) | GU901570.1 |
| UP_UP0247 | - | - | *taxus* | 12 | 46.329041 | -85.989556 | Previously published | Ethier et al. (2012) | GU901559.1 |
| UP_UP0261 | - | - | *taxus* | 12 | 46.329041 | -85.989556 | Previously published | Ethier et al. (2012) | GU901567.1 |
| UP_UP0275 | - | - | *taxus* | 2 | 46.329041 | -85.989556 | Previously published | Ethier et al. (2012) | GU901565.1 |
| UP_UP0276 | - | - | *taxus* | 12 | 46.329041 | -85.989556 | Previously published | Ethier et al. (2012) | GU901573.1 |
| UP_UP0277 | - | - | *taxus* | 12 | 46.329041 | -85.989556 | Previously published | Ethier et al. (2012) | GU901572.1 |
| UP_UP0279 | - | - | *taxus* | 12 | 46.329041 | -85.989556 | Previously published | Ethier et al. (2012) | GU901568.1 |
| UP_UP0281 | - | - | *taxus* | 12 | 46.329041 | -85.989556 | Previously published | Ethier et al. (2012) | GU901575.1 |
| UP_UP0289 | - | - | *taxus* | 12 | 46.329041 | -85.989556 | Previously published | Ethier et al. (2012) | GU901571.1 |
| UT_T1 | - | - | *jeffersonii* | 19 | 39.304345 | -111.66817 | Previously published | Kierepka & Latch (2016) | KU764257.1 |
| UT_T10 | - | - | *jeffersonii* | 33 | 39.304345 | -111.66817 | Previously published | Kierepka & Latch (2016) | KU764258.1 |
| UT_T12 | - | - | *jeffersonii* | 33 | 39.304345 | -111.66817 | Previously published | Kierepka & Latch (2016) | KU764259.1 |
| UT_T13 | - | - | *jeffersonii* | 58 | 39.304345 | -111.66817 | Previously published | Kierepka & Latch (2016) | KU764260.1 |
| UT_T14 | - | - | *jeffersonii* | 52 | 39.304345 | -111.66817 | Previously published | Kierepka & Latch (2016) | KU764261.1 |
| UT_T15 | - | - | *jeffersonii* | 95 | 39.304345 | -111.66817 | Previously published | Kierepka & Latch (2016) | KU764262.1 |
| UT_T17 | - | - | *jeffersonii* | 80 | 39.304345 | -111.66817 | Previously published | Kierepka & Latch (2016) | KU764263.1 |
| UT_T20 | - | - | *jeffersonii* | 2 | 39.304345 | -111.66817 | Previously published | Kierepka & Latch (2016) | KU764264.1 |
| UT_T21 | - | - | *jeffersonii* | 95 | 39.304345 | -111.66817 | Previously published | Kierepka & Latch (2016) | KU764265.1 |
| UT_T22 | - | - | *jeffersonii* | 80 | 39.304345 | -111.66817 | Previously published | Kierepka & Latch (2016) | KU764266.1 |
| UT_T24 | - | - | *jeffersonii* | 96 | 39.304345 | -111.66817 | Previously published | Kierepka & Latch (2016) | KU764267.1 |
| UT_T26 | - | - | *jeffersonii* | 91 | 39.304345 | -111.66817 | Previously published | Kierepka & Latch (2016) | KU764268.1 |
| UT_T27 | - | - | *jeffersonii* | 80 | 39.304345 | -111.66817 | Previously published | Kierepka & Latch (2016) | KU764269.1 |
| UT_T28 | - | - | *jeffersonii* | 80 | 39.304345 | -111.66817 | Previously published | Kierepka & Latch (2016) | KU764270.1 |
| UT_T29 | - | - | *jeffersonii* | 95 | 39.304345 | -111.66817 | Previously published | Kierepka & Latch (2016) | KU764271.1 |
| UT_T3 | - | - | *jeffersonii* | 33 | 39.304345 | -111.66817 | Previously published | Kierepka & Latch (2016) | KU764272.1 |
| UT_T30 | - | - | *jeffersonii* | 96 | 39.304345 | -111.66817 | Previously published | Kierepka & Latch (2016) | KU764273.1 |
| UT_T31 | - | - | *jeffersonii* | 80 | 39.304345 | -111.66817 | Previously published | Kierepka & Latch (2016) | KU764274.1 |
| UT_T32 | - | - | *jeffersonii* | 80 | 39.304345 | -111.66817 | Previously published | Kierepka & Latch (2016) | KU764275.1 |
| UT_T33 | - | - | *jeffersonii* | 17 | 39.304345 | -111.66817 | Previously published | Kierepka & Latch (2016) | KU764276.1 |
| UT_T34 | - | - | *jeffersonii* | 80 | 39.304345 | -111.66817 | Previously published | Kierepka & Latch (2016) | KU764277.1 |
| UT_T35 | - | - | *jeffersonii* | 21 | 39.304345 | -111.66817 | Previously published | Kierepka & Latch (2016) | KU764278.1 |
| UT_T36 | - | - | *jeffersonii* | 96 | 39.304345 | -111.66817 | Previously published | Kierepka & Latch (2016) | KU764279.1 |
| UT_T37 | - | - | *jeffersonii* | 96 | 39.304345 | -111.66817 | Previously published | Kierepka & Latch (2016) | KU764280.1 |
| UT_T39 | - | - | *jeffersonii* | 33 | 39.304345 | -111.66817 | Previously published | Kierepka & Latch (2016) | KU764281.1 |
| UT_T4 | - | - | *jeffersonii* | 33 | 39.304345 | -111.66817 | Previously published | Kierepka & Latch (2016) | KU764282.1 |
| UT_T40 | - | - | *jeffersonii* | 96 | 39.304345 | -111.66817 | Previously published | Kierepka & Latch (2016) | KU764283.1 |
| UT_T41 | - | - | *jeffersonii* | 33 | 39.304345 | -111.66817 | Previously published | Kierepka & Latch (2016) | KU764284.1 |
| UT_T42 | - | - | *jeffersonii* | 96 | 39.304345 | -111.66817 | Previously published | Kierepka & Latch (2016) | KU764285.1 |
| UT_T44 | - | - | *jeffersonii* | 91 | 39.304345 | -111.66817 | Previously published | Kierepka & Latch (2016) | KU764286.1 |
| UT_T45 | - | - | *jeffersonii* | 2 | 39.304345 | -111.66817 | Previously published | Kierepka & Latch (2016) | KU764287.1 |
| UT_T46 | - | - | *jeffersonii* | 95 | 39.304345 | -111.66817 | Previously published | Kierepka & Latch (2016) | KU764288.1 |
| UT_T47 | - | - | *jeffersonii* | 62 | 39.304345 | -111.66817 | Previously published | Kierepka & Latch (2016) | KU764289.1 |
| UT_T48 | - | - | *jeffersonii* | 33 | 39.304345 | -111.66817 | Previously published | Kierepka & Latch (2016) | KU764290.1 |
| UT_T49 | - | - | *jeffersonii* | 96 | 39.304345 | -111.66817 | Previously published | Kierepka & Latch (2016) | KU764291.1 |
| UT_T5 | - | - | *jeffersonii* | 96 | 39.304345 | -111.66817 | Previously published | Kierepka & Latch (2016) | KU764292.1 |
| UT_T50 | - | - | *jeffersonii* | 6 | 39.304345 | -111.66817 | Previously published | Kierepka & Latch (2016) | KU764293.1 |
| UT_T6 | - | - | *jeffersonii* | 33 | 39.304345 | -111.66817 | Previously published | Kierepka & Latch (2016) | KU764294.1 |
| UT_T7 | - | - | *jeffersonii* | 20 | 39.304345 | -111.66817 | Previously published | Kierepka & Latch (2016) | KU764295.1 |
| UT_T8 | - | - | *jeffersonii* | 54 | 39.304345 | -111.66817 | Previously published | Kierepka & Latch (2016) | KU764296.1 |
| UT_T9 | - | - | *jeffersonii* | 91 | 39.304345 | -111.66817 | Previously published | Kierepka & Latch (2016) | KU764297.1 |
| UT5 | - | - | *jeffersonii* | 91 | 39.304345 | -111.66817 | Previously published | Kierepka & Latch (2016) | KU764256.1 |
| WA001 | - | - | *jeffersonii* | 2 | 47.3458912 | -119.27676 | Previously published | Kierepka & Latch (2016) | Direct from authors |
| WA1208 | - | - | *jeffersonii* | 4 | 48.482087 | -119.02918 | Previously published | Ford et al. (2019) | MK882813.1 |
| WA1209 | - | - | *jeffersonii* | 115 | 47.9738 | -119.10982 | Previously published | Ford et al. (2019) | MK882814.1 |
| WA1210 | - | - | *jeffersonii* | 58 | 48.76859 | -118.97955 | Previously published | Ford et al. (2019) | MK882815.1 |
| WA13_222 | - | - | *jeffersonii* | 4 | 46.43 | -117.53 | Previously published | Ford et al. (2019) | MK882816.1 |
| WA15_6787 | - | - | *jeffersonii* | 52 | 46.4207032 | -117.5068 | Previously published | Ford et al. (2019) | MK882817.1 |
| WA15_6788 | - | - | *jeffersonii* | 93 | 47.5579055 | -117.61096 | Previously published | Ford et al. (2019) | MK882818.1 |
| WA15_6792 | - | - | *jeffersonii* | 114 | 46.4673629 | -117.49734 | Previously published | Ford et al. (2019) | MK882819.1 |
| WA15_6793 | - | - | *jeffersonii* | 114 | 47.0581823 | -117.63927 | Previously published | Ford et al. (2019) | MK882820.1 |
| WA15_6795 | - | - | *jeffersonii* | 44 | 47.4328079 | -117.79417 | Previously published | Ford et al. (2019) | MK882821.1 |
| WA15_6797 | - | - | *jeffersonii* | 2 | 47.6393629 | -117.49321 | Previously published | Ford et al. (2019) | MK882822.1 |
| WA15_6798 | - | - | *jeffersonii* | 58 | 47.5857277 | -117.64853 | Previously published | Ford et al. (2019) | MK882823.1 |
| WA15_6799 | - | - | *jeffersonii* | 30 | 46.0960907 | -118.19413 | Previously published | Ford et al. (2019) | MK882824.1 |
| WA15_7362 | - | - | *jeffersonii* | 4 | 47.65787 | -118.00774 | Previously published | Ford et al. (2019) | MK882825.1 |
| WA15_7375 | - | - | *jeffersonii* | 4 | 47.648592 | -120.05262 | Previously published | Ford et al. (2019) | MK882826.1 |
| WA15_7377 | - | - | *jeffersonii* | 58 | 47.657766 | -117.98456 | Previously published | Ford et al. (2019) | MK882827.1 |
| WA15_7378 | - | - | *jeffersonii* | 58 | 46.868182 | -119.19505 | Previously published | Ford et al. (2019) | MK882828.1 |
| WA15_7379 | - | - | *jeffersonii* | 58 | 47.08719 | -118.87493 | Previously published | Ford et al. (2019) | MK882829.1 |
| WA15_7380 | - | - | *jeffersonii* | 52 | 46.946508 | -120.19991 | Previously published | Ford et al. (2019) | MK882830.1 |
| WA15_7381 | - | - | *jeffersonii* | 58 | 46.83869 | -120.372 | Previously published | Ford et al. (2019) | MK882831.1 |
| WA15_7382 | - | - | *jeffersonii* | 58 | 47.581944 | -118.46103 | Previously published | Ford et al. (2019) | MK882832.1 |
| WA15_7384 | - | - | *jeffersonii* | 4 | 47.566585 | -118.48896 | Previously published | Ford et al. (2019) | MK882833.1 |
| WA15_7386 | - | - | *jeffersonii* | 58 | 47.610205 | -118.53425 | Previously published | Ford et al. (2019) | MK882834.1 |
| WA15_7409 | - | - | *jeffersonii* | 4 | 48.463407 | -119.18215 | Previously published | Ford et al. (2019) | MK882835.1 |
| WA16_8444 | - | - | *jeffersonii* | 2 | 47.1062989 | -120.76562 | Previously published | Ford et al. (2019) | MK882836.1 |
| WA16_8452 | - | - | *jeffersonii* | 75 | 47.393713 | -119.43358 | Previously published | Ford et al. (2019) | MK882837.1 |
| WA16_8453 | - | - | *jeffersonii* | 58 | 47.975913 | -119.14185 | Previously published | Ford et al. (2019) | MK882838.1 |
| WA16_8457 | - | - | *jeffersonii* | 58 | 48.89629 | -119.07589 | Previously published | Ford et al. (2019) | MK882839.1 |
| WA16_8458 | - | - | *jeffersonii* | 2 | 48.543175 | -119.11326 | Previously published | Ford et al. (2019) | MK882840.1 |
| WA16_8459 | - | - | *jeffersonii* | 58 | 48.9564078 | -119.05194 | Previously published | Ford et al. (2019) | MK882841.1 |
| WA16_8463 | - | - | *jeffersonii* | 115 | 48.647415 | -119.54227 | Previously published | Ford et al. (2019) | MK882842.1 |
| WA16_8466 | - | - | *jeffersonii* | 4 | 48.58591 | -118.14075 | Previously published | Ford et al. (2019) | MK882843.1 |
| WA16_8467 | - | - | *jeffersonii* | 4 | 47.595043 | -118.73588 | Previously published | Ford et al. (2019) | MK882844.1 |
| WA16_8469 | - | - | *jeffersonii* | 2 | 46.885118 | -120.42617 | Previously published | Ford et al. (2019) | MK882845.1 |
| WA16_8470 | - | - | *jeffersonii* | 4 | 47.312031 | -117.96411 | Previously published | Ford et al. (2019) | MK882846.1 |
| WA16_8471 | - | - | *jeffersonii* | 4 | 46.297497 | -118.65116 | Previously published | Ford et al. (2019) | MK882847.1 |
| WA16_8475 | - | - | *jeffersonii* | 58 | 47.976078 | -119.18179 | Previously published | Ford et al. (2019) | MK882848.1 |
| WA200914 | - | - | *jeffersonii* | 4 | 48.55 | -119.75 | Previously published | Ford et al. (2019) | MK882849.1 |
| WA34282 | - | - | *jeffersonii* | 58 | 46.549761 | -118.92352 | Previously published | Ford et al. (2019) | MK882850.1 |
| WA82298 | - | - | *jeffersonii* | 58 | 47.331771 | -119.54323 | Previously published | Ford et al. (2019) | MK882852.1 |
| WI_105 | - | - | *taxus* | 2 | 44.6179495 | -89.989755 | Previously published | Kierepka & Latch (2016) | KU764298.1 |
| WI_Ad01 | - | - | *taxus* | 1 | 44.6179495 | -89.989755 | Previously published | Kierepka & Latch (2016) | KU764299.1 |
| WI_Ad02 | - | - | *taxus* | 1 | 44.6179495 | -89.989755 | Previously published | Kierepka & Latch (2016) | KU764300.1 |
| WI_As01 | - | - | *taxus* | 1 | 44.6179495 | -89.989755 | Previously published | Kierepka & Latch (2016) | KU764301.1 |
| WI_Ba06 | - | - | *taxus* | 1 | 44.6179495 | -89.989755 | Previously published | Kierepka & Latch (2016) | KU764302.1 |
| WI_Ba16 | - | - | *taxus* | 1 | 44.6179495 | -89.989755 | Previously published | Kierepka & Latch (2016) | KU764303.1 |
| WI_Ba17 | - | - | *taxus* | 1 | 44.6179495 | -89.989755 | Previously published | Kierepka & Latch (2016) | KU764304.1 |
| WI_Ba18 | - | - | *taxus* | 37 | 44.6179495 | -89.989755 | Previously published | Kierepka & Latch (2016) | KU764305.1 |
| WI_Ba19 | - | - | *taxus* | 1 | 44.6179495 | -89.989755 | Previously published | Kierepka & Latch (2016) | KU764306.1 |
| WI_Ba21 | - | - | *taxus* | 1 | 44.6179495 | -89.989755 | Previously published | Kierepka & Latch (2016) | KU764307.1 |
| WI_Br01 | - | - | *taxus* | 1 | 44.6179495 | -89.989755 | Previously published | Kierepka & Latch (2016) | KU764308.1 |
| WI_Bu01 | - | - | *taxus* | 37 | 44.6179495 | -89.989755 | Previously published | Kierepka & Latch (2016) | KU764309.1 |
| WI_Bu03 | - | - | *taxus* | 37 | 44.6179495 | -89.989755 | Previously published | Kierepka & Latch (2016) | KU764310.1 |
| WI_Ch01 | - | - | *taxus* | 46 | 44.6179495 | -89.989755 | Previously published | Kierepka & Latch (2016) | KU764311.1 |
| WI_Cl01 | - | - | *taxus* | 1 | 44.6179495 | -89.989755 | Previously published | Kierepka & Latch (2016) | KU764312.1 |
| WI_Co02 | - | - | *taxus* | 2 | 44.6179495 | -89.989755 | Previously published | Kierepka & Latch (2016) | KU764313.1 |
| WI_Co03 | - | - | *taxus* | 1 | 44.6179495 | -89.989755 | Previously published | Kierepka & Latch (2016) | KU764314.1 |
| WI_Da02 | - | - | *taxus* | 1 | 44.6179495 | -89.989755 | Previously published | Kierepka & Latch (2016) | KU764315.1 |
| WI_Da05 | - | - | *taxus* | 1 | 44.6179495 | -89.989755 | Previously published | Kierepka & Latch (2016) | KU764316.1 |
| WI_Da08 | - | - | *taxus* | 1 | 44.6179495 | -89.989755 | Previously published | Kierepka & Latch (2016) | KU764317.1 |
| WI_Dg02 | - | - | *taxus* | 2 | 44.6179495 | -89.989755 | Previously published | Kierepka & Latch (2016) | KU764318.1 |
| WI_Fd01 | - | - | *taxus* | 1 | 44.6179495 | -89.989755 | Previously published | Kierepka & Latch (2016) | KU764319.1 |
| WI_Fo02 | - | - | *taxus* | 1 | 44.6179495 | -89.989755 | Previously published | Kierepka & Latch (2016) | KU764320.1 |
| WI_Gl01 | - | - | *taxus* | 1 | 44.6179495 | -89.989755 | Previously published | Kierepka & Latch (2016) | KU764321.1 |
| WI_Gr01 | - | - | *taxus* | 1 | 44.6179495 | -89.989755 | Previously published | Kierepka & Latch (2016) | KU764322.1 |
| WI_Io03 | - | - | *taxus* | 1 | 44.6179495 | -89.989755 | Previously published | Kierepka & Latch (2016) | KU764323.1 |
| WI_Io04 | - | - | *taxus* | 1 | 44.6179495 | -89.989755 | Previously published | Kierepka & Latch (2016) | KU764324.1 |
| WI_Io05 | - | - | *taxus* | 1 | 44.6179495 | -89.989755 | Previously published | Kierepka & Latch (2016) | KU764325.1 |
| WI_Ja01 | - | - | *taxus* | 1 | 44.6179495 | -89.989755 | Previously published | Kierepka & Latch (2016) | KU764326.1 |
| WI_Ja02 | - | - | *taxus* | 1 | 44.6179495 | -89.989755 | Previously published | Kierepka & Latch (2016) | KU764327.1 |
| WI_Ja03 | - | - | *taxus* | 2 | 44.6179495 | -89.989755 | Previously published | Kierepka & Latch (2016) | KU764328.1 |
| WI_Ju02 | - | - | *taxus* | 2 | 44.6179495 | -89.989755 | Previously published | Kierepka & Latch (2016) | KU764329.1 |
| WI_Li01 | - | - | *taxus* | 1 | 44.6179495 | -89.989755 | Previously published | Kierepka & Latch (2016) | KU764330.1 |
| WI_Ma01 | - | - | *taxus* | 1 | 44.6179495 | -89.989755 | Previously published | Kierepka & Latch (2016) | KU764331.1 |
| WI_Ma03 | - | - | *taxus* | 2 | 44.6179495 | -89.989755 | Previously published | Kierepka & Latch (2016) | KU764332.1 |
| WI_Ma04 | - | - | *taxus* | 1 | 44.6179495 | -89.989755 | Previously published | Kierepka & Latch (2016) | KU764333.1 |
| WI_Ma05 | - | - | *taxus* | 1 | 44.6179495 | -89.989755 | Previously published | Kierepka & Latch (2016) | KU764334.1 |
| WI_Ma06 | - | - | *taxus* | 1 | 44.6179495 | -89.989755 | Previously published | Kierepka & Latch (2016) | KU764335.1 |
| WI_Mo01 | - | - | *taxus* | 1 | 44.6179495 | -89.989755 | Previously published | Kierepka & Latch (2016) | KU764336.1 |
| WI_Mt01 | - | - | *taxus* | 1 | 44.6179495 | -89.989755 | Previously published | Kierepka & Latch (2016) | KU764337.1 |
| WI_Mw01 | - | - | *taxus* | 1 | 44.6179495 | -89.989755 | Previously published | Kierepka & Latch (2016) | KU764338.1 |
| WI_Oc02 | - | - | *taxus* | 8 | 44.6179495 | -89.989755 | Previously published | Kierepka & Latch (2016) | KU764339.1 |
| WI_Ou01 | - | - | *taxus* | 1 | 44.6179495 | -89.989755 | Previously published | Kierepka & Latch (2016) | KU764340.1 |
| WI_Ou02 | - | - | *taxus* | 42 | 44.6179495 | -89.989755 | Previously published | Kierepka & Latch (2016) | KU764341.1 |
| WI_Ou03 | - | - | *taxus* | 26 | 44.6179495 | -89.989755 | Previously published | Kierepka & Latch (2016) | KU764342.1 |
| WI_Ou04 | - | - | *taxus* | 1 | 44.6179495 | -89.989755 | Previously published | Kierepka & Latch (2016) | KU764343.1 |
| WI_Ou05 | - | - | *taxus* | 29 | 44.6179495 | -89.989755 | Previously published | Kierepka & Latch (2016) | KU764344.1 |
| WI_Ou06 | - | - | *taxus* | 1 | 44.6179495 | -89.989755 | Previously published | Kierepka & Latch (2016) | KU764345.1 |
| WI_Sa03 | - | - | *taxus* | 1 | 44.6179495 | -89.989755 | Previously published | Kierepka & Latch (2016) | KU764346.1 |
| WI_Sa04 | - | - | *taxus* | 2 | 44.6179495 | -89.989755 | Previously published | Kierepka & Latch (2016) | KU764347.1 |
| WI_Sb01 | - | - | *taxus* | 1 | 44.6179495 | -89.989755 | Previously published | Kierepka & Latch (2016) | KU764348.1 |
| WI_Sb02 | - | - | *taxus* | 1 | 44.6179495 | -89.989755 | Previously published | Kierepka & Latch (2016) | KU764349.1 |
| WI_Sc02 | - | - | *taxus* | 1 | 44.6179495 | -89.989755 | Previously published | Kierepka & Latch (2016) | KU764350.1 |
| WI_Sh01 | - | - | *taxus* | 1 | 44.6179495 | -89.989755 | Previously published | Kierepka & Latch (2016) | KU764351.1 |
| WI_Sh04 | - | - | *taxus* | 1 | 44.6179495 | -89.989755 | Previously published | Kierepka & Latch (2016) | KU764352.1 |
| WI_Sh05 | - | - | *taxus* | 42 | 44.6179495 | -89.989755 | Previously published | Kierepka & Latch (2016) | KU764353.1 |
| WI_Sh06 | - | - | *taxus* | 1 | 44.6179495 | -89.989755 | Previously published | Kierepka & Latch (2016) | KU764354.1 |
| WI_Sh07 | - | - | *taxus* | 2 | 44.6179495 | -89.989755 | Previously published | Kierepka & Latch (2016) | KU764355.1 |
| WI_Sk01 | - | - | *taxus* | 12 | 44.6179495 | -89.989755 | Previously published | Kierepka & Latch (2016) | KU764356.1 |
| WI_SP | - | - | *taxus* | 1 | 44.6179495 | -89.989755 | Previously published | Kierepka & Latch (2016) | KU764357.1 |
| WI_Ve02 | - | - | *taxus* | 1 | 44.6179495 | -89.989755 | Previously published | Kierepka & Latch (2016) | KU764358.1 |
| WI_Wa03 | - | - | *taxus* | 1 | 44.6179495 | -89.989755 | Previously published | Kierepka & Latch (2016) | KU764359.1 |
| WI_Wa04 | - | - | *taxus* | 1 | 44.6179495 | -89.989755 | Previously published | Kierepka & Latch (2016) | KU764360.1 |
| WI_Wa05 | - | - | *taxus* | 2 | 44.6179495 | -89.989755 | Previously published | Kierepka & Latch (2016) | KU764361.1 |
| WI_Wa06 | - | - | *taxus* | 1 | 44.6179495 | -89.989755 | Previously published | Kierepka & Latch (2016) | KU764362.1 |
| WI_Wh02 | - | - | *taxus* | 1 | 44.6179495 | -89.989755 | Previously published | Kierepka & Latch (2016) | KU764363.1 |
| WI_Wk02 | - | - | *taxus* | 1 | 44.6179495 | -89.989755 | Previously published | Kierepka & Latch (2016) | KU764364.1 |
| WI_Wo01 | - | - | *taxus* | 1 | 44.6179495 | -89.989755 | Previously published | Kierepka & Latch (2016) | KU764365.1 |
| WI_Wo02 | - | - | *taxus* | 1 | 44.6179495 | -89.989755 | Previously published | Kierepka & Latch (2016) | KU764366.1 |
| WI_Wo03 | - | - | *taxus* | 1 | 44.6179495 | -89.989755 | Previously published | Kierepka & Latch (2016) | KU764367.1 |
| WI_Wo05 | - | - | *taxus* | 8 | 44.6179495 | -89.989755 | Previously published | Kierepka & Latch (2016) | KU764368.1 |
| WI_Wo06 | - | - | *taxus* | 2 | 44.6179495 | -89.989755 | Previously published | Kierepka & Latch (2016) | KU764369.1 |
| WI_Ws02 | - | - | taxus | 2 | 44.6179495 | -89.989755 | Previously published | Kierepka & Latch (2016) | KU764370.1 |
| WY001 | - | - | *jeffersonii* | 1 | 42.9920352 | -107.55292 | Previously published | Kierepka & Latch (2016) | KU764371.1 |
| WY164 | - | - | *jeffersonii* | 2 | 42.9920352 | -107.55292 | Previously published | Kierepka & Latch (2016) | KU764372.1 |
| WY202 | - | - | *jeffersonii* | 86 | 42.9920352 | -107.55292 | Previously published | Kierepka & Latch (2016) | KU764373.1 |
| WY212 | - | - | *jeffersonii* | 99 | 42.9920352 | -107.55292 | Previously published | Kierepka & Latch (2016) | KU764374.1 |
| WY353 | - | - | *jeffersonii* | 38 | 42.9920352 | -107.55292 | Previously published | Kierepka & Latch (2016) | KU764375.1 |
| WY74 | - | - | *jeffersonii* | 2 | 42.9920352 | -107.55292 | Previously published | Kierepka & Latch (2016) | KU764376.1 |
| WY89 | - | - | *jeffersonii* | 86 | 42.9920352 | -107.55292 | Previously published | Kierepka & Latch (2016) | KU764377.1 |
| WY90 | - | - | *jeffersonii* | 80 | 42.9920352 | -107.55292 | Previously published | Kierepka & Latch (2016) | KU764378.1 |
